# Development of Quality Indicators for the Correct Use of Electronic Medical Records in Primary Care: Modified Delphi Study

**DOI:** 10.2196/80057

**Published:** 2026-01-19

**Authors:** Rico Paridaens, Steve Van den Bulck, Michel De Jonghe, Benjamin Fauquert, Liesbeth Meel, Willem Raat, Bert Vaes

**Affiliations:** 1 Academisch Centrum voor Huisartsgeneeskunde Katholieke Universiteit Leuven (KU Leuven) Leuven, Flanders Belgium; 2 Research Group Healthcare and Ethics Hasselt University Hasselt Belgium; 3 Centre Académique de Médecine Générale Université catholique de Louvain Leuven Belgium; 4 Unité de Recherche en Soins Primaires Vrije Universiteit Brussel Brussels Belgium; 5 Belgian Centre for Evidence-Based Medicine (CEBAM) Leuven Belgium

**Keywords:** electronic medical records, primary care, quality assessment, Delphi method, electronic audit and feedback

## Abstract

**Background:**

When used correctly, electronic medical records (EMRs) can support clinical decision-making, provide information for research, facilitate coordination of care, reduce medical errors, and generate patient health summaries. Studies have reported large differences in the quality of EMR data.

**Objective:**

Our study aimed to develop an evidence-based set of electronically extractable quality indicators (QIs) approved by expert consensus to assess the good use of EMRs by general practitioners (GPs) from a medical perspective.

**Methods:**

The RAND-modified Delphi method was used in this study. The TRIP and MEDLINE databases were searched, and a selection of recommendations was filtered using the specific, measurable, assignable, realistic, and time-bound principles. The panel comprised 12 GPs and 6 EMR developers. The selected recommendations were transformed into QIs as percentages.

**Results:**

A combined list of 20 indicators and 30 recommendations was created from 9 guidelines and 4 review articles. After the consensus round, 20 (100%) indicators and 20 (67%) recommendations were approved by the panel. All 20 recommendations were transformed into QIs. Most (16, 40%) QIs evaluated the completeness and adequacy of the problem list.

**Conclusions:**

This study provided a set of 40 EMR-extractable QIs for the correct use of EMRs in primary care. These QIs can be used to map the completeness of EMRs by setting up an audit and feedback system, and to develop specific (computer-based) training for GPs.

## Introduction

With the advancement of digital technologies, electronic medical records (EMRs) have become the preferred method for recording, storing, and retrieving medical information [1]. The National Alliance for Health Information Technology defined the EMR as “an electronic record of health-related information on an individual that can be created, gathered, managed and consulted by authorised clinicians and staff within one health care organisation” [2,3]. Several studies have shown that the correct use of EMRs may improve the quality and efficiency of care and reduce mortality [4-6]. When used correctly, EMRs can support clinical decision-making (eg, monitoring medication safety) [7-9], facilitate care coordination, reduce medical errors, and provide information for research [1,10]. EMR data can also be used to generate patient health summaries (PHSs). The PHS is a minimum set of clinically relevant data that can be transferred to other health care workers to support the continuity of care and deliver safe and high-quality care to patients [11,12].

Unfortunately, an observational analysis conducted by the Vlaams Agentschap Zorg en Gezondheid, based on approximately 2000 general practitioners’ (GPs) consultations during weekends and holidays in Belgium between 2019 and 2020, revealed that only 32% of patients had a PHS. Several studies have shown that the amount of encoded data registered in EMRs is low (eg, the completeness of height and weight is <76%) [13-16]. Hamade et al [17] suggested that targeting EMR data quality or the use of EMR functions may significantly improve EMR use.

However, there remains a gap in knowledge regarding how to improve the correct use of EMRs [17]. Recently, Ngugi et al [18] developed and validated a set of indicators for the use of EMRs, focusing on the implementation of the EMR, in low- and middle-income countries. There are some guidelines on the good use of EMRs, but none are up to date [19,20]. Appropriate EMR training programs for health care workers and students can improve the use of EMRs; however, an exhaustive list of learning points is lacking [2,21]. Electronic audit and feedback (eA&F) can also be used to improve the correct use of the EMR and, more particularly, the quality of the data stored in the EMR [22]. Ivers et al [23] defined eA&F as “an electronic summary of the clinical performance of health care over a specified period of time aimed at providing information to health professionals to allow them to assess and adjust their performance.” To this end, a validated set of quality indicators (QIs) is used. QIs are measurable items that refer to the structures, processes, and outcomes of care [24]. An evidence-based list of QIs, approved by expert consensus, could help develop appropriate training programs and eA&F interventions to improve EMR use [17,25,26]. This study aimed to develop an evidence-based set of electronically extractable QIs approved by expert consensus to assess the good use of EMRs by GPs from a medical perspective.

## Methods

### Study Design

The RAND-modified Delphi method was used to develop QIs for the use of EMRs from January 2024 to December 2024 based on the method used by Van den Bulck et al [27-29]. The process included the following: (1) development of a questionnaire based on recommendations and existing QIs from evidence-based guidelines and (2) rating procedure by an expert panel in 3 rounds, namely individual written questionnaire, online consensus meeting, and final appraisal.

### Selection of Recommendations and Existing QIs

The TRIP database and MEDLINE were searched for guidelines and indicator sets using the following search terms: “Electronic medical records” AND (“Primary Health Care” OR “Primary Care” OR “General Practice”) AND (“Standard of Care” OR “guideline” OR “recommendation” OR “quality indicator” OR “quality of health care” OR “quality of care”). The search was also extended to Google Scholar (accessed on April 2, 2024). The sources were selected by the primary researcher using the criteria specified in Textbox 1. Sources were not filtered based on the quality of the methodology because of the limited methodological information in any of the sources found in the first search. An exhaustive list of all indicators and recommendations relevant to EMR quality was compiled. For inclusion, indicators and recommendations were filtered using the specific, measurable, acceptable, realistic, and time-bound (SMART) principle [27-29]. A QI is defined by Grypdonck et al as “a measurable tool to assess the quality of care, including outcome-, process- and patient-oriented indicators” [30]. For recommendations, the following definition provided by the World Health Organization was used: “Recommendations are statements designed to help end-users make informed decisions on whether, when and how to undertake specific actions such as clinical interventions, diagnostic tests or public health measures, to achieve the best possible individual or collective health outcomes” [31]. Screening was performed by 2 independent researchers: a GP (primary researcher) and a methodologist. If there was a difference in the results for an indicator or recommendation between the 2 researchers, they discussed it until a consensus was reached. All remaining items (indicators and recommendations) were then categorized into topics according to the organization of the PHS in Belgium as explained by Domus Medica [19], and comparable with categories defined by Jabaaij et al [13] in the EMR scan for general practitioners: completeness and adequacy of the problem list, encoded registration in the EMR, completeness and actuality of medication list, risk factors and drug monitoring, patient identification and contact information, vaccination status, and patients’ choices.

**Selection criteria used to filter articles in the literature.**

**Inclusion criteria**

- Language: English, Dutch, or French
- Evaluation level: microlevel (evaluation at the patient level)
- Source age: indicator sets—any age; recommendations—maximum 10 years
- Target population: primary care providers

**Exclusion criteria**

- Language: other than English, Dutch, or French
- Evaluation level: mesolevel or macrolevel (regional, national, or other)
- Source age: recommendations published more than 10 years ago
- Target population: hospital physicians

### Panel Selection

GPs and EMR developers were invited to participate in the panel via email. Using a purposive sampling technique, priority was given to representing all involved parties at the regional (Flanders, Wallonia, and Brussels) and occupational levels (solo GPs, GPs in group practices, programmers, and academic personnel). The final panel consisted of 18 participants: 11 (61%) Flemish-speaking participants and 7 (39%) French-speaking participants. There were 12 GPs (recently retired: n=1, 6%; worked in a community health center: n=1, 6%; had an individual practice: n=2, 11%; and worked in a group practice: n=8, 44%) and 6 (33%) EMR developers from different EMRs. They were provided with information about the aims and methodology of this study.

### Written Questionnaire Round

#### Completion

An online LimeSurvey questionnaire was presented to a multidisciplinary expert panel (from August 2024 to November 2024) [32]. Each participant was asked to score each item for their capability to measure the quality of EMR use in primary care on a 9-point Likert scale (1=lowest score; 9=highest score). They were asked to base their assessment on the extractability of the indicator or recommendation from the EMR and its possible positive impact on the quality of care. Panel members could also label an item as “not assessable.” At the end of each topic, they were asked to give the top 5 (prioritization) of the items. If a topic did not contain enough items to create the top 5, participants were asked to order half of the indicators in the top X. Finally, all participants could write remarks and add new indicators.

#### Analysis

The results of the online survey were analyzed using a Node.js (OpenJS Foundation) script to subdivide each item into 3 categories: high, uncertain, or low potential as a QI [33]. The script generated a report for each participant with their responses, a general overview of the calculated parameters (Table 1), and a category based on these parameters (Table 2).

**Table 1:** Defined parameters to score each quality indicator and recommendation [28].

Parameter		Calculation
Total number of responses		N responses
**Likert score**		
	For each score, the total number of responses	N responses for score
	Median Likert score	Median Likert score of all responses
	Highest ratings (%)	N responses with a score ≥7 divided by the total number of responses
Prioritization (top %)		Sum of the individual top X scores (eg, first place=score 3, second place=score 2, third place=score 1, and items not ranked in the top 3=score 0) divided by the maximum possible score (N responses × highest possible top score)

**Table 2:** Selection criteria for indicators and recommendations [28].

Criterion	Selection^a^	Discussion^a^	No selection^a^
Likert score: median	≥7	Any	Any
Likert score: distribution	≥70% with score ≥7	≥30% with score <3	Other
Prioritization (top %)	≥20%	≥1%	<1%

^a^Indicators and recommendations must fulfill all criteria, not only some, to be categorized as either “selection,” “discussion,” or “no selection.”

### Consensus Meeting Round

Each panel member received an individual feedback report 2 weeks before the consensus meeting. It also included new recommendations and comments. The report contained all items with a color code representing their potential to reach a consensus (Multimedia Appendix 1).

An online consensus meeting was held using Microsoft Teams [34]. To improve the efficiency of the consensus meeting, focus was given to the items categorized as “uncertain,” together with the newly added indicators proposed by the panelists. Items with high potential were automatically selected for the final selection unless panel members requested a decision-making discussion. Items with low potential were excluded from the final selection unless the panel members requested deliberation.

All accepted indicators and recommendations were adjusted to the conclusions of the consensus meeting and conformed to the SMART criteria [27-29]. The recommendations were transformed into QIs as percentages. The final report was sent to the panel members for final acceptance in Dutch and French. The final indicator set was translated into English by the researchers.

### Ethical Considerations

This study was approved on June 25, 2024, by the Social and Societal Ethics Committee of KU Leuven (G-2024-8020-R2(AMD)). The project was also vetted and approved by KU Leuven’s Privacy and Ethics platform in view of the principles and obligations laid down in the General Data Protection Regulation of the European Parliament and the Council of 27 April 2016 [35]. Participants were asked to digitally sign an informed consent form before participation. All data were pseudonymized after the written questionnaire round using a unique identifier. Personal information was separated from other research data in a second file with password protection. The personal data was used to provide the participants the feedback report, to invite them for the consensus meeting round, and to approve the final report. All personal information was deleted after the study was finished. No compensation was provided to the participants.

## Results

### Extraction of QI and Recommendations

**Figure 1:**
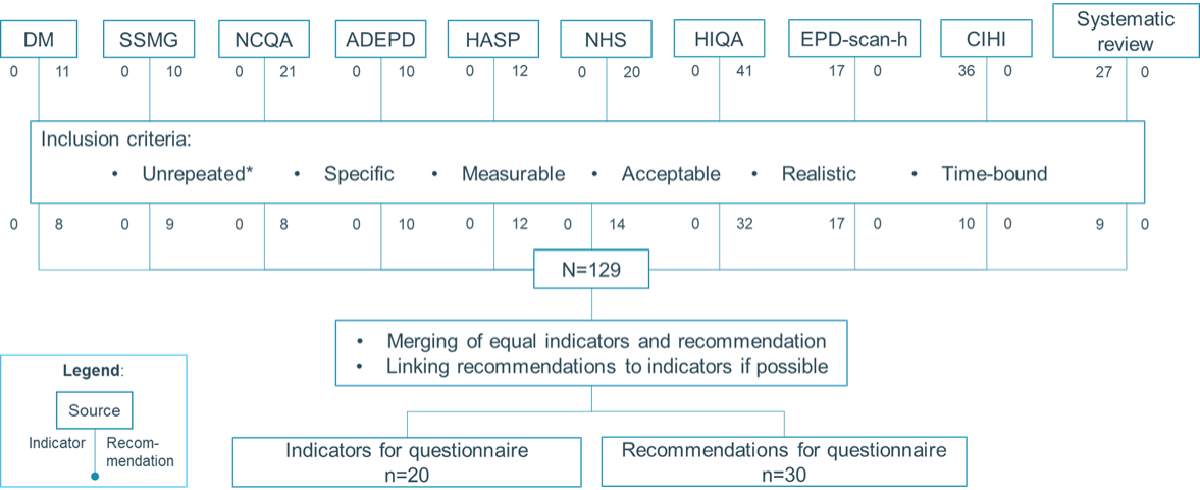
Overview of the recommendation and indicator extraction. *Unique: repetitive recommendations were removed. ADEPD: NHG-Richtlijn Adequate dossiervorming met het elektronisch patiëntdossier; CIHI: Canadian Institute for Health Information; DM: Domus Medica; HASP: NHG Richtlijn Informatie-uitwisseling tussen huisarts en medisch specialist; HIQA: Health Information and Quality Authority; NCQA: National Committee for Quality Assurance; NHS: National Health Service; SSMG: Société Scientifique de Médecine Générale [11,14,16,20,21,38-44].

A total of 4 review articles listed QIs for the use of EMRs, containing 27 QIs [13,15,36-38]. Nine guidelines contained 53 QIs and 125 recommendations [11,19,20,39-42]. Where possible, duplicate items were removed, and indicators were linked to similar recommendations in the guidelines. This resulted in a combined list of 20 QIs and 30 recommendations (Figure 1 [11,13,15,19,20,36-42]; Multimedia Appendices 2-4).

### Questionnaire Round

All panel members (18/18, 100%) completed the questionnaire. On the basis of these categories, out of 50 items, 21 (42%) were selected, 27 (54%) required further discussion, and 2 (4%) were excluded.

### Consensus Round

Of the 18 panel members, 13 (72%) were present for the consensus meeting—9 (50%) GPs and 4 (22%) EMR developers—8 (44%) of whom were Flemish speaking and 5 (28%) French speaking. In the panel round, none of the preselected indicators (n=7) or recommendations (n=14) were discarded. Of the 10 indicators labeled as “discussion,” 7 (70%) were selected, and 1 (10%) indicator was further divided into 7 specific indicators (Multimedia Appendix 1; indicator 3). Of the 17 recommendations labeled as “discussion,” 6 (35%) were selected. This resulted in a list of 20 indicators and 20 recommendations (Multimedia Appendix 1). During the consensus meeting, some items that were not yet extractable were included because the panel members felt that their clinical importance should prompt EMR providers to adapt their software.

### Final Evaluation

The 20 recommendations were transformed into QIs and added to the 20 other QIs, resulting in 16 (40%) QIs regarding the completeness and adequacy of the problem list, 4 (10%) QIs regarding encoded registration in the EMR, 3 (8%) QIs regarding the completeness and actuality of the medication list, 6 (15%) QIs regarding risk factors and drug monitoring, 4 (10%) QIs regarding patient identification and contact information, 3 (8%) QIs regarding vaccination status, and 4 (10%) QIs regarding patient choices (Textbox 2; Multimedia Appendix 5). All panel members present in the consensus meeting (13/13, 100%) approved the final report.

**Quality indicators for the good use of electronic medical records (EMRs) in primary care.**

**Topic 1: completeness and adequacy of the problem list**

1.1 The mean of active problem items listed on a patient’s problem list

1.2 Percentage of items on the problem list that are linked to an encoded diagnosis

1.3 Percentage of patients with medication for thyroid disease in their medication list with an encoded diagnosis of thyroid disease on the problem list

1.4 Percentage of patients with medication for epilepsy in their medication list with an encoded diagnosis of epilepsy on the problem list

1.5 Percentage of patients with medication for Parkinson disease in their medication list with an encoded diagnosis of Parkinson disease on the problem list

1.6 Percentage of patients with medication for depression in their medication list with an encoded diagnosis of depression on the problem list

1.7 Percentage of patients with medication for cardiovascular disease in their medication list with an encoded diagnosis of cardiovascular disease on the problem list

1.8 Percentage of patients with medication for asthma or chronic obstructive pulmonary disease in their medication list with an encoded diagnosis of asthma or chronic obstructive pulmonary disease on the problem list

1.9 Percentage of patients with medication for diabetes in their medication list with an encoded diagnosis of diabetes on the problem list

1.10 Percentage of encoded items in the problem list with an additional description or comment

1.11 Percentage of encoded items in the problem list for which the estimated or actual date of onset has been registered

1.12 Percentage of inactive items on the problem list for which the estimated or actual date the condition was resolved has been registered

1.13 Percentage of surgeries and other procedures that have been registered as an encoded item

1.14 Percentage of surgeries and other procedures in the patient history that have been linked to the related episode of care

1.15 Percentage of surgeries and other procedures in the patient’s history with an additional description or comment

1.16 Percentage of surgeries and other procedures in the patient summary for which the date and/or time on which the procedure was or is intended to be performed is registered

**Topic 2: encoded registration in the EMR**

2.1 The mean number of subcontacts created per contact during the observation period

2.2 Percentage of subcontacts during the observation period connected with an episode of care

2.3 Percentage of subcontacts (registered as consultation, home visit, or teleconsultation) with a subjective, objective, evaluation, and planning item

2.4 The number of patients with a global medical record in the practice with changes in the status of tasks (procedures or actions) over the total number of patients with a global medical record in the practice

**Topic 3: completeness and actuality of the medication list**

3.1 Percentage of medications on the medication list labeled as *active* that are no longer active

3.2 Percentage of active medications on the medication list that are linked to a health condition on the problem list

3.3 Percentage of active medications on the medication list for which the dosage and treatment regimen are complete

**Topic 4: risk factors and drug monitoring**

4.1 Percentage of patients with at least 1 registration for a drug allergy or intolerance

4.2 Percentage of patients with a registration for a risk factor; risk factors include clinical information that is imperative to know so that the life or health of the patient is not threatened, for example, prophylaxis for adrenal crisis (Addison disease), endocarditis, bleeding disorders, endoprosthesis, patients who are immunocompromised, (functional) asplenia, thrombosis, or particularly resistant microorganisms

4.3 Percentage of patients with at least 1 encoded registration for a physical examination (weight, height, blood pressure, and pulse)

4.4 Percentage of patients with at least 1 registration of an item for social history

4.5 Percentage of patients aged 12 years and older for whom there is at least 1 notation concerning the use of cigarettes, alcohol, and substances

4.6 Percentage of patients with at least 1 registration of an item for family medical history

**Topic 5: patient identification and contact information**

5.1 Percentage of patients with a registration for hospital preference

5.2 Percentage of patients with at least 1 registration of contact information for a contact person, caregiver, or person designated as representative

5.3 Percentage of patients with at least 1 registration of a health care professional in their care team

5.4 Percentage of patients with at least 1 registration for address, email, employer, home and work phone, marital status, registration status on patient platform (eg, Helena [43]), or cohousing people and animals

**Topic 6: vaccination status**

6.1 Percentage of patients aged 7 years and older who have a registration for all the recommended primary childhood vaccines

6.2 Percentage of patients aged 65 years and older who have a registration for a yearly influenza vaccine

6.3 Percentage of vaccinations for which the date the vaccination was administered is registered

**Topic 7: patient choices**

7.1 Percentage of patients with a registration of treatment preferences: euthanasia request or refusal for intubation, resuscitation, organ donation, vaccination, or blood transfusion

7.2 Percentage of patients with at least 1 contact with a subjective, objective, evaluation, or planning item linked to an encoded diagnosis regarding treatment preferences or end-of-life care

7.3 Percentage of patients with at least 1 registration of a goal for personalized care (goal-oriented care)

7.4 Percentage of patients with at least 1 registration regarding preferences for preventive health care

## Discussion

### Principal Findings and Comparison With Prior Work

This study used the RAND-modified Delphi method to validate a set of 40 QIs for the correct use of EMRs by GPs based on the indicators and recommendations found in the literature. These indicators are (potentially) EMR extractable and evaluate key aspects of EMR use by GPs: completeness and adequacy of the problem list, encoded registration, completeness and actuality of the medication list, risk factors and drug monitoring, patient identification and contact information, vaccination status, and patient choices. Using the SMART principle, we ensured that the QIs could be evaluated in clinical practice [27-29,44-46].

Previously, Jabaaij et al [38] developed the EMR scan for general practitioners in the Netherlands to evaluate EMR use. However, the method they used to define QIs was unclear. Ngugi et al [18] developed and validated a set of indicators for the use of EMRs in low- and middle-income countries. Their QIs mostly evaluated the general use of the EMRs (eg, indicator 7, “% of required data elements contained in EHRs”). Our study focuses on how data are registered and encoded in EMRs, focusing on which data are registered [18]. Other indicator sets were either outdated (aged >20 years) [36], focused on clinical performance instead of use of the EMR [15], or evaluated EMR use on the mesolevel or macrolevel instead of the microlevel [37]. Hamade et al [17] noted that while there are many studies on the implementation of EMR, there is a scarcity of studies on its use.

Most of the selected indicators evaluated the completeness and adequacy of the problem list. The problem list provides important information for decision-making [47]. Our indicators are primarily process indicators because they focus on the process of EMR registration (ie, use). However, these QIs cannot be directly linked to patient outcomes. Process indicators are useful for quality assessment. They are more sensitive to differences in quality than outcome measures and are easier to interpret [48]. Recent literature shows an interest in eA&F [22,29], dashboard interface features to support reflection on practice (“barometers”) [27-29], and computer-based decision support in primary care [49]. The quality of data in EMRs impacts the effectiveness of these systems. Several studies have shown that improving data quality may improve the quality and efficiency of care and reduce mortality [4-6]. Unfortunately, data show that physicians spend approximately two-thirds of their work time interacting with EMRs in their offices. This administrative burden negatively impacts clinicians’ wellness, leading to an increased chance of burnout. Recently, the interest in the use of artificial intelligence for analyzing data, identifying hidden information, identifying risks, and providing suggestions for diagnosis has increased rapidly. This can greatly facilitate the process for GPs to easily improve data quality without spending more time entering data in the EMR and less time interacting with patients [50]. GPs should not need to adapt their working habits to the capabilities of their medical software; rather, EMRs should be adapted to the needs of GPs to facilitate the correct registration of data [51]. An important lever to encourage EMR developers to align their software more closely with clinicians’ data entry and retrieval needs lies in regulatory frameworks, such as homologation. This is a certification procedure to evaluate the mandatory standards and certification requirements that EMRs must fulfill to be approved for use within the health care system [52]. By embedding QIs derived through expert consensus, such as those developed in this study, into these frameworks, regulators can create explicit performance benchmarks that EMRs must meet. This alignment incentivizes developers to design functionalities that facilitate accurate, complete, and efficient data capture and reporting by clinicians [50].

### Strengths and Limitations

Our panel included members working in all parts of Belgium and in all types of practices, giving it broad support. Because our panel included health professionals and EMR developers, we were able to define clinically relevant indicators that considered the technical requirements for extractability. During the consensus meeting, the EMR developers emphasized that these indicators could possibly stimulate GPs to use the features they have already developed more extensively. We are convinced that their opinion was a valuable addition to the panel. EMR developers were the most capable of assessing whether a QI could be automatically extracted from the EHR data. However, we cannot exclude a potential bias caused by their inclusion (eg, the ease or cost of addressing a recommended QI outweighing the importance of making that QI available from a public health perspective). The scarcity of literature on the initial set of QIs and recommendations is a double-edged sword for our study. On the one hand, it limits the strengths of the initial set of QIs and recommendations because none of our sources used a systematic approach to evaluate the validity of their QIs and recommendations, had a well-explained methodology [13,15,36-38], or could be evaluated using the Appraisal of Guidelines for Research and Evaluation II method [11,19,20,39-42]. On the other hand, this allowed us to conduct a broad and general search on the topic, which could increase its universal applicability. There are many differences between countries in terms of how and which data are registered [11,19,20,39-42]. We attempted to generalize the QIs and recommendations as much as possible. However, adaptation to the local context is necessary. Another important limitation is that some indicators are not currently extractable. However, as mentioned earlier, these indicators reflect aspects of clinical practice that are important enough to warrant adaptation of EMRs to include this information.

### Future Research Directions

This set of indicators is important for future research on EMR use. Developing a list of QIs is the first step in implementing a strategy and realizing a quality loop. The next essential step is to conduct practice testing for operational validity [29]. These results can be used to create an eA&F system [22,23]. This set of QIs can also be used to develop specific (computer based) training for GPs based on a set of learning goals [2]. It should be noted that our QIs can be used for the correct use of EMRs but cannot be used to quantify the quality of care provided by GPs. Further research is necessary to evaluate whether the correct use of the EMR, as proposed in this set of QIs, also affects the quality of care provided to patients and its possible effect on the data in the PHS.

### Conclusions

This study provided a set of 40 EMR-extractable QIs for the correct use of EMRs in primary care based on international guidelines and approved by GPs and EMR developers. These QIs can be used as a framework to measure and improve the quality and completeness of EMRs in primary care.

## Appendix

Multimedia Appendix 1 Example of the feedback report for panel members.

Multimedia Appendix 2 Online questionnaire - Dutch version.

Multimedia Appendix 3 Online questionnaire - French version.

Multimedia Appendix 4 Online questionnaire - English version (automated generated translation by DeepL Translator; https://www.deepl.com/nl/translator).

Multimedia Appendix 5 Final indicator set.

## Abbreviations

eA&F: electronic audit and feedback
EMR: electronic medical record
GP: general practitioner
PHS: patient health summary
QI: quality indicator
SMART: specific, measurable, assignable, realistic, and time-bound

## Data Availability

The datasets generated or analyzed during this study are available from the corresponding author on reasonable request.
