# Supplementary material for: Development of Quality Indicators for the Correct Use of Electronic Medical Records in Primary Care: Modified Delphi Study
Source: JMIR Med Inform. 2026 Jan 19;14:e80057. doi: 10.2196/80057 (PMC12865340; doi:10.2196/80057)
Supplement: Multimedia Appendix 1 [file medinform_v14i1e80057_app1.pdf]

INTEGO Indicatorset: Correct gebruik elektronisch patiëntendossier bij huisartsen / Le bon usage du dossier électronique du patient en médecine générale

Topic 1. Volledigheid en adequaatheid probleemlijst / 1. Exhaustivité et adéquation de la liste des problèmes

#	Type	Description		Total	Likert score											Rank % top 5	Potential	Agreement	Conclusion
		Nl	Fr		Lowest			Middle			Highest			% Highest	Median				
					1	2	3	4	5	6	7	8	9						
1	IND	Hoeveel actieve zorgelementen heeft een patiënt gemiddeld op zijn/haar lijst met zorgelementen?	Combien d'éléments de santé/soins actifs un patient a en moyenne sur sa liste de éléments de santé/soins?<br>Erreur dans equète: Noté comme indicateur Topic 2 Indicateur 1. Corretement ici	17	0	1	1	1	2	2	2	7	1	59	7	11	Uncertain	Uncertain	**Discussion**
2	IND	Hoeveel procent van de probleemstatuswaardige episodes heeft inderdaad het label ‘bijzondere attentiewaarde’?	Quel est le pourcentage d'élément de santé/soins avec une attention particulière qui sont effectivement étiquetés "valeur d'attention particulière"?	18	0	2	1	3	0	2	6	2	2	56	7	18	Uncertain	Uncertain	**Discussion**
3	IND	Een aantal medicamenten worden voorgeschreven specifiek in het kader van een bepaalde ziekte. Voor patiënten met een voorschrift voor deze medicatie dient een zorgelement met die diagnose aanwezig te zijn. Afwezigheid hiervan kan wijzen op gebrek van registratie van dit zorgelement in het EPD. Deze aandoeningen zijn:<br>• schildklierlijden,<br>• epilepsie,<br>• ziekte van Parkinson,<br>• depressie,<br>• cardiovasculaire ziekte,<br>• astma/COPD,<br>• diabetes	Un certain nombre de médicaments sont prescrits spécifiquement dans le contexte d'une maladie particulière. Pour les patients ayant une ordonnance pour ces médicaments, un élément de soins avec ce diagnostic doit être présent. L'absence d'un tel élément peut indiquer qu'il n'a pas été enregistré dans le DMI.<br>Ces maladies sont les suivantes:<br>• maladie thyroïdienne,<br>• épilepsie,<br>• la maladie de Parkinson,<br>• la dépression,	18	0	0	1	0	0	2	1	10	4	83	8	43	High	Yes	**Selection**

#	Type	Description		Total	Likert score											Rank % top 5	Potential	Agreement	Conclusion
		Nl	Fr		Lowest			Middle			Highest			% Highest	Median				
					1	2	3	4	5	6	7	8	9						
			• maladie cardiovasculaire,<br>• asthme/COPD,<br>• diabète.																
4	IND	Percentage van geregisteerde patiënten waarvoor er geen aanpassing was in het EPD over de voorbije 12 maanden.<br>Voorstel aanpassing: en WEL een contact was.<br>De periode dat er geen aanpassing was in het EPD zou eventueel verlengd kunnen worden	Pourcentage de patients enregistrés pour lesquels il n'y a pas de changement dans le DMI les 12 derniers mois.<br>Proposition d'adaption aanpassing: et AVEC un contact.<br>La période pendant laquelle il n'y a pas eu d'ajustement dans le DMI pourrait éventuellement être prolongée	17	0	2	4	2	2	1	4	1	1	35	5	11	Low	No	**No selection**
5	IND	Percentage van de patiëntenpopulatie, 18 jaar en ouder, met chronische gezondheidsproblemen die ten minste een van de volgende soorten zelfmanagementondersteuning hebben ontvangen van hun zorgverlener in de eerstelijnsgezondheidszorg:<br>• voorzien van een behandelplan ;<br>• aangemoedigd om zelfhulpgroepen of -programma's te gebruiken.	Pourcentage de la population de patients, âgés de 18 ans et plus, atteints de maladies chroniques qui ont reçu au moins un des types suivants de soutien à l'autogestion de la part de leur prestataire de soins de santé primaires:<br>• fourni un plan de traitement;<br>• encouragé à utiliser des groupes ou des programmes d'entraide	18	0	5	1	5	3	1	1	1	1	17	4	4	Uncertain	Uncertain	**Discussion**
6	IND	Hoeveel procent van de episodes op de episodelijst heeft een correcte ICPC-code?<br>Voorstel aanpassing: 'gecodeerde' episode ipv 'correcte' ICPC-code	Quel est le pourcentage d'élément de santé/soins de la liste d'élément de santé/soins ayant un code ICPC correcte?<br>Adaption proposée : épisode « codé » au lieu de code ICPC « correcte ».	17	2	0	0	0	0	1	4	7	3	82	8	31	High	Yes	**Selection**

#	Type	Description		Total	Likert score											Rank % top 5	Potential	Agreement	Conclusion
		Nl	Fr		Lowest			Middle			Highest			% Highest	Median				
					1	2	3	4	5	6	7	8	9						
7	REC	Het is aangewezen dat communicatiebeperkingen geregistreerd staan in het EPD opdat de zorgverlener hiermee rekening kan houden.	Il convient que les restrictions de communication soient enregistrées dans le DMI afin que le prestataire de soins puisse en tenir compte.	18	0	0	1	0	5	2	3	7	0	56	7	6	Uncertain	Uncertain	**Discussion**
8	REC	In het EPD wordt bijkomende informatie betreffende de diagnose best vastgelegd in bijschrift of commentaar welke meer info geeft over de aspecten van de aandoening.	Dans le DMI, il est préférable d'enregistrer les informations supplémentaires concernant le diagnostic dans une légende ou un commentaire qui donne plus d'informations sur les aspects de la maladie.	18	0	0	0	2	2	0	2	7	5	78	8	27	High	Yes	**Selection**
9	REC	In het EPD dient de startdatum vastgelegd te worden van ieder zorgelement.	Le DMI doit enregistrer la date de début de chaque élément de soins.	16	0	0	0	0	1	3	2	5	5	75	8	25	High	Yes	**Selection**
10	REC	In het EPD dient de einddatum of vermoedelijke einddatum vastgelegd te worden van ieder afgesloten zorgelement.	Le DMI doit enregistrer la date de fin ou la date de fin présumée de chaque élément de soins terminé.	17	0	1	0	0	1	4	5	5	1	65	7	16	Uncertain	Uncertain	**Discussion**
11	REC	In het EPD wordt dient geregistreerd te worden dat de patiënt geen gekende ziektes heeft indien de patiënt geen gekende ziektes heeft. Op deze manier weten andere zorgverleners dat het dossier volledig is. (ICPC-2: A97)	Elle doit être enregistrée dans le DMI si le patient n'a pas de maladie connue. De cette manière, les autres prestataires de soins de santé savent que le dossier est complet. (ICPC-2 : A97)	18	1	2	0	2	4	5	2	1	1	22	5.5	3	Uncertain	Uncertain	**Discussion**
12	REC	Het EPD bevat: alle aandoeningen of gegevens die van belang zijn voor de verdere hulpverlening, zoals bepaalde ingrepen en aandoeningen die kunnen recidiveren. Verduidelijking: het gaat hier over operaties en belangrijke behandelingen zoals omschreven in NHG Ingrepenviewer	Le DMI contient toute condition ou donnée pertinente pour la suite des soins, telle que certaines procédures et conditions susceptibles de se reproduire. Clarification : il s'agit des interventions chirurgicales et des traitements majeurs tels que définis dans le NHG Ingrepenviewer"	18	0	0	0	2	1	0	1	12	2	83	8	32	High	Yes	**Selection**

#	Type	Description		Total	Likert score											Rank % top 5	Potential	Agreement	Conclusion
		Nl	Fr		Lowest			Middle			Highest			% Highest	Median				
					1	2	3	4	5	6	7	8	9						
13	REC	Leg operaties en belangrijke behandelingen bij voorkeur vast bij de relevante episode. Doe dit met de functionaliteit van uw EMD. Daarmee komt deze informatie ook in het overzicht Ingrepen en behandelingen terecht.	Leg operaties en belangrijke behandelingen bij voorkeur vast bij de relevante episode. Doe dit met de functionaliteit van uw HIS. Daarmee komt deze informatie ook in het overzicht Ingrepen en behandelingen terecht.	17	0	0	1	0	0	3	6	6	1	76	7	25	High	Yes	**Selection**
14	REC	Het EPD bevat voor relevante operaties en ingrepen een bijschrift met informatie over de procedure.	Pour les opérations et procédures concernées, le DMI contient une légende avec des informations sur la procédure.	17	1	1	1	3	2	3	1	4	1	35	6	21	Uncertain	Uncertain	**Discussion**
15	REC	Het EPD bevat voor relevante operaties en ingrepen de datum waarop deze is uitgevoerd.	Pour les opérations et procédures pertinentes, le DMI contient la date à laquelle elles ont été effectuées.	17	0	0	0	0	0	1	2	10	4	94	8	26	High	Yes	**Selection**
16	REC	Het EPD bevat een registratie indien de patiënt geen operaties of ingrepen in het verleden gehad heeft.	Le DMI contient un enregistrement si le patient n'a pas subi d'opérations ou de procédures antérieures.	17	2	2	1	1	6	0	3	1	1	29	5	13	Uncertain	Uncertain	**Discussion**

Topic 2. Gestructureerd registreren in EPD / 2. Enregistrement structuré dans le DMI

#	Type	Description		Total	Likert score											Rank % top 3	Potential	Agreement	Conclusion
		Nl	Fr		Lowest			Middle			Highest			% Highest	Median				
					1	2	3	4	5	6	7	8	9						
1	IND	Hoeveel procent van de deelcontacten in het journaal gekoppeld aan zorgelementen met een geldige ICPC-code? Voorstel aanpassing: 'gecodeerde' episode ipv 'correcte' ICPC-code	Quel est le pourcentage de sous-contacts dans le journal connecté avecdes éléments de soins/santé avec un code ICPC valide? Adaption proposée : épisode « codé » au lieu de code ICPC « correcte ».	18	0	1	0	0	1	2	4	6	4	78	8	43	High	Yes	**Selection**
2	IND	Hoeveel procent van de deelcontacten die zijn vastgelegd tijdens consulten, telefonische contacten en huisbezoeken hebben een Subjectief-, Objectief-, Evaluatie- of Planning-regel?	Quel est le pourcentage de contacts partiels enregistrés lors de consultations, de contacts téléphoniques et de visites à domicile qui comportent une ligne Subjectif, Objectif, Evaluation ou Planning?	18	0	1	0	0	4	2	5	4	2	61	7	69	Uncertain	Uncertain	**Discussion**
3	IND	Aantal raadplegingen voor online bronnen (vb. CDLH)	Nombre de consultations pour les sources online (par example. CDLH)	18	3	0	1	2	4	3	1	3	1	28	5	33	Uncertain	Uncertain	**Discussion**
4	IND	Aantal veranderingen in planningen per week per 1000 GMD patiënten	Nombre de changements d'horaires par semaine pour 1000 patients avec dossier médical global	16	3	0	3	0	3	2	3	1	1	31	5	27	Uncertain	No	**Discussion**
5	REC	Consultation, laboratory and imaging reports filed in the chart are initialed by the practitioner who ordered them, to signify review. (Review and signature by professionals other than the ordering practitioner do not meet this requirement.) If the reports are presented electronically or by some other method, there is also representation of review by the ordering practitioner. Consultation and abnormal laboratory and imaging study results have an explicit notation in the record of followup plans.	Consultation, laboratory and imaging reports filed in the chart are initialed by the practitioner who ordered them, to signify review. (Review and signature by professionals other than the ordering practitioner do not meet this requirement.) If the reports are presented electronically or by some other method, there is also representation of review by the ordering practitioner. Consultation and abnormal laboratory and imaging study results have an explicit notation in the record of followup plans.	17	1	3	2	1	3	1	1	4	1	35	5	25	Uncertain	No	**Discussion**

Topic 3. Compleetheid en actualiteit medicatie-overzicht / 3. Exhaustivité et actualisation de la liste des médicaments

#	Type	Description		Total	Likert score											Rank % top 3	Potential	Agreement	Conclusion
		Nl	Fr		Lowest			Middle			Highest			% Highest	Median				
					1	2	3	4	5	6	7	8	9						
1	IND	Hoeveel procent van de medicatie op de lijst 'actuele medicatie' is onterecht als actuele medicatie gelabeld?	Quel est le pourcentage des médicaments sur la liste des « médicaments actuels » qui sont incorrectement étiquetées comme étant des médicaments actuels?	18	0	0	1	0	2	1	0	11	3	78	8	56	High	Yes	**Selection**
2	IND	Hoeveel procent van de voorschriften is gekoppeld aan een episode?	Quel est le pourcentage de prescriptions connecté avec un épisode de soins/santé?	18	0	2	1	2	0	1	5	7	0	67	7	54	Uncertain	Uncertain	**Discussion**
3	IND	Volledige dosis- en behandelingsschema met betrekking tot dosis-effect of bijwerkingen van medicatie.	Détails complets sur la dose et le régime concernant l'effet de la dose ou les réactions indésirables aux médicaments.	18	0	0	0	0	0	2	3	9	4	89	8	57	High	Yes	**Selection**
4	IND	Aantal voorschriften voor medicatie per week per 1 000 GMD patiënten?	Nombre de prescriptions de médicaments par semaine pour 1 000 patients avec dossier médical global?	17	1	2	1	2	2	3	3	3	0	35	6	14	Uncertain	Uncertain	**Discussion**
5	IND	Indien de patiënt geen medicatie neemt dient dit correct genoteerd te zijn in het EPD.	Si le patient ne prend pas de médicaments, cela doit être correctement noté dans le DMI.	18	2	2	3	2	2	3	3	0	1	22	4.5	20	Uncertain	Uncertain	**Discussion**

Topic 4. Risicofactoren / Medicatiebewaking / 4. Facteurs de risque / surveillance des médicaments

#	Type	Description		Total	Likert score											Rank % top 5	Potential	Agreement	Conclusion
		Nl	Fr		Lowest			Middle			Highest			% Highest	Median				
					1	2	3	4	5	6	7	8	9						
1	IND	Aantal nieuwe planningen voor screening voor dikkedarmkanker of borstkanker per week per 1000 GMD patiënten	Nombre de nouveaux programmes de dépistage du cancer colorectal ou du cancer du sein par semaine pour 1000 patients avec dossier médical global	17	1	1	1	0	1	3	4	5	1	59	7	34	Uncertain	Uncertain	**Discussion**
2	IND	Van hoeveel patiënten is een contra-indicatie medicatie geregistreerd? Contra-indicaties zijn aandoeningen waarmee bij het voorschrijven van medicatie rekening dient te worden gehouden. Te denken valt aan bijvoorbeeld diabetes mellitus of nierinsufficiëntie. Het kan ook gaan om tijdelijke contra-indicaties zoals bijvoorbeeld bij zwangerschap.	Combien de patients ont des médicaments contre-indiqués enregistrés ? Les contre-indications sont des conditions qui doivent être prises en compte lors de la prescription de médicaments. Il s'agit par exemple du diabète sucré ou de l'insuffisance rénale. Il peut également s'agir de contre-indications temporaires telles que la grossesse.	18	1	0	1	0	2	2	6	3	3	67	7	27	Uncertain	Uncertain	**Discussion**
3	IND	Van hoeveel patiënten is een geneesmiddelenallergie of -intolerantie geregistreerd?	Pour combien de patients une allergie ou une intolérance aux médicaments a-t-elle été enregistrée ?	18	0	0	1	0	1	0	4	8	4	89	8	30	High	Yes	**Selection**
4	REC	Bepaalde medische informatie is van belang voor de hele zorgketen. NHG beveelt registratie aan van: profylaxe voor addison crisis, endocarditis, bloedingsziekten, endoprothese, immuungecompromitteerde patiënt, (functionele) asplenie, trombose of bijzonder resistente micro-organismen.	Certaines informations médicales sont importantes pour l'ensemble de la chaîne de soins. Le NHG recommande l'enregistrement des informations suivantes : prophylaxie de la crise d'Addison, endocardite, maladies hémorragiques, endoprothèse, patient immunodéprimé, asplénie (fonctionnelle), thrombose ou micro-organismes particulièrement résistants.	17	0	1	0	1	1	2	3	7	2	71	8	22	High	Yes	**Selection**

#	Type	Description		Total	Likert score											Rank % top 5	Potential	Agreement	Conclusion
		Nl	Fr		Lowest			Middle			Highest			% Highest	Median				
					1	2	3	4	5	6	7	8	9						
5	REC	Lichamelijk onderzoek kan in vrije tekst of als diagnostische bepaling in het EPD worden vastgelegd. Indien het als diagnostische bepaling is vastgelegd, kan het worden hergebruikt voor overzichten in de tijd en voor beslisondersteuning. Waar mogelijk dient het lichamelijk onderzoek dan ook zo veel mogelijk als diagnostische bepaling worden vastgelegd in het EPD.<br>Diagnostische bepalingen zijn bijvoorbeeld: gewicht, lengte, bloeddruk, hartritme, gebruik van alcohol en nicotine, middelengebruik, hoeveelheid lichaamsbeweging...<br>Een volledige lijst kan u vinden op: NHG - Bepalingenviewer	L'examen physique peut être enregistré en texte libre ou en tant que diagnostic dans le DMI. S'il est enregistré en tant que diagnostic, il peut être réutilisé pour des aperçus dans le temps et pour l'aide à la décision. Dans la mesure du possible, l'examen physique doit donc être enregistré en tant que diagnostic dans le DMI.<br>Examples des diagnostic: le poids, la taille, la tension artérielle, le rythme cardiaque, la consommation d'alcool et de nicotine, la consommation de substances, l'intensité de l'activité physique....<br>Une liste complète peut être consultée à l'adresse suivante: NHG - Bepalingenviewer"<br>Proposition correction: enregistré sous forme structurée	17	1	0	0	0	2	0	2	9	3	82	8	39	High	Yes	**Selection**
6	REC	Voor alle patiënten 12 jaar en ouder is een een vermelding in het EMD in verband met het gebruik van cigaretten, alcohol en middelen.<br>><br>Voorstel aanpassing: dit moet jaarlijks geregistreerd worden.	Pour tous les patients de 12 ans et plus, est notée dans le DMI si le patient utilise des cigarettes, d'alcool et de substances.<br>Propositon d'adaption: ceci devrait être recodé chaque année.	18	0	0	0	2	0	3	2	3	8	72	8	37	High	Yes	**Selection**
7	REC	Indien in het EPD het dossierdeel Familieanamnese aanwezig is:<br>• leg hierin de informatie vast over de aandoeningen die voorkomen bij bloedverwanten, per aandoening bij welke familieleden deze voorkomt, op welke leeftijd	Si la section "Anamnèse familiale" est présente dans le DMI:<br>• enregistrez ici les informations sur les troubles qui surviennent chez les membres de la famille, pour chaque trouble	17	0	0	1	0	2	4	5	4	1	59	7	31	Uncertain	Uncertain	**Discussion**

#	Type	Description		Total	Likert score											Rank % top 5	Potential	Agreement	Conclusion
		Nl	Fr		Lowest			Middle			Highest			% Highest	Median				
					1	2	3	4	5	6	7	8	9						
		het bij hen is begonnen en eventueel op welke leeftijd zij daaraan zijn overleden;<br>• de bron van deze informatie is vaak de patiënt zelf;<br>• laat de velden open als de informatie niet duidelijk of onbekend is.	chez quels membres de la famille il survient, à quel âge il a commencé chez eux et éventuellement à quel âge ils en sont morts;<br>• la source de ces informations est souvent le patient lui-même<br>• laissez les champs vides si les informations ne sont pas claires ou si elles sont inconnues.																
8	REC	Bij patiënten zonder gekende geneesmiddelenallergie of -intolerantie dient dit genoteerd te zijn in het EPD.	Si le patient n'a pas d'allergies connues ou d'antécédents de réactions indésirables, cela est noté de manière appropriée dans le dossier.	18	1	3	1	0	1	5	3	1	3	39	6	23	Uncertain	Uncertain	**Discussion**
9	REC	Psychogeriatrisch onderzoek: indien het een kwetsbare betreft selecteert wordt dit genoteerd in het EPD in de afgesproken rubrieken rond kwetsbaarheid.	Examen psychogériatrique : si une personne fragile est sélectionnée, cela est noté dans le DMI dans les sections convenues concernant la fragilité.	17	1	1	0	1	1	1	6	6	0	71	7	15	Uncertain	Yes	**Discussion**
10	REC	Sociale gegevens worden vastgelegd in het dossierdeel Sociale gegevens.<br>Onder sociale gegevens worden verstaan:<br>• opleiding, beroep, werk en sociaaleconomische status;<br>• ook taal- en taalvaardigheid, gezondheidsvaardigheden, woonsituatie, gezinssituatie, aanwezigheid van mantelzorg,	Les données sociales sont enregistrées dans la section du fichier consacrée aux données sociales.<br>Les données sociales comprennent:<br>• l'éducation, la profession, l'emploi et le statut socio-économique;<br>• la langue et l'alphabétisation, les compétences en matière de	17	1	0	0	2	2	2	5	3	2	59	7	42	Uncertain	Uncertain	**Discussion**

#	Type	Description		Total	Likert score											Rank % top 5	Potential	Agreement	Conclusion
		Nl	Fr		Lowest			Middle			Highest			% Highest	Median				
					1	2	3	4	5	6	7	8	9						
		migratiehistorie, overtuigingen en heftige emotionele gebeurtenissen in het verleden vallen onder sociale gegevens	santé, la situation du logement, la situation familiale, la présence de soins informels, l'historique des migrations, les croyances et les événements émotionnels intenses survenus dans le passé sont également inclus dans les données sociales.																

Topic 5. Patiëntenidentificatie / Contactinformatie / 5. Identification du patient / Informations de contact

#	Type	Description		Total	Likert score											Rank % top 3	Potential	Agreement	Conclusion
		Nl	Fr		Lowest			Middle			Highest			% Highest	Median				
					1	2	3	4	5	6	7	8	9						
1	REC	In het EPD dient geregistreerd te zijn in welke urgentiedienst (ziekenhuis) de patiënt gekend is.	Le service des urgences (hôpital) dans lequel le patient est connu doit être enregistré dans le DMI.	17	1	5	1	1	5	2	1	1	0	12	5	14	Uncertain	Uncertain	**Discussion**
2	REC	In het EPD dient geregistreerd te zijn of de patiënt gehospitaliseerd is	Le DMI doit indiquer si le patient est hospitalisé ou non.	17	1	2	2	3	2	1	2	3	1	35	5	4	Low	No	**No selection**
3	REC	De contactgegevens van de contactpersonen, mantelzorgers en als vertegenwoordiger aangewezen personen worden vastgelegd in het dossier.	Les coordonnées des personnes de contact, des aidants informels et des personnes désignées comme représentants sont consignées dans le dossier.	18	0	0	0	0	1	1	6	8	2	89	8	65	High	Yes	**Selection**
4	REC	Het EPD dient informatie te bevatten over het zorgteam dat de patiënt opvolgt.	Le DMI doit contenir des informations sur l'équipe soignante qui suit le patient.	18	0	0	1	1	1	2	4	7	2	72	7.5	63	High	Yes	**Selection**
5	REC	Het EPD dient persoonlijke informatie te bevatten relevant voor de zorgverlener, zijnde: adres patiënt, werkgever, huis en werk telefoonnummer en burgerlijke staat.	Le DMI doit contenir des informations personnelles pertinentes pour le prestataire de soins de santé, à savoir : l'adresse du patient, son employeur, son numéro de téléphone au domicile et au travail et sa situation de famille.	18	1	0	0	1	2	1	6	5	2	72	7	50	High	Yes	**Selection**

Topic 6. Vaccinatiestatus / 6. Statut vaccinal

#	Type	Description		Total	Likert score											Rank % top 2	Potential	Agreement	Conclusion
		Nl	Fr		Lowest			Middle			Highest			% Highest	Median				
					1	2	3	4	5	6	7	8	9						
1	IND	Percentage van de populatie, welke 7 jaar of ouder zijn, die alle basisvaccinaties gekregen hebben.(Overzicht basisvaccinatieschema) Voorstel aanpassing: Basisvaccinaties opsplitsen: bv percentage patienten dat een (up-to-date) tetanus vaccinatie heeft, MBR vaccinatie,...	Pourcentage de la population âgée actuellement de 7 ans ayant reçu les vaccinations primaires recommandées pour les enfants. (Calendrier de vaccination | vaccination-info) Proposition d'adaption: diviser les vaccinations de base : par exemple, le pourcentage de patients ayant reçu une vaccination (à jour) contre le tétanos, une vaccination MBR,...	17	2	0	1	1	0	0	2	9	2	76	8	50	High	Yes	**Selection**
2	IND	Percentage van de patiëntenpopulatie, 65 jaar en ouder, dat een griepvaccinatie heeft ontvangen.	Pourcentage des patients âgés de 65 ans et plus qui ont été vaccinés contre la grippe.	18	1	0	0	0	1	0	3	8	5	89	8	47	High	Yes	**Selection**
3	REC	Het EPD dient informatie te bevatten dat de patiënt niet gevaccineerd werd.	Le DMI doit indiquer que le patient n'a pas été vacciné.	18	2	2	4	1	1	4	2	2	0	22	4.5	0	Uncertain	Uncertain	**Discussion**
4	REC	Het EPD dient per vaccinatie de datum van toediening te bevatten.	Le DMI doit contenir la date d'administration de chaque vaccin.	18	0	0	0	0	1	0	5	7	5	94	8	53	High	Yes	**Selection**

Topic 7. Wil van de patiënt / 7. Volontés du patient

#	Type	Description		Total	Likert score											Rank % top 3	Potential	Agreement	Conclusion
		Nl	Fr		Lowest			Middle			Highest			% Highest	Median				
					1	2	3	4	5	6	7	8	9						
1	REC	De verslaglegging van de gesprekken met de patiënt over wensen voor behandelingen en bij het levenseinde wordt in de eerste plaats vastgelegd in SOEP-verslagen in een episode "Gesprek levenseinde/behandelwensen" (ICPC A20, versie 2018)	Les rapports sur les conversations des patients concernant leurs souhaits en matière de traitements et de fin de vie sont principalement enregistrés dans les rapports SOEP dans un sous-contact "Conversation sur les souhaits en matière de fin de vie/traitement" (ICPC A20, version 2018).	17	2	2	2	0	1	1	3	6	0	53	7	57	Uncertain	No	**Discussion**
2	REC	De uiteindelijke besluiten over wel of niet behandelen in specifieke situaties worden vastgelegd in het dossierdeel "Behandelgrenzen". Deze behandelgrenzen zijn: registratie cardiopulmonaire resuscitatie (Reanimatie); opname in het ziekenhuis, opname op intensive care, kunstmatige beademing, toediening van een bloedproduct, overige behandeling (in vrije tekst) of toediening van antibiotica.	Les décisions finales sur l'opportunité de traiter ou non dans des situations spécifiques sont enregistrées dans la section du dossier "Limites de traitement". Ces limites de traitement sont les suivantes: enregistrement de la réanimation cardio-pulmonaire (RCP) ; hospitalisation, admission en unité de soins intensifs, ventilation artificielle, administration d'un produit sanguin, autre traitement (en texte libre) ou administration d'antibiotiques.	18	1	1	0	0	0	0	4	8	4	89	8	44	High	Yes	**Selection**
3	REC	Schriftelijke wilsverklaringen, bijvoorbeeld een euthanasieverklaring of niet-reanimeerverklaring, die de patiënt aan de huisarts aanbiedt, worden als correspondentie toegevoegd aan het EPD	Les déclarations écrites de volonté, par exemple une déclaration d'euthanasie ou de non-réanimation, proposées par le patient au médecin généraliste sont ajoutées au DMI en tant que correspondance.	18	0	2	0	0	2	2	2	7	3	67	8	33	Uncertain	Uncertain	**Discussion**
4	REC	Zijn er doelen voor persoonsgerichte zorg (Individueel Zorgplan = IZP) geregistreerd in het dossier? Bij persoonsgerichte zorg staan de	Les objectifs des soins centrés sur la personne sont-ils consignés dans le dossier ? Dans les soins centrés sur la personne,	17	1	1	2	3	0	2	0	5	3	47	6	27	Uncertain	Uncertain	**Discussion**

#	Type	Description		Total	Likert score											Rank % top 3	Potential	Agreement	Conclusion
		Nl	Fr		Lowest			Middle			Highest			% Highest	Median				
					1	2	3	4	5	6	7	8	9						
		doelen van de patiënt centraal. De patiënt bepaalt samen met zijn zorgverleners de doelen voor de zorg. Deze doelen zijn vaak niet medisch ingestoken, maar hebben er wel raakvlakken mee. Bijvoorbeeld: een COPD-patiënt wil nog dagelijks buiten een ommetje kunnen lopen en met de buren babbelen. Een goede instelling van de COPD in combinatie met loop- en ademhalingstraining kunnen het gestelde doel bereikbaar maken. Daarbij moet er afstemming zijn tussen de huisarts (instelling COPD), poh-somatiek (reguliere COPD controles) en een gespecialiseerde fysiotherapeut (loop- en ademhalingstraining). Het IZP maakt inzichtelijk wie wat doet en wie waarvoor verantwoordelijk is.	les objectifs du patient sont au centre des préoccupations. Le patient détermine les objectifs des soins avec ses prestataires de soins de santé. Ces objectifs ne sont souvent pas de nature médicale, mais présentent des similitudes. Par exemple, un patient atteint de BPCO souhaite pouvoir se promener tous les jours à l'extérieur et discuter avec ses voisins. Une adaptation adéquate de la BPCO, combinée à un entraînement à la marche et à la respiration, peut rendre l'objectif fixé réalisable. Cela nécessite une coordination entre le médecin généraliste (définition de la BPCO), les poh-somatics (contrôles réguliers de la BPCO) et un physiothérapeute spécialisé (entraînement à la marche et à la respiration). C'est indique clairement qui fait quoi et qui est responsable de quoi.																
5	REC	Het EPD dient informatie te bevatten betreffende de screeningstrajecten van toepassing voor iedere patiënt of de wensen van de patiënt betreffende deze screening. In België is aanwezig als bevolkingsonderzoek: borstkanker, baarmoederhalskanker, dikke darmkanker, aangeboren aandoeningen. (Soorten bevolkingsonderzoeken | Bevolkingsonderzoek) Opmerking: hier gaat het over accepteren/afwijzen van zorgaanpakken en niet over aanwezigheid van individuele	Le DMI doit contenir des informations sur les voies de dépistage applicables à chaque patient ou sur les souhaits du patient concernant ce dépistage. En Belgique, le dépistage de la population concerne le cancer du sein, le cancer du col de l'utérus, le cancer du côlon et les maladies congénitales (Soorten bevolkingsonderzoeken | Bevolkingsonderzoek). Remarque : nous parlons ici de l'acceptation/du rejet des plans de soins et non de la présence de plans	17	0	0	1	2	1	2	1	7	3	65	8	43	Uncertain	Uncertain	**Discussion**

#	Type	Description		Total	Likert score											Rank % top 3	Potential	Agreement	Conclusion
		Nl	Fr		Lowest			Middle			Highest			% Highest	Median				
					1	2	3	4	5	6	7	8	9						
		planningen (zoals de aanbeveling in topic 4).	individuels (comme le recommendation du topic 4).																

Topic General suggestions
