# Supplementary material for: Development of Quality Indicators for the Correct Use of Electronic Medical Records in Primary Care: Modified Delphi Study
Source: JMIR Med Inform. 2026 Jan 19;14:e80057. doi: 10.2196/80057 (PMC12865340; doi:10.2196/80057)
Supplement: Multimedia Appendix 2 [file medinform_v14i1e80057_app2.pdf]

Ontwikkeling van kwaliteitsindicatoren voor het goed gebruik van het elektronisch patiëntendossier in de huisartsgeneeskunde

Geachte heer,
Geachte mevrouw,

In de eerste plaats willen wij u bedanken voor uw bereidwilligheid deel te nemen aan ons expertenpanel. Het onderzoek waarin het expertpanel een belangrijke rol speelt, wordt uitgevoerd door het Academisch Centrum voor Huisartsgeneeskunde van de KU Leuven en heeft als doel een selectie van kwaliteitsindicatoren voor goed gebruik elektronisch medisch dossier in huisartsgeneeskunde te ontwikkelen. Een specifieke vereiste voor indicatorselectie betreft de rechtstreekse extraheerbaarheid uit het Elektronisch Medisch Dossier (EMD). Het uiteindelijke doel is om op basis van de kwaliteitsindicatoren automatische feedback te verschaffen aan de huisarts over de kwaliteit van de medische dossiers van zijn/haar patiënten. Op die manier trachten wij een verbetering te bekomen van de kwaliteit van zorg.

U bent vanwege uw kennis en expertise uitgenodigd om deel te nemen aan de selectie van kwaliteitsindicatoren voor het goed gebruik van het elektronisch patiëntendossier in de huisartsenpraktijk. Zoals in de uitnodiging reeds werd meegedeeld, maken we voor de selectie van kwaliteitsindicatoren in dit onderzoek gebruik van de Rand gemodificeerde Delphi methode. Dit is een consensusmethode die wordt onderverdeeld in 3 stappen. In de eerste stap vragen wij u een lijst van aanbevelingen te scoren naar hun belangrijkheid, bruikbaarheid en relevantie in de eerste lijn. De bedoeling is om met uw hulp een korte en krachtige set van aanbevelingen op te stellen die uiteindelijk, na de hele procedure doorlopen te hebben, zal vertaald worden naar een kernset van kwaliteitsindicatoren. Het invullen van de vragenlijst neemt ongeveer 30 minuten in beslag. Graag ontvangen wij de door u ingevulde vragenlijst vóór **30 september 2024 23u59**.

In de tweede stap worden de resultaten van de ingevulde vragenlijsten geanalyseerd. Tijdens een face to face panelgesprek trachten we tot consensus te komen over de aanvaarding, de afwijzing of de herformulering van de potentiële indicatoren. Dit gesprek, waarvan de datum later wordt meegedeeld, zal ongeveer 2 uur tijd in beslag nemen.

In de derde en laatste stap is het de bedoeling om de definitieve lijst van indicatoren schriftelijk voor te leggen aan alle experts van het panel teneinde een definitieve goedkeuring te verkrijgen. Deze stap neemt niet langer dan 15 minuten in beslag en de datum daarvan wordt eveneens later bekendgemaakt.

Bij vragen of opmerkingen kan u contact opnemen per e-mail (rico.paridaens@outlook.com).

Hoogachtend,

De projectgroep bestaande uit:

Dr. Rico Paridaens, huisarts, KU Leuven
Professor Dr. Bert Vaes, huisarts, KU Leuven
Dr. Steve Van den Bulck, huisarts, KU Leuven

Er zijn 148 vragen in deze enquête.

Algemene informatie over het onderzoek

Algemene doelstellingen onderzoek

Het onderzoek waarin het expertpanel een belangrijke rol speelt, wordt uitgevoerd door het Academisch Centrum voor Huisartsgeneeskunde van de KU Leuven en heeft als doel een selectie van kwaliteitsindicatoren voor goed gebruik elektronisch medisch dossier in huisartsgeneeskunde te ontwikkelen. Een specifieke vereiste voor indicatorselectie betreft de rechtstreekse extraheerbaarheid uit het Elektronisch Medisch Dossier (EMD). Het uiteindelijke doel is om op basis van de kwaliteitsindicatoren automatische feedback te verschaffen aan de huisarts over de kwaliteit van de medische dossiers van zijn/haar patiënten. Op die manier trachten wij een verbetering te bekomen van de kwaliteit van zorg.

Informatie over de verwerking van uw persoonsgegevens

In het kader van uw deelname aan dit onderzoek zullen persoonsgegevens over u verzameld en verwerkt worden. Deze verwerking zal gebeuren in overeenstemming met de Algemene Verordening Gegevensbescherming (AVG / GDPR). De volgende categorieën van persoonsgegevens zullen verwerkt worden tijdens dit onderzoek: naam, leeftijd, geslacht, functie, werkplaats: ziekenhuis/praktijk en afdeling voor rapportering in het consensusrapport, audio-opname tijdens het face-to-face panelgesprek voor verwerking tot het consensusrapport.

Gebruik van uw persoonsgegevens

Enkel persoonsgegevens die noodzakelijk zijn voor de doeleinden van dit onderzoek zullen verzameld en verwerkt worden. Uw gegevens zullen in het kader van dit onderzoek gepseudonimiseerd worden. Dit wil zeggen dat gegevens die u kunnen identificeren zoals naam, leeftijd, geslacht, functie, werkplaats: ziekenhuis/praktijk worden losgekoppeld van de andere onderzoeksdata en vervangen worden door een unieke, willekeurige code. Op deze manier is het niet langer onmiddellijk zichtbaar welke gegevens van welke specifieke persoon afkomstig zijn. Enkel de onderzoeker kan via de unieke code de gegevens terug linken aan een specifieke persoon. Dit zal echter enkel in uitzonderlijke gevallen gebeuren, bijvoorbeeld indien u beroep doet op uw recht op inzage of rectificatie van uw gegevens. Voor de kwaliteit van het onderzoek is het belangrijk dat wij uw naam, leeftijd, geslacht, functie, werkplaats: ziekenhuis/praktijk mogen vermelden als deelnemer van de studie in de output van dit onderzoek, zoals publicaties, zal u niet geïdentificeerd worden. De resultaten zullen echter nooit rechtstreeks verwijzen naar een persoon in de output van dit onderzoek en zal steeds vermeld worden als een algemene conclusie.

Als rechtsgrond voor de verwerking van uw gegevens wordt het algemeen belang aangewend. Dit betekent dat het onderzoek zal leiden tot een vermeerdering van kennis en inzicht die de maatschappij (direct of indirect) ten goede komt. Stopzetting van deelname aan de studie houdt in dat de eerder verzamelde gegevens nog verder rechtsgeldig kunnen worden betrokken in de studie en niet moeten worden verwijderd door KU Leuven.

Uw gegevens zullen door de onderzoekers gedurende 10 jaar na afloop van het onderzoek bewaard worden op een beveiligde opslaglocatie van KU Leuven.

Uw rechten

U hebt het steeds recht om meer informatie te vragen over het gebruik van uw gegevens. Daarnaast kan u beroep doen op het recht van inzage en het recht op verbetering (rectificatie) van uw gegevens voor zover deze rechten de doeleinden van het onderzoek niet onmogelijk maken of ernstig belemmeren.

Indien u op één van deze rechten beroep wil doen, kan u contact opnemen met de onderzoekers aan de hand van de contactgegevens bovenaan deze brief.

Hergebruik van uw gegevens

De mogelijkheid bestaat dat uw gepseudonimiseerde gegevens kunnen hergebruikt worden voor wetenschappelijk onderzoek door:

- onderzoekers en/of academische partners die samenwerken met of gegevens ontvangen van KU Leuven (met inbegrip - onder bepaalde voorwaarden- van een master- of doctoraatstudent),

Het kunnen ter beschikking stellen van gegevens is essentieel om onderzoeksresultaten te kunnen valideren en de vooruitgang van de wetenschappelijke kennis te kunnen bewerkstelligen.

Ingeval van hergebruik van uw gegevens zullen steeds de nodige contractuele afspraken gemaakt worden om de persoonsgegevens te beschermen en de verantwoordelijkheden en aansprakelijkheden van de partijen te bepalen in overeenstemming met de relevante wetgeving.

In het kader van hergebruik kunnen uw persoonsgegeven worden overgedragen buiten de Europese Economische Ruimte, op voorwaarde dat passende maatregelen genomen zijn om uw persoonsgegevens te beschermen in overeenstemming met de relevante wetgeving. Met name zullen deze gegevens in principe enkel in gepseudonimiseerde vorm worden doorgegeven, en zullen alle partijen betrokken bij het onderzoek verplicht zijn om de vertrouwelijkheid van de persoonsgegevens te respecteren.

Er zal transparant geïnformeerd worden over eventueel hergebruik van uw data. Dit zal gebeuren via email.

Elk hergebruik zal steeds gebeuren conform de geldende wetgeving en conform het beleid van KU Leuven ter zake. Dit beleid houdt onder meer in dat een onafhankelijk orgaan zal waken over de bescherming van persoonsgegevens en uw rechten.

Merk op dat uw gepseudonimiseerde gegevens ook kunnen beschikbaar worden gemaakt op bepaalde dataplatformen voor verder wetenschappelijk onderzoek en dit met strikt beveiligde en gecontroleerde toegang. Dit kan bijvoorbeeld gebeuren in het kader van publicaties over resultaten. Het dataplatform voorziet een toegangsbeleid en verbindt zich ertoe toegang tot gegevens conform de geldende wetgeving te regelen.

Contactgegevens

KU Leuven fungeert als verwerkingsverantwoordelijke in het kader van dit onderzoek. Meer specifiek zal enkel de onderzoekers Rico Paridaens, Bert Vaes en Steve Van den Bulck toegang hebben tot uw persoonsgegevens. Ingeval van specifieke vragen over dit onderzoek, inclusief de verwerking van uw persoonsgegevens, kan u met hen contact opnemen.

Voor verdere vragen en bedenkingen over de verwerking van uw persoonsgegevens kan u contact opnemen met de functionaris voor gegevensbescherming voor wetenschappelijk onderzoek van KU Leuven (dpo@kuleuven.be). Gelieve hierbij te verduidelijken om welk onderzoek het gaat door vermelding van de titel en de namen van de onderzoekers.

Indien u, na contact te hebben opgenomen met de functionaris voor gegevensbescherming, een klacht zou willen indienen over hoe uw informatie wordt behandeld, kan u terecht bij de Belgische Gegevensbeschermingsautoriteit (www.gegevensbeschermingsautoriteit.be).

Specifieke informatie over het onderzoek

Van aanbeveling tot indicator

Voor het opstellen van de lijst van aanbevelingen in dit document, maakten we gebruik van nationale en internationale richtlijnen (cfr. bronvermelding). We zijn gestart met het opstellen van een lijst die alle aanbevelingen voor het goed gebruik van het elektronisch medisch dossier in de huisartsgeneeskunde uit alle geraadpleegde bronnen bevatte. Het zou echter onbegonnen werk zijn om met zulk een uitgebreide lijst aan de slag te gaan voor de selectie van relevante indicatoren. Daarom hebben we in een volgende stap die aanbevelingen geselecteerd, die bruikbaar waren in de eerste lijn en die mogelijks extraheerbaar waren uit het EMD. Dit zijn namelijk twee belangrijke voorwaarden in het kader van onze Studie. We hebben daarnaast ook gekeken of de aanbevelingen “SMART” waren opgesteld, dat wil zeggen **S**pecific (specifiek), **M**easurable (meetbaar), **A**chievable (haalbaar), **R**elevant en **T**ime-bound (tijdsgebonden). Het resultaat is een basisset van aanbevelingen zoals weergegeven in dit document.

In deze fase is het de bedoeling dat u alle aanbevelingen scoort in die mate waarin u de specifieke aanbevelingen van belang acht en extraheerbaar uit het EMD om de kwaliteit van het goed gebruik van het elektronisch patiëntendossier in de huisartsgeneeskunde te meten. Voor een verdere toelichting, zie de invulinstructies hieronder.

Invulinstructies

In de eerste plaats verzoeken wij u het in bijlage gevoegde attest ter geinformeerde toestemming in te vullen. Vervolgens vindt u in deze vragenlijst 50 indicatoren en aanbevelingen, ingedeeld in volgende categorieën:

1. Volledigheid en adequaatheid probleemlijst (16 indicatoren/aanbevelingen);
2. Gestructureerd registreren in EMD (5 indicatoren/aanbevelingen);
3. Compleetheid en actualiteit medicatie-overzicht (5 indicatoren/aanbevelingen);
4. Risicofactoren / Medicatiebewaking (10 indicatoren/aanbevelingen);
5. Patiëntenidentificatie / Contactinformatie (5 indicatoren/aanbevelingen);
6. Vaccinatiestatus (4 indicatoren/aanbevelingen); en
7. Wil van de patiënt (5 indicatoren/aanbevelingen).

Wij vragen van u om iedere aanbeveling te beoordelen op de mate waarin deze aanbeveling extraheerbaar is uit het EMD en van belang is om de kwaliteit van het elektronisch medisch dossier in de huisartsgeneeskunde te meten met behulp van een 9-punten Likert schaal. Dit is een schaal van 1 tot en met 9, met als laagste score een 1 (slechte maat voor het meten van de kwaliteit) en als hoogste score een 9 (uitstekende maat voor het meten van de kwaliteit).

Om te beoordelen of een aanbeveling in aanmerking komt voor het meten van de kwaliteit van zorg, kunt u zich baseren op de volgende criteria:

- De aanbeveling is relevant in het zorgproces in de eerste lijn.
- De aanbeveling verbeterd de gezondheid van de patiënt.
- De aanbeveling verbetert de kwaliteit van leven van de patiënt.
- De aanbeveling verbetert de doelmatigheid van zorg voor de patiënt.
- De aanbeveling kan vertaald worden naar een indicator die automatisch extraheerbaar is uit het EMD.

Naast een score volgens de 9-punten Likert schaal, vragen wij u ook om, na het beoordelen van alle aanbevelingen uit één categorie, per categorie een top 5 van aanbevelingen op te stellen op basis van hun geschiktheid voor het meten van de kwaliteit van zorg. Per categorie bieden wij u de mogelijkheid aanvullingen te noteren aan deze top 5.

We bieden de mogelijkheid aan om zelf opmerkingen te formuleren, aanbevelingen verder aan te vullen of zelf aanbevelingen neer te schrijven op het einde van de vragenlijst.

Gebruikte bronnen

Hieronder worden de bronnen opgelijst die geraadpleegd werden voor het opstellen van de lijst met aanbevelingen. Tevens vermeld zijn de afkortingen zoals die gebruikt worden in de vragenlijst (voor de bron), het jaar van publicatie of laatste update en het land van herkomst.

- Domus Medica: Verdonck P, Strobbe J, Steenackers J et al. Het elektronisch medisch dossier. Huisarts Nu maart 2004; 33(2).
- SSMG: Société Scientifique de Médecine Générale (SSMG). Organisation de la pratique. [cited 2024 Jan 27]; Available from: https://www.ssmg.be/organisation-pratique/
- NCQA: National Committee for Quality Assurance (NCQA). Guidelines for Medical Record Documentation. 2018 [cited 2024 Jan 27]; Available from: https://www.ncqa.org/wp-content/uploads/2018/07/20180110_Guidelines_Medical_Record_Documentation.pdf
- ADEPD: Duineveld B, Kole HM, Van Werven H. NHG-Richtlijn Adequate dossiervorming met het elektronisch patiëntdossier (ADEPD). 2019 [cited 2024 Jan 27]; Available from: https://www.nhg.org/praktijkvoering/informatisering/richtlijn-adequate-

dossiervorming-epd/

- HASP: Federatie Medisch Specialisten (FMS), Nederlands Huisartsen Genootschap (NHG). Richtlijn Informatie-uitwisseling tussen huisarts en medisch specialist (HASP. 2017 [cited 2024 Jan 27]; Available from: https://www.nhg.org/praktijkvoering/gegevensuitwisseling/gegevensuitwisseling-huisarts-specialist-hasp/
- NHS: National Health Service (NHS). Summary Care Records. Version 1.3, 19 April 2023. [cited 2024 Jan 27]; Available from: https://www.england.nhs.uk/long-read/summary-care-records-scr/
- HIQA: Health Information and Quality Authority. Recommendations on the implementation of a national electronic patient summary in Ireland. 2020 [cited 2024 Jan 27]; Available from: https://www.hiqa.ie/reports-and-publications/health-information/recommendations-implementation-national-electronic
- Hiddema-van der Wal: Hiddema-van der Wal A, van der Werf GTh, Meyboom-de Jong B. Welke ICPC-codes willen huisartsen automatisch laten toevoegen aan de probleemlijst? Huisarts en Wetenschap 46(10) september 2003. Page 539-543
- EPD-scan-h: Lea Jabaaij, Robert Verheij, Khing Njoo, Henk van den Hoogen, Waling Tiersma, Herman Levelink. Het meten van de kwaliteit van de registratie in elektronische patiënten dossiers van huisartsen met de EPD-scan-h (EPD-scan-h). ISBN 978-90-6905-896-2. 2008
- Hamade et al: Hamade N, Terry A, Malvankar-Mehta M. Interventions to improve the use of EMRs in primary health care: a systematic review and meta-analysis. BMJ Health Care Inform. 2019 May;26(1):e000023.
- De Lusignan et al: De Lusignan S. Does Feedback Improve the Quality of Computerized Medical Records in Primary Care? Journal of the American Medical Informatics Association. 2002 Jul 1;9(4):395–401.
- CIHI: Ottawa - Ontario : Canadian Institute for Health Information. Pan-Canadian primary health care indicator - update report. 2016. Canada. ISBN: 978-1-77109-146-6

Informed Consent

Titel van het onderzoek:
Ontwikkeling van kwaliteitsindicatoren voor het goed gebruik van het elektronisch patiëntendossier in de huisartsgeneeskunde

Naam + contactgegevens promotor en onderzoeker(s):

- **Onderzoeker: Rico Paridaens, rico.paridaens@outlook.com, 0470 83 39 09**
- **Promotor: Bert Vaes, bert.vaes@kuleuven.be, 0474 33 05 13, Departement Maatschappelijke Gezondheidszorg en Eerstelijnszorg, Academisch Centrum voor Huisartsgeneeskunde**

Doel en methodologie van het onderzoek:
INTEGO poogt om kwaliteitsindicatoren op te stellen voor de belangrijkste ziektebeelden in de huisartsgeneeskunde en algemeen het gebruik van het EPD door huisartsen door middel van RAND-Modiphied Delphi procedures. Dit is een procedure waarbij elke deelnemer systematische enkele stappen doorloopt om uiteindelijk een consensusrapport met aanbevelingen te bekomen welke gedragen wordt door alle deelnemers. Voor dit onderzoek zullen wij kwaliteitsindicatoren bepalen voor:

Tijdens het onderzoek doorloopt u volgende stappen:

1. **Online vragenronde: U wordt gevraagd een lijst van potentiële kwaliteitsindicatoren / aanbevelingen te scoren op hun capaciteit om de kwaliteit van het onderwerp te meten aan de hand van een Likert schaal van 1 (= laagste score) tot 9 (= hoogste score). U ontvangt een feedbackrapport over de eerste ronde met uw persoonlijke score, de mediane score van alle deelnemers en op basis hiervan het potentieel van de kwaliteitsindicator/aanbeveling.**
2. **Face-to-face panelgesprek: De matige aanbevelingen (twijfelachtig resultaat) worden bediscussieerd alsook de nieuw aangebrachte indicatoren. De sterke en zwakke indicatoren komen enkel ter sprake indien hier opmerkingen over geformuleerd zijn. Op basis van de conclusies van de face-to-face samenkomst wordt een set van aanbevelingen opgesteld welke voor een derde en laatste beoordeling wordt voorgelegd aan alle panelleden ter goedkeuring.**

Duur van het experiment: +/- 30 min

Ik begrijp en stem toe met:

- **Ik begrijp wat van mij verwacht wordt tijdens dit onderzoek.**
- **Ik weet dat ik zal deelnemen aan volgende proeven of testen: Online bevraging en face-to-face panelgesprek**
- **Ik weet dat er risico's of ongemakken kunnen verbonden zijn aan mijn deelname: Mogelijke conflictsituaties tijdens de discussies van het face-to-face panelgesprek**
- **Ikzelf of anderen kunnen baat bij dit onderzoek hebben op volgende wijze: Deze kwaliteitsindicatoren kunnen gebruikt**

worden tot het verschaffen van feedback aan huisartsen in België en andere landen omtrent de kwaliteit van de zorg voor patiënten.

- **Mijn deelname levert een bijdrage aan het wetenschappelijk onderzoek. Ik weet ik geen verdere beloning of compensatie voor mijn deelname zal ontvangen.**
- **Ik begrijp dat mijn deelname aan deze studie vrijwillig is. Ik heb het recht om mijn deelname op elk moment stop te zetten. Daarvoor hoef ik geen reden te geven en ik weet dat daaruit geen nadeel voor mij kan ontstaan.**
- **Ik weet dat er in dit onderzoek opnames van mij gemaakt kunnen worden: opname van audio tijdens het face-to-face panelgesprek voor ontwikkeling van het consensusrapport.**
- **Mijn persoonsgegevens zullen verwerkt worden in lijn met de Algemene Verordening Gegevensbescherming (AVG/GDPR). Hierbij worden alleen de gegevens verwerkt die strikt noodzakelijk voor het behalen van de onderzoeksdoelstellingen. Doorheen het onderzoek zullen mijn gegevens steeds vertrouwelijk behandeld worden. De onderzoekers nemen maatregelen om mijn privacy te beschermen. Zo zullen mijn persoonsgegevens gepseudonimiseerd worden, wat betekent dat mijn gegevens niet meer aan mij gekoppeld kunnen worden zonder gebruik van aanvullende informatie die alleen toegankelijk is voor de onderzoekers. Ik begrijp dat mijn gepseudonimiseerde gegevens kunnen hergebruikt worden voor ander wetenschappelijk onderzoek en mogelijk ook in het kader van onderwijs en academische lezingen. Meer informatie over de verwerking van mijn persoonsgegevens kan teruggevonden worden in de bijgevoegde informatiebrief.**

Ik wil graag op de hoogte gehouden worden van de resultaten van dit onderzoek. De onderzoeker mag mij hiervoor contacteren op het volgende e-mailadres:

Vul uw antwoord hier in:

[ ]

- **Hoewel dit onderzoek geen diagnostische screening inhoudt, bestaat er een kleine kans dat de onderzoekers toevallig zouden stuiten op onvoorziene onderzoeksresultaten waarvan zij het nodig achten mij op de hoogte te stellen (bv. mogelijke aanwijzingen van medische of psychische problemen). In dat geval mogen ze contact opnemen via het bovenstaande e-mailadres om me hiervan op de hoogte te brengen. Indien ik niet wens om hiervan op de hoogte te worden gebracht, kruis ik het onderstaande vakje aan.**

Kies alle voor u geldende mogelijkheden:

☐ Ik wens niet op de hoogte te worden gebracht van onvoorziene onderzoekbevindingen.

- **Voor verdere vragen over het onderzoek weet ik dat ik na mijn deelname terecht kan bij:**
  Rico Paridaens (rico.paridaens@outlook.com)
- **Deze studie werd beoordeeld en goedgekeurd door de Sociaal-Maatschappelijke Ethische Commissie (SMEC) van KU Leuven (G-2024-8020, gelieve dit nummer te vermelden in elke communicatie over het onderzoek). Voor eventuele klachten of andere bezorgdheden omtrent ethische aspecten van deze studie kan ik contact opnemen met SMEC: smec@kuleuven.be**
- **Ik weet dat ik bij onderstaande terecht kan indien ik na het onderzoek ongemakken of moeilijkheden ervaar als gevolg van de thema's die in het onderzoek aan bod kwamen:**
  Rico Paridaens (rico.paridaens@outlook.com)

Ik heb bovenstaande informatie gelezen en begrepen en heb antwoord gekregen op al mijn vragen betreffende deze studie. Ik stem toe om deel te nemen.

*

Kies één van de volgende mogelijkheden:

◯ Akkoord

◯ Niet akkoord

Persoonlijke info

Naam en voornaam:
(Deze info wordt enkel gebruikt voor het verzenden van de resultaten van de eerste Delphi ronde en voor de uitnodiging voor het panelgesprek.) *

Vul uw antwoord hier in:

E-mail adres:
(Deze info wordt enkel gebruikt voor het verzenden van de resultaten van de eerste Delphi ronde en voor de uitnodiging voor het panelgesprek.) *

Vul uw antwoord hier in:

Leeftijd: *

Vul uw antwoord hier in:

Geslacht: *

Indien u 'Anders:' kiest, licht deze keuze dan toe in het bijbehorende tekstvak.
Kies één van de volgende mogelijkheden:

- Man
- Vrouw
- Overige

Werkplaats:
(Zo ziekenhuis: specifieer)
*

Kies één van de volgende mogelijkheden:

- ◯ Ziekenhuis
- ◯ Huisartsenpraktijk: solo-/duopraktijk
- ◯ Huisartsenpraktijk: groepspraktijk

Geef hier een toelichting op uw antwoord:

Functie bij werkplaats: *

Kies één van de volgende mogelijkheden:

- ◯ Huisarts
- ◯ Overige

Affiliatie: *

Kies één van de volgende mogelijkheden:

- ◯ Universiteit Gent
- ◯ Universiteit Hasselt
- ◯ KU Leuven
- ◯ Universiteit Antwerpen
- ◯ Vrije Universiteit Brussel
- ◯ Université Liège
- ◯ Université catholique de Louvain
- ◯ Université Saint-Louis - Bruxelles
- ◯ Université Namur
- ◯ Université Mons
- ◯ Université libre de Bruxelles
- ◯ Overige

Welke EMD gebruikt u? *

Kies één van de volgende mogelijkheden:

- ◯ CareConnect
- ◯ HealthOne
- ◯ Medispring
- ◯ Daktari
- ◯ Overige

1. Volledigheid en adequaatheid probleemlijst

In welke mate zijn de volgende aanbevelingen relevant voor het meten van de kwaliteit van het goed gebruik van het EMD in de huisartsgeneeskunde in verband met de **volledigheid en adequaatheid van de probleemlijst** van patiënten?

Indicator / Aanbeveling	Bron	Jaar	Evidentiegraad
**INDICATOR:**<br>**Hoeveel actieve zorgelementen heeft een patiënt gemiddeld op zijn/haar lijst met zorgelementen?**	**EPD-scan-h**	**2009**	**Geen gradatie**
**GEKOPPELDE AANBEVELING:**<br>**Episodelijst: het EPD presenteert achtereenvolgens open episodes met vlag, afgesloten episodes met vlag, open episodes zonder vlag.**	**ADEPD, HIQA**	**2019, 2020**	**Geeb gradatie**

Uw oordeel:

*

Kies één van de volgende mogelijkheden:

- ◯ 1 (Slecht)
- ◯ 2
- ◯ 3
- ◯ 4
- ◯ 5
- ◯ 6
- ◯ 7
- ◯ 8
- ◯ 9 (Uitstekend)
- ◯ Niet beoordeelbaar

Beoordeling gebaseerd op:

Kies alle voor u geldende mogelijkheden:

- ☐ EMD extraheerbaarheid
- ☐ Relevantie aanbeveling

Indicator / Aanbeveling	Bron	Jaar	Evidentiegraad
**INDICATOR:**<br>**Hoeveel procent van de probleemstatuswaardige episodes heeft inderdaad het label 'bijzondere attentiewaarde'?**	**EPD-scan-h**	**2009**	**Geen gradatie**

Bijkomende info: Problemen met een bijzondere attentiewaarde worden automatisch bovenaan de probleemlijst geplaatst opdat deze goed zichtbaar zouden zijn in het EPD. Probleemstatuswaardige zorgelementen zijn zorgelementen met een ICPC-code die in aanmerking komen voor automatische plaatsing op de probleemlijst omdat ze belangrijk zijn voor de zorgverlener. Deze zorgelementen zijn:

A12	allergie/allergische reactie neg.	N88	epilepsie alle vormen
A70	tuberculose gegen. [excl. R70]	N89	migraine
A79	maligne neopl. (onbekende prim.lkk)	P15	chronisch alcoholmisbruik
A85	geneesmiddelbijwerking	P18	geneesmiddelmisbruik
A90	congen. syndromen/mult. afwijkingen	P21	overactief kind/hyperkin. syndroom
B72	Hodgkin/andere maligne lymfomen	P70	dementie (incl. seniel/Alzheimer)
B73	leukemie	P71	andere organische psychosen
B74	andere mal. neopl. bloed/bloedv. org.	P72	schizofrenie alle vormen
B76	miltruptuur	P73	affectieve psychosen
B78	erfelijke hemolytische anemie	P74	angsttoestand/ziekelijke angst
B79	and. aangeb. afw. bloed/bloedv. org	P76	depressie
B81	pernicieuze/foliumzuurdefic. anemie	P77	suïcidepoging

B83	purpura/stoll.st./afw. thromb.	R70	tuberculose tr. resp. [exc gegen.A70]
D74	maligne neoplasma maag	R78	acute bronchitis (>2x/ja
D75	maligne neoplasma colon/rectum	R82	pleuritis alle vormen [exc tbc R70]
D76	maligne neoplasma pancreas	R84	maligne neoplasma bronchus/long
D77	mal. neopl. tr. dig. and./niet gespec.	R85	ander maligne neoplasma resp.
D78	benigne tumoren maag-darmkanaal	R89	aangeboren afwijkingen t resp.
D81	aangeboren afwijking tr. digestivus	R91	chron. bronchitis/bronchiëctasie
D85	ulcus duodeni	R95	emfyseem/COPD
D86	andere peptische ulcera	R96	astma
D92	diverticulose/diverticulitis	R97	hooikoorts/allergische rhinitis
D93	spastisch colon/IBS	S77	maligne neoplasma huid/subcutis
D94	colitis ulcerosa/chron. enteritis	S87	constitutioneel eczeem
D97	cirrose/andere leverziekten neg.	S91	psoriasis (incl. artropathi
D98	cholecystitis/cholelithiasis	T71	maligne neoplasma schildklier
F81	andere aangeboren afwijkingen oog	T72	benigne neoplasma schildklier
F92	cataract	T80	and. aangeb. afw. endo. kl./metab.
F93	glaucoom	T81	struma/noduli schildklier [excl. T85]
F94	blind/visus vermind. (alle vormen)	T85	hyperthyreoïdie (met/zo struma)
H83	otosclerose	T86	hypothyreoïdie/myxoede
H84	presbyacusis	T90	diabetes mellitus

K73	aangeboren afw. tr. circulatorius	T92	jicht
K74	angina pectoris	T93	vetstofwisselingsstoornis
K75	acuut myocardinfarct	U71	urineweginfecties (>2x/jaar)
K76	and. chron. ischemische hartziekten	U75	maligne neoplasma nier
K77	decompensatio cordis	U76	maligne neoplasma blaas
K78	boezemfibrilleren/-fladderen	U77	andere maligne neopl. urinewegen
K79	paroxismale tachycardie	U85	aangeboren afwijkingen urinewegen
K83	klepaandoening niet-reumat./nao	U88	glomerulonefritis/nefros[illegible]
K86	hypertensie zond. orgaanbeschadig.	U95	urolithiasis (alle vormen/lok.)
K87	hypertensie met orgaanbeschadig.	W13	sterilisatie vrouw/verwijzing
K89	passagère cerebrale ischemie/TIA	W72	maligne neopl. i.v.m. zwangerschap
K90	cerebrovasc. accid. (CVA) [excl. TIA]	W76	aangeb. afw. moeder als zwang.compl.
K91	atherosclerose [excl. coron./cerebr.]	W80	ectopische zwangerschap
K92	andere ziekten perifere arteriën	W82	spontane abortus/abortu[illegible] nao
K93	longembolie/longinfarct	W92	gecompl. partus levendgeborene
K94	tromboflebitis/flebotrombose	W93	gecompl. partus doodgeborene
L75	fractuur femur	X74	ontstekingen kleine bekken/PID
L82	aangeboren afw. bewegingsapparaat	X75	maligne neoplasma cervix uteri
L88	reumatoïde artritis/verwante aand.	X76	maligne neopl. borsten (vrouw)

**L89**	**coxartrose**	**X77**	**and. maligne neopl. gesl.[illegible] (vrouw)**
**L95**	**osteoporose**	**X78**	**benigne neopl. uterus/cervix uteri**
**N70**	**poliomyelitis/and. enterovirusinf.**	**X83**	**aangeboren afw. gesl.org[illegible] (vrouw)**
**N71**	**meningitis/encefalitis**	**X87**	**prolaps vagina/uterus**
**N72**	**tetanus**	**Y77**	**maligne neoplasmata prostaat**
**N74**	**maligne neoplasma zenuwstelsel**	**Y78**	**and. mal. neopl. gesl.org/borst. (man)**
**N75**	**benigne neoplasma zenuwstelsel**	**Y82**	**hypospadie**
**N85**	**aangeboren afwijkingen zenuwst.**	**Y83**	**cryptorchisme/niet inged[illegible] testis**
**N86**	**multiple sclerose**	**Y84**	**and. aangeb. afw. gesl.or[illegible] (man)**
**N87**	**parkinsonisme/ziekte v. Parkinson**	**Y85**	**benigne prostaathypertro[illegible]**

Bron: A Hiddema-van der Wal, GTh van der Werf, B Meyboom-de Jong, "Welke ICPC-codes willen huisartsen automatisch laten toevoegen aan de probleemlijst?

Uw oordeel:

*****

Kies één van de volgende mogelijkheden:

- ◯ 1 (Slecht)
- ◯ 2
- ◯ 3
- ◯ 4
- ◯ 5
- ◯ 6
- ◯ 7
- ◯ 8
- ◯ 9 (Uitstekend)
- ◯ Niet beoordeelbaar

Beoordeling gebaseerd op:

Kies alle voor u geldende mogelijkheden:

- [ ] EMD extraheerbaarheid
- [ ] Relevantie aanbeveling

Indicator / aanbeveling	Bron	Jaar	Evidentiegraad
**INDICATOR:**<br>**Een aantal medicamenten worden voorgeschreven specifiek in het kader van een bepaalde ziekte. Voor patiënten met een voorschrift voor deze medicatie dient een zorgelement met die diagnose aanwezig te zijn. Afwezigheid hiervan kan wijzen op gebrek van registratie van dit zorgelement in het EPD. Deze aandoeningen zijn:**<br>• **Schildklierlijden**<br>• **Epilepsie**<br>• **Ziekte van Parkinson**<br>• **Depressie**<br>• **Cardiovasculaire ziekte**<br>• **Astma/COPD**<br>• **Diabetes**	**EPD-scan-h**	**2009**	**Geen gradatie**

Uw oordeel:

*

Kies één van de volgende mogelijkheden:

- ○ 1 (Slecht)
- ○ 2
- ○ 3
- ○ 4
- ○ 5
- ○ 6
- ○ 7
- ○ 8
- ○ 9 (Uitstekend)
- ○ Niet beoordeelbaar

Beoordeling gebaseerd op:

Kies alle voor u geldende mogelijkheden:

- [ ] EMD extraheerbaarheid
- [ ] Relevantie aanbeveling

Indicator / aanbeveling	Bron	Jaar	Evidentiegraad
**INDICATOR:** **Percentage van geregisteerde patiënten waarvoor er geen aanpassing was in het EPD over de voorbije 12 maanden.**	**De Lusignan et al**	**2002**	**Geen gradatie**

Uw oordeel:

*

Kies één van de volgende mogelijkheden:

- ◯ 1 (Slecht)
- ◯ 2
- ◯ 3
- ◯ 4
- ◯ 5
- ◯ 6
- ◯ 7
- ◯ 8
- ◯ 9 (Uitstekend)
- ◯ Niet beoordeelbaar

Beoordeling gebaseerd op:

Kies alle voor u geldende mogelijkheden:

- [ ] EMD extraheerbaarheid
- [ ] Relevantie aanbeveling

Indicator / aanbeveling	Bron	Jaar	Evidentiegra
**INDICATOR:**<br>**Percentage van de patiëntenpopulatie, 18 jaar en ouder, met chronische gezondheidsproblemen die ten minste een van de volgende soorten zelfmanagementondersteuning hebben ontvangen van hun zorgverlener in de eerstelijnsgezondheidszorg: Voorzien van een behandelplan ; Aangemoedigd om zelfhulpgroepen of -programma's te gebruiken**	**CIHI**	**2016**	**Geen gradati**

Uw oordeel:

*

Kies één van de volgende mogelijkheden:

- ◯ 1 (Slecht)
- ◯ 2
- ◯ 3
- ◯ 4
- ◯ 5
- ◯ 6
- ◯ 7
- ◯ 8
- ◯ 9 (Uitstekend)
- ◯ Niet beoordeelbaar

Beoordeling gebaseerd op:

Kies alle voor u geldende mogelijkheden:

- ☐ EMD extraheerbaarheid
- ☐ Relevantie aanbeveling

Indicator / aanbeveling	Bron	Jaar	Evidentiegraad
**INDICATOR:**<br>**Hoeveel procent van de episodes op de episodelijst heeft een correcte ICPC-code?**<br>**ICPC code verder te verdelen in :**<br>• **Correct gebruik: Klachten in de range 01 tot en met 29 en diagnoses in de range 70 tot en met 99. In deze categorie zijn ook opgenomen A44 (inenting), R44 (influenzavaccinatie) en X37 (cervix-uitstrijkje bevolkingsonderzoek)**<br>• **Mogelijk incorrect gebruik: A97 (geen ziekte) of A99 (andere gegeneraliseerde of niet gespecificeerde ziekte) (als vluchtcode);**<br>• **Niet-toegestane of geen ICPC: Geen ICPC of een niet toegestane code (range 30-69), met uitzondering van A44 (preventieve inenting of medicatie), R44 (influenzavaccinatie) en X37 (cervixuitstrijkje bevolkingsonderzoek).**	**EPD-scan-h**	**2009**	**/**

Uw oordeel:

*

Kies één van de volgende mogelijkheden:

◯ 1 (Slecht)

- ◯ 2
- ◯ 3
- ◯ 4
- ◯ 5
- ◯ 6
- ◯ 7
- ◯ 8
- ◯ 9 (Uitstekend)
- ◯ Niet beoordeelbaar

Beoordeling gebaseerd op:

Kies alle voor u geldende mogelijkheden:

- ☐ EMD extraheerbaarheid
- ☐ Relevantie aanbeveling

Indicator / aanbeveling	Bron	Jaar	Evidentiegraad
**AANBEVELING:**<br>**Het is aangewezen dat communicatiebeperkingen geregistreerd staan in het EPD opdat de zorgverlener hiermee rekening kan houden.**<br>**ICPC-2 codes:**<br>• **F94 blind/visus vermind. (alle vormen)**<br>• **H84 Presbyacusis**<br>• **H86 Deafness**<br>• **In range 28 (Limited function/disability)**	**NHS**	**2023**	**Geen gradatie**

Uw oordeel:

*

Kies één van de volgende mogelijkheden:

- ◯ 1 (Slecht)
- ◯ 2
- ◯ 3
- ◯ 4
- ◯ 5
- ◯ 6
- ◯ 7
- ◯ 8
- ◯ 9 (Uitstekend)
- ◯ Niet beoordeelbaar

Beoordeling gebaseerd op:

Kies alle voor u geldende mogelijkheden:

- ☐ EMD extraheerbaarheid
- ☐ Relevantie aanbeveling

Indicator / aanbeveling	Bron	Jaar	Evidentiegraad
**AANBEVELING:** **In het EPD wordt bijkomende informatie betreffende de diagnose best vastgelegd in bijschrift of commentaar welke meer info geeft over de aspecten van de aandoening.**	**HIQA**	**2018**	**Geen evidentie**

Uw oordeel:

*

Kies één van de volgende mogelijkheden:

- ◯ 1 (Slecht)
- ◯ 2
- ◯ 3
- ◯ 4
- ◯ 5
- ◯ 6
- ◯ 7
- ◯ 8
- ◯ 9 (Uitstekend)
- ◯ Niet beoordeelbaar

Beoordeling gebaseerd op:

Kies alle voor u geldende mogelijkheden:

- ☐ EMD extraheerbaarheid
- ☐ Relevantie aanbeveling

Indicator / aanbeveling	Bron	Jaar	Evidentiegraad
**AANBEVELING:** **In het EPD dient de startdatum vastgelegd te worden van ieder zorgelement.**	**HIQA**	**2018**	**Geen gradatie**

Uw oordeel:

*

Kies één van de volgende mogelijkheden:

- ◯ 1 (Slecht)
- ◯ 2
- ◯ 3
- ◯ 4
- ◯ 5
- ◯ 6
- ◯ 7
- ◯ 8
- ◯ 9 (Uitstekend)
- ◯ Niet beoordeelbaar

Beoordeling gebaseerd op:

Kies alle voor u geldende mogelijkheden:

- ☐ EMD extraheerbaarheid
- ☐ Relevantie aanbeveling

Indicator / aanbeveling	Bron	Jaar	Evidentiegraad
**AANBEVELING:** **In het EPD dient de einddatum of vermoedelijke einddatum vastgelegd te worden van ieder afgesloten zorgelement.**	**HIQA**	**2018**	**Geen gradatie**

Uw oordeel:

*

Kies één van de volgende mogelijkheden:

- ◯ 1 (Slecht)
- ◯ 2
- ◯ 3
- ◯ 4
- ◯ 5
- ◯ 6
- ◯ 7
- ◯ 8
- ◯ 9 (Uitstekend)
- ◯ Niet beoordeelbaar

Beoordeling gebaseerd op:

Kies alle voor u geldende mogelijkheden:

- ☐ EMD extraheerbaarheid
- ☐ Relevantie aanbeveling

Indicator / Aanbeveling	Bron	Jaar	Evidentiegraad
**AANBEVELING:** **In het EPD wordt dient geregistreerd te worden dat de patiënt geen gekende ziektes heeft indien de patiënt geen gekende ziektes heeft. Op deze manier weten andere zorgverleners dat het dossier volledig is. (ICPC-2: A97)**	**HIQA**	**2018**	**/**

Uw oordeel:

*

Kies één van de volgende mogelijkheden:

- ◯ 1 (Slecht)
- ◯ 2
- ◯ 3
- ◯ 4
- ◯ 5
- ◯ 6
- ◯ 7
- ◯ 8
- ◯ 9 (Uitstekend)
- ◯ Niet beoordeelbaar

Beoordeling gebaseerd op:

Kies alle voor u geldende mogelijkheden:

- ☐ EMD extraheerbaarheid
- ☐ Relevantie aanbeveling

Aanbeveling	Bron	Jaar	Evidentiegraad
**AANBEVELING:**<br><br>**Het EPD bevat: alle aandoeningen of gegevens die van belang zijn voor de verdere hulpverlening, zoals bepaalde ingrepen en aandoeningen die kunnen recidiveren.**<br><br>**Verduidelijking: het gaat hier over operaties en belangrijke behandelingen zoals omschreven in NHG Ingrepenviewer**	**ADEPD, Domus Medica, HIQA, NCQA**	**2019, 2004, 2020, 2018**	**Geen gradatie**

Uw oordeel:

*

Kies één van de volgende mogelijkheden:

- ◯ 1 (Slecht)
- ◯ 2
- ◯ 3
- ◯ 4
- ◯ 5
- ◯ 6
- ◯ 7
- ◯ 8
- ◯ 9 (Uitstekend)
- ◯ Niet beoordeelbaar

Beoordeling gebaseerd op:

Kies alle voor u geldende mogelijkheden:

- ☐ EMD extraheerbaarheid
- ☐ Relevantie aanbeveling

Indicateur / aanbeveling	Bron	Jaar	Evidentiegraad
**AANBEVELING:** **Leg operaties en belangrijke behandelingen bij voorkeur vast bij de relevante episode. Doe dit met de functionaliteit van uw HIS. Daarmee komt deze informatie ook in het overzicht Ingrepen en behandelingen terecht.**	**ADEPD**	**2019**	**/**

Uw oordeel:

*

Kies één van de volgende mogelijkheden:

- ◯ 1 (Slecht)
- ◯ 2
- ◯ 3
- ◯ 4
- ◯ 5
- ◯ 6
- ◯ 7
- ◯ 8
- ◯ 9 (Uitstekend)
- ◯ Niet beoordeelbaar

Beoordeling gebaseerd op:

Kies alle voor u geldende mogelijkheden:

- ☐ EMD extraheerbaarheid
- ☐ Relevantie aanbeveling

Indicator / aanbeveling	Bron	Jaar	Evidentiegraad
**AANBEVELING:** **Het EPD bevat voor relevante operaties en ingrepen een bijschrift met informatie over de procedure.**	**HIQA**	**2018**	**Geen gradatie**

Uw oordeel:

*

Kies één van de volgende mogelijkheden:

- ◯ 1 (Slecht)
- ◯ 2
- ◯ 3
- ◯ 4
- ◯ 5
- ◯ 6
- ◯ 7
- ◯ 8
- ◯ 9 (Uitstekend)
- ◯ Niet beoordeelbaar

Beoordeling gebaseerd op:

Kies alle voor u geldende mogelijkheden:

- ☐ EMD extraheerbaarheid
- ☐ Relevantie aanbeveling

Indicator / aanbeveling	Bron	Jaar	Evidentiegraad
**AANBEVELING:**<br>**Het EPD bevat voor relevante operaties en ingrepen de datum waarop deze is uitgevoerd.**	**HIQA**	**2018**	**Geen gradatie**

Uw oordeel:

*

Kies één van de volgende mogelijkheden:

- ◯ 1 (Slecht)
- ◯ 2
- ◯ 3
- ◯ 4
- ◯ 5
- ◯ 6
- ◯ 7
- ◯ 8
- ◯ 9 (Uitstekend)
- ◯ Niet beoordeelbaar

Beoordeling gebaseerd op:

Kies alle voor u geldende mogelijkheden:

- ☐ EMD extraheerbaarheid
- ☐ Relevantie aanbeveling

Indicator / aanbeveling	Bron	Jaar	Evidentiegraad
**AANBEVELING:** **Het EPD bevat een registratie indien de patiënt geen operaties of ingrepen in het verleden gehad heeft.**	**HIQA**	**2018**	**Pas de gradation**

Uw oordeel:

*

Kies één van de volgende mogelijkheden:

- ◯ 1 (Slecht)
- ◯ 2
- ◯ 3
- ◯ 4
- ◯ 5
- ◯ 6
- ◯ 7
- ◯ 8
- ◯ 9 (Uitstekend)
- ◯ Niet beoordeelbaar

Beoordeling gebaseerd op:

Kies alle voor u geldende mogelijkheden:

- ☐ EMD extraheerbaarheid
- ☐ Relevantie aanbeveling

Top 5 aanbevelingen:

Welke aanbevelingen voor de "volledigheid en adequaatheid van de probleemlijst" Vindt u het meest geschikt voor het meten van de kwaliteit voor goed gebruik van het EPD in de huisartsgeneeskunde ?

1.

*

Kies één van de volgende mogelijkheden:

- ◯ Hoeveel actieve zorgelementen heeft een patiënt gemiddeld op zijn/haar lijst met zorgelementen?
- ◯ Hoeveel procent van de probleemstatuswaardige episodes heeft inderdaad het label 'bijzondere attentiewaarde'?
- ◯ Een aantal medicamenten worden voorgeschreven specifiek in het kader van een bepaalde ziekte. Voor patiënten met een voorschrift voor deze medicatie dient een zorgelement met die diagnose aanwezig te zijn.
- ◯ Percentage van geregisteerde patiënten waarvoor er geen aanpassing was in het EPD over de voorbije 12 maanden.
- ◯ Percentage van de patiëntenpopulatie, 18 jaar en ouder, met chronische gezondheidsproblemen die ten minste een van de volgende soorten zelfmanagementondersteuning hebben ontvangen van hun zorgverlener in de eerstelijnsgezondheidszorg: Voorzien van een behandelplan ; Aangemoedigd om zelfhulpgroepen of -programma's te gebruiken
- ◯ Hoeveel procent van de episodes op de episodelijst heeft een geldige ICPC-code?
- ◯ Het is aangewezen dat communicatiebeperkingen geregistreerd staan in het EPD opdat de zorgverlener hiermee rekening kan houden.
- ◯ In het EPD wordt bijkomende informatie betreffende de diagnose best vastgelegd in bijschrift of commentaar welke meer info geeft over de aspecten van de aandoening.
- ◯ In het EPD dient de startdatum vastgelegd te worden van ieder zorgelement.
- ◯ In het EPD dient de einddatum of vermoedelijke einddatum vastgelegd te worden van ieder afgesloten zorgelement.
- ◯ In het EPD wordt dient geregistreerd te worden dat de patiënt geen gekende ziektes heeft indien de patiënt geen gekende ziektes heeft.
- ◯ Het EPD bevat: operaties en ingrepen die van belang zijn vooor de verdere hulpverlenging, , zoals bepaalde ingrepen en aandoeningen die kunnen recidiveren.
- ◯ Leg operaties en belangrijke behandelingen bij voorkeur vast bij de relevante episode.
- ◯ Het EPD bevat voor relevante operaties en ingrepen een bijschrift met informatie over de procedure.
- ◯ Het EPD bevat voor relevante operaties en ingrepen de datum waarop deze is uitgevoerd.
- ◯ Het EPD bevat een registratie indien de patiënt geen operaties of ingrepen in het verleden gehad heeft.

2.

*****

Kies één van de volgende mogelijkheden:

- ◯ Hoeveel actieve zorgelementen heeft een patiënt gemiddeld op zijn/haar lijst met zorgelementen?
- ◯ Hoeveel procent van de probleemstatuswaardige episodes heeft inderdaad het label ‘bijzondere attentiewaarde’?
- ◯ Een aantal medicamenten worden voorgeschreven specifiek in het kader van een bepaalde ziekte. Voor patiënten met een voorschrift voor deze medicatie dient een zorgelement met die diagnose aanwezig te zijn.
- ◯ Percentage van geregisteerde patiënten waarvoor er geen aanpassing was in het EPD over de voorbije 12 maanden.
- ◯ Percentage van de patiëntenpopulatie, 18 jaar en ouder, met chronische gezondheidsproblemen die ten minste een van de volgende soorten zelfmanagementondersteuning hebben ontvangen van hun zorgverlener in de eerstelijnsgezondheidszorg: Voorzien van een behandelplan ; Aangemoedigd om zelfhulpgroepen of -programma's te gebruiken
- ◯ Hoeveel procent van de episodes op de episodelijst heeft een geldige ICPC-code?
- ◯ Het is aangewezen dat communicatiebeperkingen geregistreerd staan in het EPD opdat de zorgverlener hiermee rekening kan houden.
- ◯ In het EPD wordt bijkomende informatie betreffende de diagnose best vastgelegd in bijschrift of commentaar welke meer info geeft over de aspecten van de aandoening.
- ◯ In het EPD dient de startdatum vastgelegd te worden van ieder zorgelement.
- ◯ In het EPD dient de einddatum of vermoedelijke einddatum vastgelegd te worden van ieder afgesloten zorgelement.
- ◯ In het EPD wordt dient geregistreerd te worden dat de patiënt geen gekende ziektes heeft indien de patiënt geen gekende ziektes heeft.
- ◯ Het EPD bevat: operaties en ingrepen die van belang zijn vooor de verdere hulpverlenging, , zoals bepaalde ingrepen en aandoeningen die kunnen recidiveren.
- ◯ Leg operaties en belangrijke behandelingen bij voorkeur vast bij de relevante episode.
- ◯ Het EPD bevat voor relevante operaties en ingrepen een bijschrift met informatie over de procedure.
- ◯ Het EPD bevat voor relevante operaties en ingrepen de datum waarop deze is uitgevoerd.
- ◯ Het EPD bevat een registratie indien de patiënt geen operaties of ingrepen in het verleden gehad heeft.

3.

*

Kies één van de volgende mogelijkheden:

- ◯ Hoeveel actieve zorgelementen heeft een patiënt gemiddeld op zijn/haar lijst met zorgelementen?
- ◯ Hoeveel procent van de probleemstatuswaardige episodes heeft inderdaad het label ‘bijzondere attentiewaarde’?
- ◯ Een aantal medicamenten worden voorgeschreven specifiek in het kader van een bepaalde ziekte. Voor patiënten met een voorschrift voor deze medicatie dient een zorgelement met die diagnose aanwezig te zijn.
- ◯ Percentage van geregisteerde patiënten waarvoor er geen aanpassing was in het EPD over de voorbije 12 maanden.
- ◯ Percentage van de patiëntenpopulatie, 18 jaar en ouder, met chronische gezondheidsproblemen die ten minste een van de volgende soorten zelfmanagementondersteuning hebben ontvangen van hun zorgverlener in de eerstelijnsgezondheidszorg: Voorzien van een behandelplan ; Aangemoedigd om zelfhulpgroepen of -programma's te gebruiken
- ◯ Hoeveel procent van de episodes op de episodelijst heeft een geldige ICPC-code?
- ◯ Het is aangewezen dat communicatiebeperkingen geregistreerd staan in het EPD opdat de zorgverlener hiermee rekening kan houden.
- ◯ In het EPD wordt bijkomende informatie betreffende de diagnose best vastgelegd in bijschrift of commentaar welke meer info geeft over de aspecten van de aandoening.
- ◯ In het EPD dient de startdatum vastgelegd te worden van ieder zorgelement.
- ◯ In het EPD dient de einddatum of vermoedelijke einddatum vastgelegd te worden van ieder afgesloten zorgelement.
- ◯ In het EPD wordt dient geregistreerd te worden dat de patiënt geen gekende ziektes heeft indien de patiënt geen gekende ziektes heeft.
- ◯ Het EPD bevat: operaties en ingrepen die van belang zijn vooor de verdere hulpverlenging, , zoals bepaalde ingrepen en aandoeningen die kunnen recidiveren.
- ◯ Leg operaties en belangrijke behandelingen bij voorkeur vast bij de relevante episode.
- ◯ Het EPD bevat voor relevante operaties en ingrepen een bijschrift met informatie over de procedure.
- ◯ Het EPD bevat voor relevante operaties en ingrepen de datum waarop deze is uitgevoerd.
- ◯ Het EPD bevat een registratie indien de patiënt geen operaties of ingrepen in het verleden gehad heeft.

4.

*

Kies één van de volgende mogelijkheden:

- ◯ Hoeveel actieve zorgelementen heeft een patiënt gemiddeld op zijn/haar lijst met zorgelementen?
- ◯ Hoeveel procent van de probleemstatuswaardige episodes heeft inderdaad het label ‘bijzondere attentiewaarde’?
- ◯ Een aantal medicamenten worden voorgeschreven specifiek in het kader van een bepaalde ziekte. Voor patiënten met een voorschrift voor deze medicatie dient een zorgelement met die diagnose aanwezig te zijn.
- ◯ Percentage van geregisteerde patiënten waarvoor er geen aanpassing was in het EPD over de voorbije 12 maanden.
- ◯ Percentage van de patiëntenpopulatie, 18 jaar en ouder, met chronische gezondheidsproblemen die ten minste een van de volgende soorten zelfmanagementondersteuning hebben ontvangen van hun zorgverlener in de eerstelijnsgezondheidszorg: Voorzien van een behandelplan ; Aangemoedigd om zelfhulpgroepen of -programma's te gebruiken
- ◯ Hoeveel procent van de episodes op de episodelijst heeft een geldige ICPC-code?
- ◯ Het is aangewezen dat communicatiebeperkingen geregistreerd staan in het EPD opdat de zorgverlener hiermee rekening kan houden.
- ◯ In het EPD wordt bijkomende informatie betreffende de diagnose best vastgelegd in bijschrift of commentaar welke meer info geeft over de aspecten van de aandoening.
- ◯ In het EPD dient de startdatum vastgelegd te worden van ieder zorgelement.
- ◯ In het EPD dient de einddatum of vermoedelijke einddatum vastgelegd te worden van ieder afgesloten zorgelement.
- ◯ In het EPD wordt dient geregistreerd te worden dat de patiënt geen gekende ziektes heeft indien de patiënt geen gekende ziektes heeft.
- ◯ Het EPD bevat: operaties en ingrepen die van belang zijn vooor de verdere hulpverlenging, , zoals bepaalde ingrepen en aandoeningen die kunnen recidiveren.
- ◯ Leg operaties en belangrijke behandelingen bij voorkeur vast bij de relevante episode.
- ◯ Het EPD bevat voor relevante operaties en ingrepen een bijschrift met informatie over de procedure.
- ◯ Het EPD bevat voor relevante operaties en ingrepen de datum waarop deze is uitgevoerd.
- ◯ Het EPD bevat een registratie indien de patiënt geen operaties of ingrepen in het verleden gehad heeft.

5.

*

Kies één van de volgende mogelijkheden:

- ◯ Hoeveel actieve zorgelementen heeft een patiënt gemiddeld op zijn/haar lijst met zorgelementen?
- ◯ Hoeveel procent van de probleemstatuswaardige episodes heeft inderdaad het label ‘bijzondere attentiewaarde’?
- ◯ Een aantal medicamenten worden voorgeschreven specifiek in het kader van een bepaalde ziekte. Voor patiënten met een voorschrift voor deze medicatie dient een zorgelement met die diagnose aanwezig te zijn.
- ◯ Percentage van geregisteerde patiënten waarvoor er geen aanpassing was in het EPD over de voorbije 12 maanden.
- ◯ Percentage van de patiëntenpopulatie, 18 jaar en ouder, met chronische gezondheidsproblemen die ten minste een van de volgende soorten zelfmanagementondersteuning hebben ontvangen van hun zorgverlener in de eerstelijnsgezondheidszorg: Voorzien van een behandelplan ; Aangemoedigd om zelfhulpgroepen of -programma's te gebruiken
- ◯ Hoeveel procent van de episodes op de episodelijst heeft een geldige ICPC-code?
- ◯ Het is aangewezen dat communicatiebeperkingen geregistreerd staan in het EPD opdat de zorgverlener hiermee rekening kan houden.
- ◯ In het EPD wordt bijkomende informatie betreffende de diagnose best vastgelegd in bijschrift of commentaar welke meer info geeft over de aspecten van de aandoening.
- ◯ In het EPD dient de startdatum vastgelegd te worden van ieder zorgelement.
- ◯ In het EPD dient de einddatum of vermoedelijke einddatum vastgelegd te worden van ieder afgesloten zorgelement.
- ◯ In het EPD wordt dient geregistreerd te worden dat de patiënt geen gekende ziektes heeft indien de patiënt geen gekende ziektes heeft.
- ◯ Het EPD bevat: operaties en ingrepen die van belang zijn vooor de verdere hulpverlenging, , zoals bepaalde ingrepen en aandoeningen die kunnen recidiveren.
- ◯ Leg operaties en belangrijke behandelingen bij voorkeur vast bij de relevante episode.
- ◯ Het EPD bevat voor relevante operaties en ingrepen een bijschrift met informatie over de procedure.
- ◯ Het EPD bevat voor relevante operaties en ingrepen de datum waarop deze is uitgevoerd.
- ◯ Het EPD bevat een registratie indien de patiënt geen operaties of ingrepen in het verleden gehad heeft.

Indien u nog suggesties heeft voor aanbevelingen die volgens u in bovenstaande lijst ontbreken, mag u deze in onderstaande vakje noteren.

Gelieve steeds duidelijk een aanbeveling en motivatie te noteren voor deze aanbeveling.

Vul uw antwoord hier in:

2. Gestructureerd registreren in EPD

In welke mate zijn de volgende aanbevelingen relevant voor het meten van de kwaliteit van het goed gebruik van het EMD in de huisartsgeneeskunde in verband met het **gestructureerd registreren in het EPD**?

Indicator / aanbeveling	Bron	Jaar	Evidentiegraad
**INDICATOR:**<br>**Hoeveel procent van de deelcontacten in het journaal gekoppeld aan zorgelementen met een geldige ICPC-code?**<br>**ICPC code verder te verdelen in :**<br>• **Correct gebruik: Klachten in de range 01 tot en met 29 en diagnoses in de range 70 tot en met 99. In deze categorie zijn ook opgenomen A44 (inenting), R44 (influenzavaccinatie) en X37 (cervix-uitstrijkje bevolkingsonderzoek);**<br>• **Mogelijk incorrect gebruik: A97 (geen ziekte) of A99 (andere gegeneraliseerde of niet gespecificeerde ziekte) (als vluchtcode);**<br>• **Niet-toegestane of geen ICPC: Geen ICPC of een niet toegestane code (range 30-69), met uitzondering van A44 (preventieve inenting of medicatie), R44 (influenzavaccinatie) en X37 (cervixuitstrijkje bevolkingsonderzoek).**	**EPD-scan-h**	**2009**	**Geen gradatie**

Uw oordeel:

*

Kies één van de volgende mogelijkheden:

- ◯ 1 (Slecht)
- ◯ 2
- ◯ 3
- ◯ 4
- ◯ 5
- ◯ 6
- ◯ 7
- ◯ 8
- ◯ 9 (Uitstekend)
- ◯ Niet beoordeelbaar

Beoordeling gebaseerd op:

Kies alle voor u geldende mogelijkheden:

- [ ] EMD extraheerbaarheid
- [ ] Relevantie aanbeveling

Indicator / aanbeveling	Bron	Jaar	Evidentiegraad
**INDICATOR:**<br><br>**Hoeveel procent van de deelcontacten die zijn vastgelegd tijdens consulten, telefonische contacten en huisbezoeken hebben een Subjectief-, Objectief-, Evaluatie- of Planning-regel?**	**EPD-scan-h**	**2009**	**Geen gradatie**
**GEKOPPELDE AANBEVELING:**<br><br>**Deelcontactverslag: het EPD presenteert SOEP-regels van**<br>**het deelcontact van waaruit de huisarts verwijst.**<br>**De huisarts selecteert zo nodig extra deelcontacten van de episode.**	**HASP**	**2017**	**Geen gradatie**

Uw oordeel:

*

Kies één van de volgende mogelijkheden:

- ◯ 1 (Slecht)
- ◯ 2
- ◯ 3
- ◯ 4
- ◯ 5
- ◯ 6
- ◯ 7
- ◯ 8
- ◯ 9 (Uitstekend)
- ◯ Niet beoordeelbaar

Beoordeling gebaseerd op:

Kies alle voor u geldende mogelijkheden:

- [ ] EMD extraheerbaarheid
- [ ] Relevantie aanbeveling

Indicator / Aanbeveling	Bron	Jaar	Evidentiegraad
**INDICATOR:** **Aantal raadplegingen voor online bronnen (vb. CDLH)**	**Hamade et al.**	**2008**	**Geen gradatie**

Uw oordeel:

*

Kies één van de volgende mogelijkheden:

- ◯ 1 (Slecht)
- ◯ 2
- ◯ 3
- ◯ 4
- ◯ 5
- ◯ 6
- ◯ 7
- ◯ 8
- ◯ 9 (Uitstekend)
- ◯ Niet beoordeelbaar

Beoordeling gebaseerd op:

Kies alle voor u geldende mogelijkheden:

- [ ] EMD extraheerbaarheid
- [ ] Relevantie aanbeveling

Indicator / aanbeveling	Bron	Jaar	Evidentiegraad
**INDICATOR:** **Aantal veranderingen in planningen per week per 1000 GMD patiënten**	**Hamade et al.**	**2008**	**Geen gradatie**
**GEKOPPELDE AANBEVELING:** **Encounter forms or notes have a notation, regarding follow-up care, calls or visits, when indicated. The specific time of return is noted in weeks, months or as needed.**	**NCQA**	**2018**	**Geen gradatie**

Uw oordeel:

*

Kies één van de volgende mogelijkheden:

- ◯ 1 (Slecht)
- ◯ 2
- ◯ 3
- ◯ 4
- ◯ 5
- ◯ 6
- ◯ 7
- ◯ 8
- ◯ 9 (Uitstekend)
- ◯ Niet beoordeelbaar

Beoordeling gebaseerd op:

Kies alle voor u geldende mogelijkheden:

- ☐ EMD extraheerbaarheid
- ☐ Relevantie aanbeveling

Indicator / aanbeveling	Bron	Jaar	Evidentiegraad
**AANBEVELING:** **Consultation, laboratory and imaging reports filed in the chart are initialed by the practitioner who ordered them, to signify review. (Review and signature by professionals other than the ordering practitioner do not meet this requirement.) If the reports are presented electronically or by some other method, there is also representation of review by the ordering practitioner. Consultation and abnormal laboratory and imaging study results have an explicit notation in the record of followup plans.**	**NCQA**	**2018**	**Geen gradatie**

Uw oordeel:

*

Kies één van de volgende mogelijkheden:

- ◯ 1 (Slecht)
- ◯ 2
- ◯ 3
- ◯ 4
- ◯ 5
- ◯ 6
- ◯ 7
- ◯ 8
- ◯ 9 (Uitstekend)
- ◯ Niet beoordeelbaar

Beoordeling gebaseerd op:

Kies alle voor u geldende mogelijkheden:

- ☐ EMD extraheerbaarheid
- ☐ Relevantie aanbeveling

Top 5 aanbevelingen:

Welke aanbevelingen voor de "compleetheid en actualiteit medicatie-overzicht" Vindt u het meest geschikt voor het meten van de kwaliteit voor goed gebruik van het EPD in de huisartsgeneeskunde ?

1.

*

Kies één van de volgende mogelijkheden:

- ◯ Hoeveel procent van de deelcontacten in het journaal gekoppeld aan zorgelementen met een geldige ICPC-code?
- ◯ Hoeveel procent van de deelcontacten die zijn vastgelegd tijdens consulten, telefonische contacten en huisbezoeken hebben een Subjectief-, Objectief-, Evaluatie- of Planning-regel?
- ◯ Aantal raadplegingen voor online bronnen (vb. CDLH)
- ◯ Aantal veranderingen in planningen per week per 1000 GMD patiënten
- ◯ Consultation, laboratory and imaging reports filed in the chart are initialed by the practitioner who ordered them, to signify review

2.

*****

Kies één van de volgende mogelijkheden:

- ◯ Hoeveel procent van de deelcontacten in het journaal gekoppeld aan zorgelementen met een geldige ICPC-code?
- ◯ Hoeveel procent van de deelcontacten die zijn vastgelegd tijdens consulten, telefonische contacten en huisbezoeken hebben een Subjectief-, Objectief-, Evaluatie- of Planning-regel?
- ◯ Aantal raadplegingen voor online bronnen (vb. CDLH)
- ◯ Aantal veranderingen in planningen per week per 1000 GMD patiënten
- ◯ Consultation, laboratory and imaging reports filed in the chart are initialed by the practitioner who ordered them, to signify review

3.

*****

Kies één van de volgende mogelijkheden:

- ◯ Hoeveel procent van de deelcontacten in het journaal gekoppeld aan zorgelementen met een geldige ICPC-code?
- ◯ Hoeveel procent van de deelcontacten die zijn vastgelegd tijdens consulten, telefonische contacten en huisbezoeken hebben een Subjectief-, Objectief-, Evaluatie- of Planning-regel?
- ◯ Aantal raadplegingen voor online bronnen (vb. CDLH)
- ◯ Aantal veranderingen in planningen per week per 1000 GMD patiënten
- ◯ Consultation, laboratory and imaging reports filed in the chart are initialed by the practitioner who ordered them, to signify review

Indien u nog suggesties heeft voor aanbevelingen die volgens u in bovenstaande lijst ontbreken, mag u deze in onderstaande vakje noteren.

Gelieve steeds duidelijk een aanbeveling en motivatie te noteren voor deze aanbeveling.

Vul uw antwoord hier in:

3. Compleetheid en actualiteit medicatie-overzicht

In welke mate zijn de volgende aanbevelingen relevant voor het meten van de kwaliteit van het goed gebruik van het EMD in de huisartsgeneeskunde in verband met de **compleetheid en actualiteit van het medicatie-overzicht** van patiënten?

Indicator / Aanbeveling	Bron	Jaar	Evidentiegraad
**INDICATOR:** **Hoeveel procent van de medicatie op de lijst 'actuele medicatie' is onterecht als actuele medicatie gelabeld?** **In de professionele samenvatting moet een overzicht staan van de actuele medicatie (dat wil zeggen, de medicatie die de patiënt op dat moment gebruikt, tijdelijk of chronisch (> 6 maand)) en de medicatie die de afgelopen maanden is gestopt (4-6 maanden afhankelijk van bron). In de regel schrijft een huisarts geneesmiddelen voor drie maanden voor, met uitzonderingen naar zes maanden voor bijvoorbeeld de pil. Medicatie die langer dan 6 maanden niet is voorgeschreven is vermoedelijk niet meer actueel.**	**EPD-scan-h**	**2009**	**Geen gradatie**
**GEKOPPELDE AANBEVELING:** **Het EPD presenteert de actuele voorschriften. Het HIS geeft de mogelijkheid toe te voegen eerdere medicatie bij de episode van waaruit wordt verwezen incl. reden stoppen, en/of zelfmedicatie.**	**ADEPD, Domus Medica, SSMG, NHS, HIQA**	**2019, 2004, ?, 2023, 2020**	**Geen gradatie**

Uw oordeel:

*

Kies één van de volgende mogelijkheden:

- ◯ 1 (Slecht)
- ◯ 2
- ◯ 3
- ◯ 4
- ◯ 5
- ◯ 6
- ◯ 7
- ◯ 8
- ◯ 9 (Uitstekend)
- ◯ Niet beoordeelbaar

Beoordeling gebaseerd op:

Kies alle voor u geldende mogelijkheden:

- ☐ EMD extraheerbaarheid
- ☐ Relevantie aanbeveling

Indicator / Aanbeveling	Bron	Jaar	Evidentiegraad
**INDICATOR:**<br>**Hoeveel procent van de voorschriften is gekoppeld aan een episode?**<br>**Voor deze indicator selecteerden we de actuele medicatie en de medicatie die de afgelopen vier maanden is gestopt.**<br>**ICPC code verder te verdelen in :**<br>• **Correct gebruik: Klachten in de range 01 tot en met 29 en diagnoses in de range 70 tot en met 99. In deze categorie zijn ook opgenomen A44 (inenting), R44 (influenzavaccinatie) en X37 (cervix-uitstrijkje bevolkingsonderzoek)**<br>• **Mogelijk incorrect gebruik: A97 (geen ziekte) of A99 (andere gegeneraliseerde of niet gespecificeerde ziekte) (als vluchtcode);**<br>• **Niet-toegestane of geen ICPC: Geen ICPC of een niet toegestane code (range 30-69), met uitzondering van A44 (preventieve inenting of medicatie), R44 (influenzavaccinatie) en X37 (cervixuitstrijkje bevolkingsonderzoek).**	**EPD-scan-h**	**2009**	**Geen gradatie**

Uw oordeel:

*

Kies één van de volgende mogelijkheden:

- ◯ 1 (Slecht)
- ◯ 2
- ◯ 3
- ◯ 4
- ◯ 5
- ◯ 6
- ◯ 7
- ◯ 8
- ◯ 9 (Uitstekend)
- ◯ Niet beoordeelbaar

Beoordeling gebaseerd op:

Kies alle voor u geldende mogelijkheden:

- ☐ EMD extraheerbaarheid
- ☐ Relevantie aanbeveling

Indicator / Aanbeveling	Bron	Jaar	Evidentiegraad
**INDICATOR:**<br>**Volledige dosis- en behandelingsschema met betrekking tot dosis-effect of bijwerkingen van medicatie.**	**De Lusignan et al**	**2002**	**Geen gradatie**
**GELINKTE AANBEVELING:**<br>• **Voor elk voorgeschreven medicament dient de posologie geregistreerd te zijn in het EPD.**<br>• **Voor elk voorgeschreven medicament dient het innamemoment geregistreerd te zijn in het EPD.**<br>• **Voor elk voorgeschreven medicament dient de duur van toediening geregistreerd te zijn in het EPD.**<br>• **Voor elk voorgeschreven medicament dient wijze van toediening geregistreerd te zijn in het EPD.**<br>• **Voor elk voorgeschreven medicament dient de startdatum geregistreerd te zijn in het EPD.**	**HIQA**	**2018**	**Geen gradatie**

Uw oordeel:

*

Kies één van de volgende mogelijkheden:

◯ 1 (Slecht)

◯ 2

- ◯ 3
- ◯ 4
- ◯ 5
- ◯ 6
- ◯ 7
- ◯ 8
- ◯ 9 (Uitstekend)
- ◯ Niet beoordeelbaar

Beoordeling gebaseerd op:

Kies alle voor u geldende mogelijkheden:

- ☐ EMD extraheerbaarheid
- ☐ Relevantie aanbeveling

Indicator / aanbeveling	Bron	Jaar	Evidentiegraad
**INDICATOR:** **Aantal voorschriften voor medicatie per week per 1 000 GMD patiënten?** **Dit is een ruwe maatstaf voor hoeveel voorschriften niet digitaal worden opgesteld. Alsook een detectietool voor ontbrekende data in de tijd.**	**De Lusignan.**	**2002**	**Geen gradatie**

Uw oordeel:

*

Kies één van de volgende mogelijkheden:

- ◯ 1 (Slecht)
- ◯ 2
- ◯ 3
- ◯ 4
- ◯ 5
- ◯ 6
- ◯ 7
- ◯ 8
- ◯ 9 (Uitstekend)
- ◯ Niet beoordeelbaar

Beoordeling gebaseerd op:

Kies alle voor u geldende mogelijkheden:

- ☐ EMD extraheerbaarheid
- ☐ Relevantie aanbeveling

Indicator / Aanbeveling	Bron	Jaar	Evidentiegraad
**AANBEVELING:** **Indien de patiënt geen medicatie neemt dient dit correct genoteerd te zijn in het EPD.**	**HIQA**	**2018**	**Geen gradatie**

Uw oordeel:

*

Kies één van de volgende mogelijkheden:

- ◯ 1 (Slecht)
- ◯ 2
- ◯ 3
- ◯ 4
- ◯ 5
- ◯ 6
- ◯ 7
- ◯ 8
- ◯ 9 (Uitstekend)
- ◯ Niet beoordeelbaar

Beoordeling gebaseerd op:

Kies alle voor u geldende mogelijkheden:

- ☐ EMD extraheerbaarheid
- ☐ Relevantie aanbeveling

Top 3 aanbevelingen:

Welke aanbevelingen voor de "compleetheid en actualiteit medicatie-overzicht" Vindt u het meest geschikt voor het meten van de kwaliteit voor goed gebruik van het EPD in de huisartsgeneeskunde ?

1.

*

Kies één van de volgende mogelijkheden:

- ◯ Hoeveel procent van de medicatie op de lijst 'actuele medicatie' is onterecht als actuele medicatie gelabeld?
- ◯ Hoeveel procent van de voorschriften is gekoppeld aan een episode?
- ◯ Volledige dosis- en behandelingsschema met betrekking tot dosis-effect of bijwerkingen van medicatie.
- ◯ Indien de patiënt geen medicatie neemt dient dit correct genoteerd te zijn in het EPD.
- ◯ Aantal voorschriften voor medicatie per week per 1 000 GMD patiënten?

2.

*

Kies één van de volgende mogelijkheden:

- ◯ Hoeveel procent van de medicatie op de lijst 'actuele medicatie' is onterecht als actuele medicatie gelabeld?
- ◯ Hoeveel procent van de voorschriften is gekoppeld aan een episode?
- ◯ Volledige dosis- en behandelingsschema met betrekking tot dosis-effect of bijwerkingen van medicatie.
- ◯ Indien de patiënt geen medicatie neemt dient dit correct genoteerd te zijn in het EPD.
- ◯ Aantal voorschriften voor medicatie per week per 1 000 GMD patiënten?

3.

*

Kies één van de volgende mogelijkheden:

- ◯ Hoeveel procent van de medicatie op de lijst 'actuele medicatie' is onterecht als actuele medicatie gelabeld?
- ◯ Hoeveel procent van de voorschriften is gekoppeld aan een episode?
- ◯ Volledige dosis- en behandelingsschema met betrekking tot dosis-effect of bijwerkingen van medicatie.
- ◯ Indien de patiënt geen medicatie neemt dient dit correct genoteerd te zijn in het EPD.
- ◯ Aantal voorschriften voor medicatie per week per 1 000 GMD patiënten?

Indien u nog suggesties heeft voor aanbevelingen die volgens u in bovenstaande lijst ontbreken, mag u deze in onderstaande vakje noteren.

Gelieve steeds duidelijk een aanbeveling en motivatie te noteren voor deze aanbeveling.

Vul uw antwoord hier in:

4. Risicofactoren / Medicatiebewaking

In welke mate zijn de volgende aanbevelingen relevant voor het meten van de kwaliteit van het goed gebruik van het EMD in de huisartsgeneeskunde in verband met de **risicofactoren en medicatiebewaking van patiënten**?

Indicator / aanbeveling	Bron	Jaar	Evidentiegraad
**INDICATOR:** **Aantal nieuwe planningen voor screening voor dikkedarmkanker of borstkanker per week per 1000 GMD patiënten**	**Hamade et al.**	**2008**	**Geen evidentiegraad**

Uw oordeel:

*

Kies één van de volgende mogelijkheden:

- ◯ 1 (Slecht)
- ◯ 2
- ◯ 3
- ◯ 4
- ◯ 5
- ◯ 6
- ◯ 7
- ◯ 8
- ◯ 9 (Uitstekend)
- ◯ Niet beoordeelbaar

Beoordeling gebaseerd op:

Kies alle voor u geldende mogelijkheden:

- [ ] EMD extraheerbaarheid
- [ ] Relevantie aanbeveling

Indicator / Aanbeveling	Bron	Jaar	Evidentiegraad
**INDICATOR:**<br>**Van hoeveel patiënten is een contra-indicatie medicatie geregistreerd?**<br>**Contra-indicaties zijn aandoeningen waarmee bij het voorschrijven van medicatie rekening dient te worden gehouden. Te denken valt aan bijvoorbeeld diabetes mellitus of nierinsufficiëntie. Het kan ook gaan om tijdelijke contra-indicaties zoals bijvoorbeeld bij zwangerschap.**	**EPD-scan-h**	**2009**	**Geen evidentiegraad**
**GEKOPPELDE AANBEVELING:**<br>**Overgevoeligheid voor medicatie, contra-indicaties voor voorschrijven. Het EPD presenteert vanuit medicatie-overgevoeligheid respectievelijk contra-indicatie.**	**ADEPD**	**2019**	**Geen gradatie**

Uw oordeel:

*

Kies één van de volgende mogelijkheden:

- ◯ 1 (Slecht)
- ◯ 2
- ◯ 3
- ◯ 4
- ◯ 5
- ◯ 6
- ◯ 7
- ◯ 8

◯ 9 (Uitstekend)

◯ Niet beoordeelbaar

Beoordeling gebaseerd op:

Kies alle voor u geldende mogelijkheden:

☐ EMD extraheerbaarheid

☐ Relevantie aanbeveling

Indicator / Aanbeveling	Bron	Jaar	Evidentiegraad
**INDICATOR:**<br>**Van hoeveel patiënten is een geneesmiddelenallergie of -intolerantie geregistreerd?**	**EPD-scan-h**	**2008**	**Geen evidentiegraad**
**GEKOPPELDE AANBEVELING:**<br>**The patient summary SHALL identify the substance that the patient has a susceptibility to an allergy upon exposure to the substance. It includes allergies, intolerances and adverse reactions to all substances,not only those arising from medications or medicines. It also describes other clinical information that is imperative to know so that the life or health of the patient does not come under threat. For example, intolerance to aspirin due to gastrointestinal bleeding.**<br>**The patient summary SHOULD describe the type of reaction event as determined by the healthcare practitioner.**<br>**The patient summary SHOULD include the severity of the symptom as determined by the healthcare practitioner.**<br>**The patient summary SHOULD contain a record**	**HIQA**	**2020**	**Geen gradatie**

Indicator / Aanbeveling	Bron	Jaar	Evidentiegraad
**of the date and or time of the onset of the reaction.**			

Uw oordeel:

*****

Kies één van de volgende mogelijkheden:

- ◯ 1 (Slecht)
- ◯ 2
- ◯ 3
- ◯ 4
- ◯ 5
- ◯ 6
- ◯ 7
- ◯ 8
- ◯ 9 (Uitstekend)
- ◯ Niet beoordeelbaar

Beoordeling gebaseerd op:

Kies alle voor u geldende mogelijkheden:

- ☐ EMD extraheerbaarheid
- ☐ Relevantie aanbeveling

Indicator / Aanbeveling	Bron	Jaar	Evidentiegraad
**AANBEVELING:** **Bepaalde medische informatie is van belang voor de hele zorgketen. NHG beveelt registratie aan van: profylaxe voor addison crisis, endocarditis, bloedingsziekten, endoprothese, immuungecompromitteerde patiënt, (functionele) asplenie, trombose of bijzonder resistente micro-organismen.**	**ADEPD**	**2019**	**Geen gradatie**

Uw oordeel:

*

Kies één van de volgende mogelijkheden:

- ◯ 1 (Slecht)
- ◯ 2
- ◯ 3
- ◯ 4
- ◯ 5
- ◯ 6
- ◯ 7
- ◯ 8
- ◯ 9 (Uitstekend)
- ◯ Niet beoordeelbaar

Beoordeling gebaseerd op:

Kies alle voor u geldende mogelijkheden:

- ☐ EMD extraheerbaarheid
- ☐ Relevantie aanbeveling

Indicator / aanbeveling	Bron	Jaar	Evidentiegraad
**AANBEVELING:**<br>**Lichamelijk onderzoek kan in vrije tekst of als diagnostische bepaling in het EPD worden vastgelegd. Indien het als diagnostische bepaling is vastgelegd, kan het worden hergebruikt voor overzichten in de tijd en voor beslisondersteuning. Waar mogelijk dient het lichamelijk onderzoek dan ook zo veel mogelijk als diagnostische bepaling worden vastgelegd in het EPD.**<br>**Diagnostische bepalingen zijn bijvoorbeeld: gewicht, lengte, bloeddruk, hartritme, gebruik van alcohol en nicotine, middelengebruik, hoeveelheid lichaamsbeweging...**<br>**Een volledige lijst kan u vinden op: NHG - Bepalingenviewer**	**ADEPD**	**2019**	**Geen gradatie**

Uw oordeel:

*

Kies één van de volgende mogelijkheden:

- ◯ 1 (Slecht)
- ◯ 2
- ◯ 3
- ◯ 4
- ◯ 5
- ◯ 6
- ◯ 7
- ◯ 8
- ◯ 9 (Uitstekend)

◯ Niet beoordeelbaar

Beoordeling gebaseerd op:

Kies alle voor u geldende mogelijkheden:

☐ EMD extraheerbaarheid
☐ Relevantie aanbeveling

Indicator / aanbeveling	Bron	Jaar	Evidentiegraad
**AANBEVELING:** **Voor alle patiënten 12 jaar en ouder is een een vermelding in het EMD in verband met het gebruik van cigaretten, alcohol en middelen.**	**NCQA, HASP, NHS**	**2018, 2017, 2023**	**Geen evidentiegraad**

Uw oordeel:

*

Kies één van de volgende mogelijkheden:

◯ 1 (Slecht)
◯ 2
◯ 3
◯ 4
◯ 5
◯ 6
◯ 7
◯ 8
◯ 9 (Uitstekend)
◯ Niet beoordeelbaar

Beoordeling gebaseerd op:

Kies alle voor u geldende mogelijkheden:

☐ EMD extraheerbaarheid
☐ Relevantie aanbeveling

Indicator / Aanbeveling	Bron	Jaar	Evidentiegraad
**AANBEVELING:** **Indien in het EPD het dossierdeel Familieanamnese aanwezig is: leg hierin de informatie vast over de aandoeningen die voorkomen bij bloedverwanten, per aandoening bij welke familieleden deze voorkomt, op welke leeftijd het bij hen is begonnen en eventueel op welke leeftijd zij daaraan zijn overleden; - de bron van deze informatie is vaak de patiënt zelf - laat de velden open als de informatie niet duidelijk of onbekend is**	**ADEPH**	**2019**	**Geen evidentiegraad**

Uw oordeel:

*

Kies één van de volgende mogelijkheden:

- ◯ 1 (Slecht)
- ◯ 2
- ◯ 3
- ◯ 4
- ◯ 5
- ◯ 6
- ◯ 7
- ◯ 8
- ◯ 9 (Uitstekend)
- ◯ Niet beoordeelbaar

Beoordeling gebaseerd op:

Kies alle voor u geldende mogelijkheden:

- [ ] EMD extraheerbaarheid
- [ ] Relevantie aanbeveling

Indicator / Aanbeveling	Bron	Jaar	Evidentiegraad
**AANBEVELING:** **Bij patiënten zonder gekende geneesmiddelenallergie of -intolerantie dient dit genoteerd te zijn in het EPD.**	**NCQA HIQA**	**2018 2020**	**Geen evidentiegraad**

Uw oordeel:

*

Kies één van de volgende mogelijkheden:

- ○ 1 (Slecht)
- ○ 2
- ○ 3
- ○ 4
- ○ 5
- ○ 6
- ○ 7
- ○ 8
- ○ 9 (Uitstekend)
- ○ Niet beoordeelbaar

Beoordeling gebaseerd op:

Kies alle voor u geldende mogelijkheden:

- [ ] EMD extraheerbaarheid
- [ ] Relevantie aanbeveling

Indicator / Aanbeveling	Bron	Jaar	Evidentiegraad
**AANBEVELING:** **Psychogeriatrisch onderzoek: indien het een kwetsbare betreft selecteert wordt dit genoteerd in het EPD in de afgesproken rubrieken rond kwetsbaarheid.**	**HASP**	**2017**	**Geen evidentiegraad**

Uw oordeel:

*

Kies één van de volgende mogelijkheden:

- ◯ 1 (Slecht)
- ◯ 2
- ◯ 3
- ◯ 4
- ◯ 5
- ◯ 6
- ◯ 7
- ◯ 8
- ◯ 9 (Uitstekend)
- ◯ Niet beoordeelbaar

Beoordeling gebaseerd op:

Kies alle voor u geldende mogelijkheden:

- ☐ EMD extraheerbaarheid
- ☐ Relevantie aanbeveling

Indicator / Aanbeveling	Bron	Jaar	Evidentiegraad
**AANBEVELING:** **Sociale gegevens worden vastgelegd in het dossierdeel Sociale gegevens. Onder sociale gegevens worden verstaan: opleiding, beroep, werk en sociaaleconomische status. Ook taal- en taalvaardigheid, gezondheidsvaardigheden, woonsituatie, gezinssituatie, aanwezigheid van mantelzorg, migratiehistorie, overtuigingen en heftige emotionele gebeurtenissen in het verleden vallen onder sociale gegevens**	**ADEPD**	**2019**	**Geen evidentiegraad**

Uw oordeel:

*

Kies één van de volgende mogelijkheden:

- ◯ 1 (Slecht)
- ◯ 2
- ◯ 3
- ◯ 4
- ◯ 5
- ◯ 6
- ◯ 7
- ◯ 8
- ◯ 9 (Uitstekend)
- ◯ Niet beoordeelbaar

Beoordeling gebaseerd op:

Kies alle voor u geldende mogelijkheden:

- ☐ EMD extraheerbaarheid
- ☐ Relevantie aanbeveling

Top 5 aanbevelingen:

Welke aanbevelingen voor de "risicofactoren / medicatiebewaking" Vindt u het meest geschikt voor het meten van de kwaliteit voor goed gebruik van het EPD in de huisartsgeneeskunde ?

1.

*

Kies één van de volgende mogelijkheden:

- ◯ Aantal nieuwe planningen voor screening voor dikkedarmkanker of borstkanker per week per 1000 GMD patiënten
- ◯ Van hoeveel patiënten is een contra-indicatie medicatie geregistreerd?
- ◯ Van hoeveel patiënten is een geneesmiddelenallergie of -intolerantie geregistreerd?
- ◯ NHG beveelt registratie aan van: profylaxe voor addison crisis, endocarditis, bloedingsziekten, endoprothese, immuungecompromitteerde patiënt, (functionele) asplenie, trombose of bijzonder resistente micro-organismen.
- ◯ Waar mogelijk dient het lichamelijk onderzoek dan ook zo veel mogelijk als diagnostische bepaling worden vastgelegd in het EPD.
- ◯ Voor alle patiënten 12 jaar en ouder is een een vermelding in het EMD in verband met het gebruik van cigaretten, alcohol en middelen.
- ◯ Indien in het EPD het dossierdeel Familieanamnese aanwezig is: leg hierin de informatie vast over de aandoeningen die voorkomen bij bloedverwanten
- ◯ Bij patiënten zonder gekende geneesmiddelenallergie of -intolerantie dient dit genoteerd te zijn in het EPD.
- ◯ Psychogeriatrisch onderzoek: indien het een kwetsbare betreft selecteert wordt dit genoteerd in het EPD in de afgesproken rubrieken rond kwetsbaarheid.
- ◯ Sociale gegevens worden vastgelegd in het dossierdeel Sociale gegevens

2.

*

Kies één van de volgende mogelijkheden:

- ◯ Aantal nieuwe planningen voor screening voor dikkedarmkanker of borstkanker per week per 1000 GMD patiënten
- ◯ Van hoeveel patiënten is een contra-indicatie medicatie geregistreerd?
- ◯ Van hoeveel patiënten is een geneesmiddelenallergie of -intolerantie geregistreerd?
- ◯ NHG beveelt registratie aan van: profylaxe voor addison crisis, endocarditis, bloedingsziekten, endoprothese, immuungecompromitteerde patiënt, (functionele) asplenie, trombose of bijzonder resistente micro-organismen.
- ◯ Waar mogelijk dient het lichamelijk onderzoek dan ook zo veel mogelijk als diagnostische bepaling worden vastgelegd in het EPD.
- ◯ Voor alle patiënten 12 jaar en ouder is een een vermelding in het EMD in verband met het gebruik van cigaretten, alcohol en middelen.
- ◯ Indien in het EPD het dossierdeel Familieanamnese aanwezig is: leg hierin de informatie vast over de aandoeningen die voorkomen bij bloedverwanten
- ◯ Bij patiënten zonder gekende geneesmiddelenallergie of -intolerantie dient dit genoteerd te zijn in het EPD.
- ◯ Psychogeriatrisch onderzoek: indien het een kwetsbare betreft selecteert wordt dit genoteerd in het EPD in de afgesproken rubrieken rond kwetsbaarheid.
- ◯ Sociale gegevens worden vastgelegd in het dossierdeel Sociale gegevens

3.

*

Kies één van de volgende mogelijkheden:

- ◯ Aantal nieuwe planningen voor screening voor dikkedarmkanker of borstkanker per week per 1000 GMD patiënten
- ◯ Van hoeveel patiënten is een contra-indicatie medicatie geregistreerd?
- ◯ Van hoeveel patiënten is een geneesmiddelenallergie of -intolerantie geregistreerd?
- ◯ NHG beveelt registratie aan van: profylaxe voor addison crisis, endocarditis, bloedingsziekten, endoprothese, immuungecompromitteerde patiënt, (functionele) asplenie, trombose of bijzonder resistente micro-organismen.
- ◯ Waar mogelijk dient het lichamelijk onderzoek dan ook zo veel mogelijk als diagnostische bepaling worden vastgelegd in het EPD.
- ◯ Voor alle patiënten 12 jaar en ouder is een een vermelding in het EMD in verband met het gebruik van cigaretten, alcohol en middelen.
- ◯ Indien in het EPD het dossierdeel Familieanamnese aanwezig is: leg hierin de informatie vast over de aandoeningen die voorkomen bij bloedverwanten
- ◯ Bij patiënten zonder gekende geneesmiddelenallergie of -intolerantie dient dit genoteerd te zijn in het EPD.
- ◯ Psychogeriatrisch onderzoek: indien het een kwetsbare betreft selecteert wordt dit genoteerd in het EPD in de afgesproken rubrieken rond kwetsbaarheid.
- ◯ Sociale gegevens worden vastgelegd in het dossierdeel Sociale gegevens

4.

*

Kies één van de volgende mogelijkheden:

◯ Aantal nieuwe planningen voor screening voor dikkedarmkanker of borstkanker per week per 1000 GMD patiënten

◯ Van hoeveel patiënten is een contra-indicatie medicatie geregistreerd?

◯ Van hoeveel patiënten is een geneesmiddelenallergie of -intolerantie geregistreerd?

◯ NHG beveelt registratie aan van: profylaxe voor addison crisis, endocarditis, bloedingsziekten, endoprothese, immuungecompromitteerde patiënt, (functionele) asplenie, trombose of bijzonder resistente micro-organismen.

◯ Waar mogelijk dient het lichamelijk onderzoek dan ook zo veel mogelijk als diagnostische bepaling worden vastgelegd in het EPD.

◯ Voor alle patiënten 12 jaar en ouder is een een vermelding in het EMD in verband met het gebruik van cigaretten, alcohol en middelen.

◯ Indien in het EPD het dossierdeel Familieanamnese aanwezig is: leg hierin de informatie vast over de aandoeningen die voorkomen bij bloedverwanten

◯ Bij patiënten zonder gekende geneesmiddelenallergie of -intolerantie dient dit genoteerd te zijn in het EPD.

◯ Psychogeriatrisch onderzoek: indien het een kwetsbare betreft selecteert wordt dit genoteerd in het EPD in de afgesproken rubrieken rond kwetsbaarheid.

◯ Sociale gegevens worden vastgelegd in het dossierdeel Sociale gegevens

5.

*

Kies één van de volgende mogelijkheden:

◯ Aantal nieuwe planningen voor screening voor dikkedarmkanker of borstkanker per week per 1000 GMD patiënten

◯ Van hoeveel patiënten is een contra-indicatie medicatie geregistreerd?

◯ Van hoeveel patiënten is een geneesmiddelenallergie of -intolerantie geregistreerd?

◯ NHG beveelt registratie aan van: profylaxe voor addison crisis, endocarditis, bloedingsziekten, endoprothese, immuungecompromitteerde patiënt, (functionele) asplenie, trombose of bijzonder resistente micro-organismen.

◯ Waar mogelijk dient het lichamelijk onderzoek dan ook zo veel mogelijk als diagnostische bepaling worden vastgelegd in het EPD.

◯ Voor alle patiënten 12 jaar en ouder is een een vermelding in het EMD in verband met het gebruik van cigaretten, alcohol en middelen.

◯ Indien in het EPD het dossierdeel Familieanamnese aanwezig is: leg hierin de informatie vast over de aandoeningen die voorkomen bij bloedverwanten

◯ Bij patiënten zonder gekende geneesmiddelenallergie of -intolerantie dient dit genoteerd te zijn in het EPD.

◯ Psychogeriatrisch onderzoek: indien het een kwetsbare betreft selecteert wordt dit genoteerd in het EPD in de afgesproken rubrieken rond kwetsbaarheid.

◯ Sociale gegevens worden vastgelegd in het dossierdeel Sociale gegevens

Indien u nog suggesties heeft voor aanbevelingen die volgens u in bovenstaande lijst ontbreken, mag u deze in onderstaande vakje noteren.

Gelieve steeds duidelijk een aanbeveling en motivatie te noteren voor deze aanbeveling.

Vul uw antwoord hier in:

5. Patiëntenidentificatie / Contactinformatie

In welke mate zijn de volgende aanbevelingen relevant voor het meten van de kwaliteit van het goed gebruik van het EMD in de huisartsgeneeskunde in verband met de **patiëntenidentificatie en de contactinformatie in het EPD**?

Indicator / aanbeveling	Bron	Jaar	Evidentiegraad
**AANBEVELING:** **In het EPD dient geregistreerd te zijn in welke urgentiedienst (ziekenhuis) de patiënt gekend is.**	**NHS**	**2023**	**Geen gradatie**

Uw oordeel:

*

Kies één van de volgende mogelijkheden:

- 1 (Slecht)
- 2
- 3
- 4
- 5
- 6
- 7
- 8
- 9 (Uitstekend)
- Niet beoordeelbaar

Beoordeling gebaseerd op:

Kies alle voor u geldende mogelijkheden:

- [ ] EMD extraheerbaarheid
- [ ] Relevantie aanbeveling

Indicator / aanbeveling	Bron	Jaar	Evidentiegraad
**AANBEVELING:** **In het EPD dient geregistreerd te zijn of de patiënt gehospitaliseerd is**	**NHS**	**2023**	**Geen gradatie**

Uw oordeel:

*

Kies één van de volgende mogelijkheden:

- ○ 1 (Slecht)
- ○ 2
- ○ 3
- ○ 4
- ○ 5
- ○ 6
- ○ 7
- ○ 8
- ○ 9 (Uitstekend)
- ○ Niet beoordeelbaar

Beoordeling gebaseerd op:

Kies alle voor u geldende mogelijkheden:

- [ ] EMD extraheerbaarheid
- [ ] Relevantie aanbeveling

Indicator / aanbeveling	Bron	Jaar	Evidentiegraad
**AANBEVELING:**<br>**De contactgegevens van de contactpersonen, mantelzorgers en als vertegenwoordiger aangewezen personen worden vastgelegd in het dossier.**	**ADEP**<br>**HIQA**<br>**SSMG**<br>**NHS**	**2019**<br>**2018**<br>**?**<br>**2023**	**Geen gradatie**

Uw oordeel:

*

Kies één van de volgende mogelijkheden:

- ◯ 1 (Slecht)
- ◯ 2
- ◯ 3
- ◯ 4
- ◯ 5
- ◯ 6
- ◯ 7
- ◯ 8
- ◯ 9 (Uitstekend)
- ◯ Niet beoordeelbaar

Beoordeling gebaseerd op:

Kies alle voor u geldende mogelijkheden:

- ☐ EMD extraheerbaarheid
- ☐ Relevantie aanbeveling

Indicator / aanbeveling	Bron	Jaar	Evidentiegraad
**AANBEVELING:** **Het EPD dient informatie te bevatten over het zorgteam dat de patiënt opvolgt.**	**NHS**	**2023**	**Geen gradatie**

Uw oordeel:

*

Kies één van de volgende mogelijkheden:

- ◯ 1 (Slecht)
- ◯ 2
- ◯ 3
- ◯ 4
- ◯ 5
- ◯ 6
- ◯ 7
- ◯ 8
- ◯ 9 (Uitstekend)
- ◯ Niet beoordeelbaar

Beoordeling gebaseerd op:

Kies alle voor u geldende mogelijkheden:

- ☐ EMD extraheerbaarheid
- ☐ Relevantie aanbeveling

Indicator / aanbeveling	Bron	Jaar	Evidentiegraad
**AANBEVELING:** **Het EPD dient persoonlijke informatie te bevatten relevant voor de zorgverlener, zijnde: adres patiënt, werkgever, huis en werk telefoonnummer en burgerlijke staat.**	**NCQA**	**2018**	**Geen gradatie**

Uw oordeel:

*

Kies één van de volgende mogelijkheden:

- ◯ 1 (Slecht)
- ◯ 2
- ◯ 3
- ◯ 4
- ◯ 5
- ◯ 6
- ◯ 7
- ◯ 8
- ◯ 9 (Uitstekend)
- ◯ Niet beoordeelbaar

Beoordeling gebaseerd op:

Kies alle voor u geldende mogelijkheden:

- ☐ EMD extraheerbaarheid
- ☐ Relevantie aanbeveling

Top 3 aanbevelingen:

Welke aanbevelingen voor de "patiëntenidentificatie" Vindt u het meest geschikt voor het meten van de kwaliteit voor goed gebruik van het EPD in de huisartsgeneeskunde ?

1.

*

Kies één van de volgende mogelijkheden:

- ◯ In het EPD dient geregistreerd te zijn in welke urgentiedienst (ziekenhuis) de patiënt gekend is.
- ◯ In het EPD dient geregistreerd te zijn of de patiënt gehospitaliseerd is.
- ◯ De contactgegevens van de contactpersonen, mantelzorgers en als vertegenwoordiger aangewezen personen worden vastgelegd in het dossier.
- ◯ Het EPD dient informatie te bevatten over het zorgteam dat de patiënt opvolgt.
- ◯ Het EPD dient persoonlijke informatie te bevatten relevant voor de zorgverlener, zijnde: adres patiënt, werkgever, huis en werk telefoonnummer en burgerlijke staat.

2.

*

Kies één van de volgende mogelijkheden:

- ◯ In het EPD dient geregistreerd te zijn in welke urgentiedienst (ziekenhuis) de patiënt gekend is.
- ◯ In het EPD dient geregistreerd te zijn of de patiënt gehospitaliseerd is.
- ◯ De contactgegevens van de contactpersonen, mantelzorgers en als vertegenwoordiger aangewezen personen worden vastgelegd in het dossier.
- ◯ Het EPD dient informatie te bevatten over het zorgteam dat de patiënt opvolgt.
- ◯ Het EPD dient persoonlijke informatie te bevatten relevant voor de zorgverlener, zijnde: adres patiënt, werkgever, huis en werk telefoonnummer en burgerlijke staat.

3.

*

Kies één van de volgende mogelijkheden:

- ◯ In het EPD dient geregistreerd te zijn in welke urgentiedienst (ziekenhuis) de patiënt gekend is.
- ◯ In het EPD dient geregistreerd te zijn of de patiënt gehospitaliseerd is.
- ◯ De contactgegevens van de contactpersonen, mantelzorgers en als vertegenwoordiger aangewezen personen worden vastgelegd in het dossier.
- ◯ Het EPD dient informatie te bevatten over het zorgteam dat de patiënt opvolgt.
- ◯ Het EPD dient persoonlijke informatie te bevatten relevant voor de zorgverlener, zijnde: adres patiënt, werkgever, huis en werk telefoonnummer en burgerlijke staat.

Indien u nog suggesties heeft voor aanbevelingen die volgens u in bovenstaande lijst ontbreken, mag u deze in onderstaande vakje noteren.

Gelieve steeds duidelijk een aanbeveling en motivatie te noteren voor deze aanbeveling.

Vul uw antwoord hier in:

6. Vaccinatiestatus

In welke mate zijn de volgende aanbevelingen relevant voor het meten van de kwaliteit van het goed gebruik van het EMD in de huisartsgeneeskunde in verband met de **vaccinatiestatus van patiënten**?

Indicator / aanbeveling	Bron	Jaar	Evidentiegraad
**INDICATOR:**<br>**Percentage van de populatie, welke 7 jaar of ouder zijn, die alle basisvaccinaties gekregen hebben. (Overzicht basisvaccinatieschema)**	**CIHI**	**2016**	**Geen gradatie**
**GELINKTE AANBEVELING:**<br>**Een vaccinatiedossier (voor kinderen) is up-to-date of er is een geschikte voorgeschiedenis opgenomen in het medisch dossier (voor volwassenen).**	**NCQA, NHS, Domus Medica, SSMG, HIQA**	**2018, 2023, 2004, ?, 2020**	**Geen gradatie**

Uw oordeel:

*

Kies één van de volgende mogelijkheden:

- ◯ 1 (Slecht)
- ◯ 2
- ◯ 3
- ◯ 4
- ◯ 5
- ◯ 6
- ◯ 7
- ◯ 8
- ◯ 9 (Uitstekend)
- ◯ Niet beoordeelbaar

Beoordeling gebaseerd op:

Kies alle voor u geldende mogelijkheden:

- ☐ EMD extraheerbaarheid
- ☐ Relevantie aanbeveling

Indicator / aanbeveling	Bron	Jaar	Evidentiegraad
**INDICATOR:** **Percentage van de patiëntenpopulatie, 65 jaar en ouder, dat een griepvaccinatie heeft ontvangen.**	**CIHI**	**2016**	**Geen gradatie**
**GELINKTE AANBEVELING:** **Een vaccinatiedossier (voor kinderen) is up-to-date of er is een geschikte voorgeschiedenis opgenomen in het medisch dossier (voor volwassenen).**	**NCQA, NHS, Domus Medica, SSMG, HIQA**	**2018, 2023, 2004, ?, 2020**	**Geen gradatie**

Uw oordeel:

*

Kies één van de volgende mogelijkheden:

- ◯ 1 (Slecht)
- ◯ 2
- ◯ 3
- ◯ 4
- ◯ 5
- ◯ 6
- ◯ 7
- ◯ 8
- ◯ 9 (Uitstekend)
- ◯ Niet beoordeelbaar

Beoordeling gebaseerd op:

Kies alle voor u geldende mogelijkheden:

- ☐ EMD extraheerbaarheid
- ☐ Relevantie aanbeveling

Indicator / aanbeveling	Bron	Jaar	Evidentiegraad
**AANBEVELING:** **Het EPD dient informatie te bevatten dat de patiënt niet gevaccineerd werd.**	**HIQA**	**2018**	**/**

Uw oordeel:

*****

Kies één van de volgende mogelijkheden:

- ◯ 1 (Slecht)
- ◯ 2
- ◯ 3
- ◯ 4
- ◯ 5
- ◯ 6
- ◯ 7
- ◯ 8
- ◯ 9 (Uitstekend)
- ◯ Niet beoordeelbaar

Beoordeling gebaseerd op:

Kies alle voor u geldende mogelijkheden:

- ☐ EMD extraheerbaarheid
- ☐ Relevantie aanbeveling

Indicator / aanbeveling	Bron	Jaar	Evidentiegraad
**AANBEVELING:** **Het EPD dient per vaccinatie de datum van toediening te bevatten.**	**HIQA**	**2018**	**/**

Uw oordeel:

*

Kies één van de volgende mogelijkheden:

- ◯ 1 (Slecht)
- ◯ 2
- ◯ 3
- ◯ 4
- ◯ 5
- ◯ 6
- ◯ 7
- ◯ 8
- ◯ 9 (Uitstekend)
- ◯ Niet beoordeelbaar

Beoordeling gebaseerd op:

Kies alle voor u geldende mogelijkheden:

- ☐ EMD extraheerbaarheid
- ☐ Relevantie aanbeveling

Top 2 aanbevelingen:

Welke aanbevelingen voor de "vaccinatiestatus" vindt u het meest geschikt voor het meten van de kwaliteit voor goed gebruik van het EPD in de huisartsgeneeskunde ?

1.

*

Kies één van de volgende mogelijkheden:

- ◯ Percentage van de populatie, welke 7 jaar of ouder zijn, die alle basisvaccinaties gekregen hebben.
- ◯ Percentage van de patiëntenpopulatie, 65 jaar en ouder, dat een griepvaccinatie heeft ontvangen.
- ◯ Het EPD dient informatie te bevatten dat de patiënt niet gevaccineerd werd.
- ◯ Het EPD dient per vaccinatie de datum van toediening te bevatten.

2.

*

Kies één van de volgende mogelijkheden:

- ◯ Percentage van de populatie, welke 7 jaar of ouder zijn, die alle basisvaccinaties gekregen hebben.
- ◯ Percentage van de patiëntenpopulatie, 65 jaar en ouder, dat een griepvaccinatie heeft ontvangen.
- ◯ Het EPD dient informatie te bevatten dat de patiënt niet gevaccineerd werd.
- ◯ Het EPD dient per vaccinatie de datum van toediening te bevatten.

Indien u nog suggesties heeft voor aanbevelingen die volgens u in bovenstaande lijst ontbreken, mag u deze in onderstaande vakje noteren.

Gelieve steeds duidelijk een aanbeveling en motivatie te noteren voor deze aanbeveling.

Vul uw antwoord hier in:

7. Wil van de patiënt

In welke mate zijn de volgende aanbevelingen relevant voor het meten van de kwaliteit van het goed gebruik van het EMD in de huisartsgeneeskunde in verband met de registratie van **wil van de patiënt** in het EPD?

Indicator / Aanbeveling	Bron	Jaar	Evidentiegraa
**AANBEVELING:** **De verslaglegging van de gesprekken met de patiënt over wensen voor behandelingen en bij het levenseinde wordt in de eerste plaats vastgelegd in SOEP-verslagen in een episode "Gesprek levenseinde/behandelwensen" (ICPC A20, versie 2018)**	**ADEPD, NHS, HASP**	**2019, 2023, 2018**	**Geen gradatie**

Uw oordeel:

*

Kies één van de volgende mogelijkheden:

- ◯ 1 (Slecht)
- ◯ 2
- ◯ 3
- ◯ 4
- ◯ 5
- ◯ 6
- ◯ 7
- ◯ 8
- ◯ 9 (Uitstekend)
- ◯ Niet beoordeelbaar

Beoordeling gebaseerd op:

Kies alle voor u geldende mogelijkheden:

- ☐ EMD extraheerbaarheid
- ☐ Relevantie aanbeveling

Indicator / aanbeveling	Bron	Jaar	Evidentiegraad
**AANBEVELING:** **De uiteindelijke besluiten over wel of niet behandelen in specifieke situaties worden vastgelegd in het dossierdeel "Behandelgrenzen".** **Deze behandelgrenzen zijn: registratie cardiopulmonaire resuscitatie (Reanimatie); opname in het ziekenhuis, opname op intensive care, kunstmatige beademing, toediening van een bloedproduct, overige behandeling (in vrije tekst) of toediening van antibiotica.**	**ADEPD**	**2019**	**Geen gradatie**

Uw oordeel:

*

Kies één van de volgende mogelijkheden:

- ◯ 1 (Slecht)
- ◯ 2
- ◯ 3
- ◯ 4
- ◯ 5
- ◯ 6
- ◯ 7
- ◯ 8
- ◯ 9 (Uitstekend)
- ◯ Niet beoordeelbaar

Beoordeling gebaseerd op:

Kies alle voor u geldende mogelijkheden:

- [ ] EMD extraheerbaarheid
- [ ] Relevantie aanbeveling

Indicator / aanbeveling	Bron	Jaar	Evidentiegraad
**AANBEVELING:** **Schriftelijke wilsverklaringen, bijvoorbeeld een euthanasieverklaring of niet-reanimeerverklaring, die de patiënt aan de huisarts aanbiedt, worden als correspondentie toegevoegd aan het EPD**	**ADEPD**	**2019**	**Geen gradatie**

Uw oordeel:

*

Kies één van de volgende mogelijkheden:

- ◯ 1 (Slecht)
- ◯ 2
- ◯ 3
- ◯ 4
- ◯ 5
- ◯ 6
- ◯ 7
- ◯ 8
- ◯ 9 (Uitstekend)
- ◯ Niet beoordeelbaar

Beoordeling gebaseerd op:

Kies alle voor u geldende mogelijkheden:

- [ ] EMD extraheerbaarheid
- [ ] Relevantie aanbeveling

Indicator / aanbeveling	Bron	Jaar	Evidentiegraad
**AANBEVELING:** **Zijn er doelen voor persoonsgerichte zorg (Individueel Zorgplan = IZP) geregistreerd in het dossier? Bij persoonsgerichte zorg staan de doelen van de patiënt centraal. De patiënt bepaalt samen met zijn zorgverleners de doelen voor de zorg. Deze doelen zijn vaak niet medisch ingestoken, maar hebben er wel raakvlakken mee.** **Bijvoorbeeld: een COPD-patiënt wil nog dagelijks buiten een ommetje kunnen lopen en met de buren babbelen. Een goede instelling van de COPD in combinatie met loop- en ademhalingstraining kunnen het gestelde doel bereikbaar maken. Daarbij moet er afstemming zijn tussen de huisarts (instelling COPD), poh-somatiek (reguliere COPD controles) en een gespecialiseerde fysiotherapeut (loop- en ademhalingstraining). Het IZP maakt inzichtelijk wie wat doet en wie waarvoor verantwoordelijk is.**	**ADEPD**	**2019**	**Geen gradatie**

Uw oordeel:

*****

Kies één van de volgende mogelijkheden:

◯ 1 (Slecht)
◯ 2
◯ 3
◯ 4
◯ 5
◯ 6
◯ 7
◯ 8
◯ 9 (Uitstekend)
◯ Niet beoordeelbaar

Beoordeling gebaseerd op:

Kies alle voor u geldende mogelijkheden:

☐ EMD extraheerbaarheid
☐ Relevantie aanbeveling

Indicator / aanbeveling	Bron	Jaar	Evidentiegraad
**AANBEVELING:**<br>**Het EPD dient informatie te bevatten betreffende de screeningstrajecten van toepassing voor iedere patiënt of de wensen van de patiënt betreffende deze screening.**<br>**In België is aanwezig als bevolkingsonderzoek: borstkanker, baarmoederhalskanker, dikke darmkanker, aangeboren aandoeningen. (Soorten bevolkingsonderzoeken | Bevolkingsonderzoek)**<br>**Opmerking: hier gaat het over accepteren/afwijzen van zorgaanpakken en niet over aanwezigheid van individuele planningen (zoals de aanbeveling in topic 4).**	**ADEPD**	**2019**	**Geen gradatie**

Uw oordeel:

*

Kies één van de volgende mogelijkheden:

- ◯ 1 (Slecht)
- ◯ 2
- ◯ 3
- ◯ 4
- ◯ 5
- ◯ 6
- ◯ 7
- ◯ 8
- ◯ 9 (Uitstekend)
- ◯ Niet beoordeelbaar

Beoordeling gebaseerd op:

Kies alle voor u geldende mogelijkheden:

- [ ] EMD extraheerbaarheid
- [ ] Relevantie aanbeveling

Top 3 aanbevelingen:

Welke aanbevelingen voor de "wil van de patiënt" Vindt u het meest geschikt voor het meten van de kwaliteit voor goed gebruik van het EPD in de huisartsgeneeskunde ?

1.

*

Kies één van de volgende mogelijkheden:

- ◯ De verslaglegging van de gesprekken met de patiënt over wensen voor behandelingen en bij het levenseinde wordt in de eerste plaats vastgelegd in SOEP-verslagen in een episode "Gesprek levenseinde/behandelwensen" (ICPC A20, versie 2018)
- ◯ De uiteindelijke besluiten over wel of niet behandelen in specifieke situaties worden vastgelegd in het dossierdeel "Behandelgrenzen".
- ◯ Schriftelijke wilsverklaringen, bijvoorbeeld een euthanasieverklaring of niet-reanimeerverklaring, die de patiënt aan de huisarts aanbiedt, worden als correspondentie toegevoegd aan het EPD
- ◯ Zijn er doelen voor persoonsgerichte zorg (Individueel Zorgplan = IZP) geregistreerd in het dossier?
- ◯ Het EPD dient informatie te bevatten betreffende de screeningstrajecten van toepassing voor iedere patiënt of de wensen van de patiënt betreffende deze screening.

2.

*

Kies één van de volgende mogelijkheden:

- ◯ De verslaglegging van de gesprekken met de patiënt over wensen voor behandelingen en bij het levenseinde wordt in de eerste plaats vastgelegd in SOEP-verslagen in een episode "Gesprek levenseinde/behandelwensen" (ICPC A20, versie 2018)
- ◯ De uiteindelijke besluiten over wel of niet behandelen in specifieke situaties worden vastgelegd in het dossierdeel "Behandelgrenzen".
- ◯ Schriftelijke wilsverklaringen, bijvoorbeeld een euthanasieverklaring of niet-reanimeerverklaring, die de patiënt aan de huisarts aanbiedt, worden als correspondentie toegevoegd aan het EPD
- ◯ Zijn er doelen voor persoonsgerichte zorg (Individueel Zorgplan = IZP) geregistreerd in het dossier?
- ◯ Het EPD dient informatie te bevatten betreffende de screeningstrajecten van toepassing voor iedere patiënt of de wensen van de patiënt betreffende deze screening.

3.
*

Kies één van de volgende mogelijkheden:

◯ De verslaglegging van de gesprekken met de patiënt over wensen voor behandelingen en bij het levenseinde wordt in de eerste plaats vastgelegd in SOEP-verslagen in een episode "Gesprek levenseinde/behandelwensen" (ICPC A20, versie 2018)

◯ De uiteindelijke besluiten over wel of niet behandelen in specifieke situaties worden vastgelegd in het dossierdeel "Behandelgrenzen".

◯ Schriftelijke wilsverklaringen, bijvoorbeeld een euthanasieverklaring of niet-reanimeerverklaring, die de patiënt aan de huisarts aanbiedt, worden als correspondentie toegevoegd aan het EPD

◯ Zijn er doelen voor persoonsgerichte zorg (Individueel Zorgplan = IZP) geregistreerd in het dossier?

◯ Het EPD dient informatie te bevatten betreffende de screeningstrajecten van toepassing voor iedere patiënt of de wensen van de patiënt betreffende deze screening.

Indien u nog suggesties heeft voor aanbevelingen die volgens u in bovenstaande lijst ontbreken, mag u deze in onderstaande vakje noteren.

Gelieve steeds duidelijk een aanbeveling en motivatie te noteren voor deze aanbeveling.

Vul uw antwoord hier in:

Het einde

Bedankt voor uw deelname aan het eerste deel van het onderzoek voor het opstellen van een set indicatoren betreffende het correct gebruik van het elektronisch patiëntendossier bij huisartsen. Hopelijk mogen wij u op 5 november 2024 om 20u00 verwachten voor het panelgesprek. U krijgt hiervoor nog een herinneringsmail.

Datum	Wat?	Hoe?	Duur?
30 september 2024 23u59	Deadline invullen vragenlijst	Online enquete	30 min
5 november 2024 20u00	Panelgesprek	Online	120 min
30 november 2024 23u59	Deadline nalezen definitieve indicatorenlijst	Email	15 min

Indien u nog suggesties heeft voor aanbevelingen die volgens u in bovenstaande lijst ontbreken, mag u deze in onderstaande vakje noteren.

Gelieve steeds duidelijk een aanbeveling en motivatie te noteren voor deze aanbeveling.

Vul uw antwoord hier in:

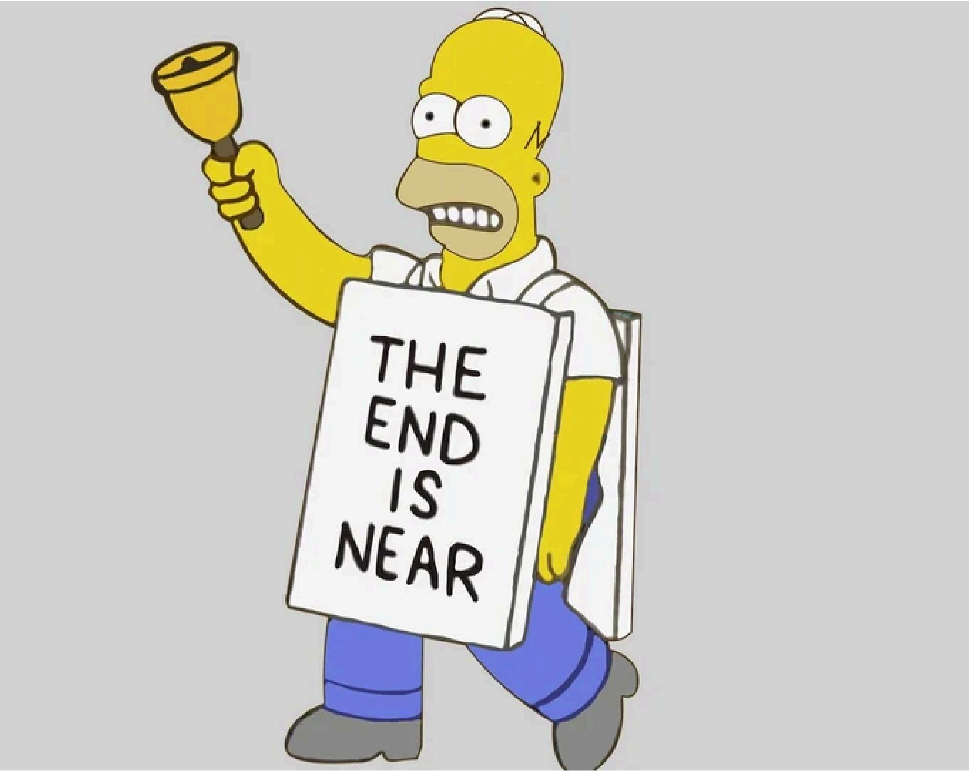

16-11-2024 – 17:01
Verzend uw enquête.
Bedankt voor uw deelname aan deze enquête.
