# Supplementary material for: Development of Quality Indicators for the Correct Use of Electronic Medical Records in Primary Care: Modified Delphi Study
Source: JMIR Med Inform. 2026 Jan 19;14:e80057. doi: 10.2196/80057 (PMC12865340; doi:10.2196/80057)
Supplement: Multimedia Appendix 3 [file medinform_v14i1e80057_app3.pdf]

Développement d'indicateurs de qualité pour le bon usage du dossier électronique du patient en médecine générale

Madame,
Monsieur,

Tout d'abord, nous tenons à vous remercier d'avoir accepté de participer à notre panel d'experts. L'étude dans laquelle le panel d'experts joue un rôle important est menée par le Centre académique de médecine générale de la KU Leuven et vise à développer une sélection d'indicateurs de qualité pour le bon usage du dossier électronique du patient en médecine générale. Une exigence spécifique pour la sélection des indicateurs concerne l'extraction (ou la possibilité d'extraction) des données médicales du Dossier Médical Informatisé (DMI) (ou directement à partir du DMI). L'objectif final est de fournir un retour d'information automatique aux médecins généralistes sur la qualité des dossiers médicaux de leurs patients sur la base des indicateurs de qualité. Nous souhaitons ainsi obtenir une amélioration de la qualité des soins.

En raison de vos connaissances et de votre expertise, vous avez été invités à participer à la sélection du développement d'indicateurs de qualité pour le bon usage du dossier électronique du patient en médecine générale. Comme indiqué dans l'invitation, nous utilisons la méthode Delphi modifiée Rand pour la sélection des indicateurs de qualité dans cette étude. Il s'agit d'une méthode de consensus en trois étapes. Dans la première étape, nous vous demandons de noter une liste de recommandations en fonction de leur importance, de leur utilité et de leur pertinence pour les soins primaires. Avec votre aide, l'objectif est de rédiger un ensemble de recommandations courtes et pertinentes qui, après avoir suivi toute la procédure, seront traduites en un set de base des indicateurs de qualité du DMI. Il faut compter environ 30 minutes pour remplir le questionnaire. Nous aimerions recevoir votre questionnaire complété avant **30 septembre 2024 23h59**.

Dans un deuxième temps, les résultats des questionnaires remplis seront analysés. Au cours d'un réunion de consensus, nous tenterons de parvenir à un consensus sur l'acceptation, le rejet ou la reformulation des indicateurs potentiels. Cet entretien, dont la date sera communiquée ultérieurement, durera environ deux heures.

Dans la troisième et dernière étape, l'objectif est de soumettre la liste finale des indicateurs par écrit à tous les experts du panel afin d'obtenir leur approbation finale. Cette étape ne prendra pas plus de 15 minutes et la date sera également annoncée ultérieurement.

Si vous avez des questions ou des commentaires, n'hésitez pas à nous contacter par e-mail (rico.paridaens@outlook.com).

Je vous prie d'agréer, Madame, Monsieur, l'expression de mes salutations distinguées,

Le groupe de pilotage est composé de:
Rico Paridaens, médecin généraliste, KU Leuven
Professeur Bert Vaes, médecin généraliste, KU Leuven
Steve Van den Bulck, médecin généraliste, KU Leuven

Il y a 148 questions dans ce questionnaire.

Informations générales sur l'étude

Recherche sur les objectifs généraux

L'étude dans laquelle le panel d'experts joue un rôle important est menée par le "Academisch Centrum voor Huisartsgeneeskunde" de la KU Leuven" et vise à développer une sélection d'indicateurs de qualité pour une bonne utilisation du dossier médical informatisé de médecine générale. Une exigence spécifique pour la sélection des indicateurs concerne l’extraction (ou la possibilité d’extraction) des données médicales du Dossier Médical Informatisé (DMI) (ou directement à partir du DMI). L'objectif final est de fournir un retour d'information automatisé aux médecins généralistes sur la qualité de l'enregistrement des données dans les dossiers medicaux sur la base des indicateurs de qualité. De cette manière, nous essayons d'obtenir une amélioration de la qualité des soins.

Informations sur le traitement de vos données personnelles

Dans le cadre de votre participation à cette étude, des données personnelles vous concernant seront collectées et traitées. Ce traitement sera effectué conformément au règlement général sur la protection des données (AVG / RGPD). Les catégories de données personnelles suivantes seront traitées dans le cadre de cette étude: nom, âge, sexe, fonction, lieu de travail (hôpital/pratique et service) pour le rapport de consensus, enregistrement audio pendant la réunion de consensus pour écrire le rapport de consensus.

Utilisation de vos données personnelles

Seules les données personnelles nécessaires aux fins de cette étude seront collectées et traitées. Vos données seront pseudonymisées dans le cadre de cette étude. Cela signifie que les données permettant de vous identifier, telles que le nom, l'âge, le sexe, la fonction, le lieu de travail (hôpital/pratique et service), seront séparées des autres données de recherche et remplacées par un code unique et aléatoire. De cette manière, il n'est plus possible de voir immédiatement quelles données proviennent de quelle personne spécifique. Seul le chercheur peut utiliser le code unique pour relier les données à une personne spécifique. Toutefois, cela ne se fera que dans des cas exceptionnels, par exemple si vous invoquez votre droit de consulter ou de rectifier vos données. Pour la qualité de l'étude, il est important que nous soyons autorisés à mentionner votre nom, votre âge, votre sexe, votre fonction, votre lieu de travail (hôpital/pratique et service) en tant que participant à l'étude dans les résultats de cette étude, tels que les publications. Vous ne serez pas identifié. Toutefois, les résultats ne feront jamais référence directement à un individu dans les résultats de cette étude et seront toujours mentionnés en tant que conclusion générale.

L'intérêt public sera utilisé comme base juridique pour le traitement de vos données (article 6e du RGPD). Cela signifie que l'étude conduira à une augmentation des connaissances et de la compréhension qui profitera à la société (directement ou indirectement). L'arrêt de la participation à l'étude signifie que les données collectées précédemment peuvent encore être légalement impliquées dans l'étude et ne doivent pas être supprimées par la KU Leuven.

Vos données seront conservées par les chercheurs pendant 10 ans après la fin de l'étude dans un lieu de stockage sécurisé de la KU Leuven.

Vos droits

Vous avez toujours le droit de demander plus d'informations sur l'utilisation de vos données. En outre, vous pouvez exercer le droit de regard et le droit de correction (rectification) de vos données dans la mesure où ces droits ne rendent pas impossible ou n'entravent pas sérieusement la réalisation des objectifs de la recherche.

Si vous souhaitez invoquer l'un de ces droits, veuillez contacter les chercheurs en utilisant les coordonnées figurant au début de cette enquête.

Réutilisation de vos données

Il est possible que vos données pseudonymisées soient réutilisées à des fins de recherche scientifique par: des chercheurs et/ou des partenaires académiques collaborant avec la KU Leuven ou recevant des données de la KU Leuven (y compris - sous certaines conditions - un étudiant en master ou en doctorat),

La mise à disposition des données est essentielle pour valider les résultats de la recherche et faire progresser les connaissances scientifiques.

En cas de réutilisation de vos données, les dispositions contractuelles nécessaires seront toujours prises pour protéger les données à caractère personnel et déterminer les responsabilités des parties conformément à la législation applicable.

Dans le cadre de la réutilisation, vos données à caractère personnel peuvent être transférées en dehors de l'Espace économique européen, à condition que des mesures appropriées aient été prises pour protéger vos données à caractère personnel conformément à la législation applicable. En particulier, ces données ne seront en principe transférées que sous

forme pseudonymisée et toutes les parties impliquées dans la recherche seront tenues de respecter la confidentialité des données à caractère personnel.

Des informations transparentes seront fournies sur toute réutilisation de vos données. Ces informations seront envoyées par courrier électronique.

Toute réutilisation sera toujours conforme à la législation applicable et à la politique de la KU Leuven en la matière. Cette politique signifie, entre autres, qu'un organisme indépendant contrôlera la protection des données à caractère personnel et de vos droits. Veuillez noter que vos données pseudonymisées peuvent également être mises à disposition sur certaines plateformes de données à des fins de recherche scientifique, avec un accès strictement sécurisé et contrôlé. Cela peut se produire, par exemple, dans le cadre de la publication de résultats. La plateforme de données fournit une politique d'accès et s'engage à réglementer l'accès aux données conformément à la législation applicable.

Coordonnées

La KU Leuven agit en tant que contrôleur des données dans le cadre de cette recherche. Plus précisément, seuls les chercheurs Rico Paridaens, Bert Vaes et Steve Van den Bulck auront accès à vos données personnelles. En cas de questions spécifiques concernant cette recherche, y compris le traitement de vos données personnelles, vous pouvez les contacter.

Pour toute autre question ou préoccupation concernant le traitement de vos données personnelles, veuillez contacter le délégué à la protection des données pour la recherche scientifique à la KU Leuven (dpo@kuleuven.be). Veuillez préciser de quelle recherche il s'agit en mentionnant le titre et le nom des chercheurs.

Si, après avoir contacté le délégué à la protection des données, vous souhaitez déposer une plainte concernant le traitement de vos informations, vous pouvez contacter l'Autorité belge de protection des données (www.gegevensbeschermingsautoriteit.be).

Informations spécifiques sur l'étude

De la recommandation à l'indicateur

Pour établir la liste des recommandations figurant dans le présent document, nous nous sommes appuyés sur des lignes directrices nationales et internationales (voir la citation des sources). Nous avons commencé par établir une liste reprenant toutes les recommandations de du dossier médical informatisé en médecine générale de toutes les sources consultées. Cependant, il serait impossible de travailler avec une liste aussi complète pour sélectionner des indicateurs pertinents. C'est pourquoi, dans une étape suivante, nous avons sélectionné les recommandations utilisables en soins primaires et automatiquement extractibles de l'DMI. Il s'agit en effet de deux conditions préalables importantes dans le contexte de notre étude. Nous avons également vérifié si les recommandations étaient "SMART", c'est-à-dire **S**pecific (spécifiques), **M**easurable (mesurables), **A**chievable (réalistes), **R**elevant (pertinentes) et **T**ime-boud (limitées dans le temps).

À ce stade, votre objectif est de noter toutes les recommandations dans la mesure où vous considérez que les recommandations spécifiques sont importantes et peuvent être extraites de le DMI pour mesurer la qualité des dossiers médicaux électroniques dans le domaine des soins primaires. Pour plus d'explications, voir les instructions ci-dessous.

Instructions pour remplir le formulaire

Tout d'abord, veuillez remplir le consentement éclairé ci-joint. Ensuite, vous trouverez dans ce questionnaire de 50 indicateurs / recommandations, réparties dans les catégories suivantes:

1. exhaustivité et adéquation de la liste des problèmes (16 indicateurs/recommandations);
2. enregistrement structuré dans le DMI (5 indicateurs/recommandations);
3. exhaustivité et actualisation de la liste des médicaments (5 indicateurs/recommandations);
4. facteurs de risque et surveillance des médicaments (10 indicateurs/recommandations);
5. identification du patient / informations de contact (5 indicateurs/recommandations);
6. statut vaccinal (4 indicateurs/recommandations); et
7. volontés du patient (5 indicateurs/recommandations).

Nous vous demandons d'évaluer chaque recommandation en fonction de la mesure dans laquelle elle peut être extraite du DMI et est pertinente pour mesurer la qualité des dossiers de santé électroniques dans les soins primaires, en utilisant une échelle de Likert en 9 points. Il s'agit d'une échelle de 1 à 9, le score le plus bas étant 1 (mauvaise mesure de la qualité des soins) et le score le plus élevé étant 9 (excellente mesure de la qualité des soins).

Pour déterminer si une recommandation peut servir à mesurer la qualité des soins, vous pouvez utiliser les critères suivants :

- La recommandation est pertinente dans le processus de soins primaires.
- La recommandation améliorée la santé du patient.
- La recommandation améliore la qualité de vie du patient.
- La recommandation améliore l'efficacité des soins pour le patient.
- La recommandation peut être traduite en un indicateur qui peut être extrait automatiquement de le DMI.

En plus d'un score selon l'échelle de Likert en 9 points, après avoir évalué toutes les recommandations d'une catégorie, nous vous demandons également de compiler les cinq meilleures recommandations par catégorie en fonction de leur pertinence pour mesurer la qualité des soins. Pour chaque catégorie, nous vous offrons la possibilité d'ajouter des éléments à ce top 5.

Nous vous offrons la possibilité de faire vos propres commentaires, de compléter les recommandations ou de rédiger vos propres recommandations. Pour ce faire, veuillez utiliser le question à la fin du questionnaire.

Sources utilisées

Les sources consultées pour établir la liste des recommandations sont énumérées ci-dessous. Les abréviations utilisées dans le questionnaire (avant), l'année de publication ou de dernière mise à jour et le pays d'origine sont également indiqués.

- Domus Medica: Verdonck P, Strobbe J, Steenackers J et al. Het elektronisch medisch dossier. Huisarts Nu maart 2004; 33(2).
- SSMG: Société Scientifique de Médecine Générale (SSMG). Organisation de la pratique. [cited 2024 Jan 27]; Available from: https://www.ssmg.be/organisation-pratique/
- NCQA: National Committee for Quality Assurance (NCQA). Guidelines for Medical Record Documentation. 2018 [cited 2024 Jan 27]; Available from: https://www.ncqa.org/wp-content/uploads/2018/07/20180110_Guidelines_Medical_Record_Documentation.pdf

- ADEPD: Duineveld B, Kole HM, Van Werven H. NHG-Richtlijn Adequate dossiervorming met het elektronisch patiëntdossier (ADEPD). 2019 [cited 2024 Jan 27]; Available from: https://www.nhg.org/praktijkvoering/informatisering/richtlijn-adequate-dossiervorming-epd/
- HASP: Federatie Medisch Specialisten (FMS), Nederlands Huisartsen Genootschap (NHG). Richtlijn Informatie-uitwisseling tussen huisarts en medisch specialist (HASP. 2017 [cited 2024 Jan 27]; Available from: https://www.nhg.org/praktijkvoering/gegevensuitwisseling/gegevensuitwisseling-huisarts-specialist-hasp/
- NHS: National Health Service (NHS). Summary Care Records. Version 1.3, 19 April 2023. [cited 2024 Jan 27]; Available from: https://www.england.nhs.uk/long-read/summary-care-records-scr/
- HIQA: Health Information and Quality Authority. Recommendations on the implementation of a national electronic patient summary in Ireland. 2020 [cited 2024 Jan 27]; Available from: https://www.hiqa.ie/reports-and-publications/health-information/recommendations-implementation-national-electronic
- Hiddema-van der Wal: Hiddema-van der Wal A, van der Werf GTh, Meyboom-de Jong B. Welke ICPC-codes willen huisartsen automatisch laten toevoegen aan de probleemlijst? Huisarts en Wetenschap 46(10) september 2003. Page 539-543
- EPD-scan-h: Lea Jabaaij, Robert Verheij, Khing Njoo, Henk van den Hoogen, Waling Tiersma, Herman Levelink. Het meten van de kwaliteit van de registratie in elektronische patiënten dossiers van huisartsen met de EPD-scan-h (EPD-scan-h). ISBN 978-90-6905-896-2. 2008
- Hamade et al: Hamade N, Terry A, Malvankar-Mehta M. Interventions to improve the use of EMRs in primary health care: a systematic review and meta-analysis. BMJ Health Care Inform. 2019 May;26(1):e000023.
- De Lusignan et al: De Lusignan S. Does Feedback Improve the Quality of Computerized Medical Records in Primary Care? Journal of the American Medical Informatics Association. 2002 Jul 1;9(4):395–401.
- CIHI: Ottawa - Ontario : Canadian Institute for Health Information. Pan-Canadian primary health care indicator - update report. 2016. Canada. ISBN: 978-1-77109-146-6

Informed Consent

Titre:
Développement d'indicateurs de qualité pour une bonne utilisation du dossier médical informatisé en médecine générale

Nom + coordonnées du promoteur et du/des chercheur(s):

- **Chercheur: Rico Paridaens, rico.paridaens@outlook.com, 0470 83 39 09**
- **Promoteur: Bert Vaes, bert.vaes@kuleuven.be, 0474 33 05 13, Departement Maatschappelijke Gezondheidszorg en Eerstelijnszorg, Academisch Centrum voor Huisartsgeneeskunde**

Objectif et méthodologie de l'étude:
INTEGO vise à établir des indicateurs de qualité pour les principaux états pathologiques en médecine générale et pour l'utilisation du dossier médical électronique par les médecins généralistes par les procédures RAND-Modiphied Delphi. Il s'agit d'une procédure dans laquelle chaque participant passe systématiquement par plusieurs étapes pour finalement obtenir un rapport de consensus avec des recommandations qui sont soutenues par tous les participants. Pour cette étude, nous déterminerons des indicateurs de qualité pour: indicateurs de qualité pour une bonne utilisation du dossier médical informatisé en médecine générale.

Au cours de l'étude, vous passerez par les étapes suivantes:

1. **Questionnaire électronique : Il vous sera demandé de noter une liste d'indicateurs de qualité potentiels / de recommandations sur leur capacité à mesurer la qualité du sujet l'enlément concerné'ée utilisant une échelle de Likert de 1 (= score le plus bas) à 9 (= score le plus élevé). Vous recevrez un rapport de feedback sur le premier tour avec votre score personnel, le score médian de tous les participants et, sur cette base, le potentiel de l'indicateur de qualité/recommandation.**
2. **Discussion en face à face: Les recommandations modérées (résultat douteux) seront discutées ainsi que les indicateurs nouvellement introduits. Les indicateurs forts et faibles ne sont discutés que si des commentaires ont été formulés à leur sujet. Sur la base des conclusions de la réunion en face à face, un ensemble de recommandations est élaboré et soumis pour une troisième et dernière évaluation à tous les membres du panel pour approbation.**

Durée de l'étude: +/- 30 min

Je comprends et je consens à:

- **Je comprends ce que l'on attend de moi au cours de cette étude.**

- **Je sais que je participerai à des essais ou des tests ultérieurs: Enquête électronique et réunion de consensus**
- **Je sais que ma participation peut comporter des risques ou des inconvénients: Situations conflictuelles possibles lors des discussions de réunion de consensus.**
- **Cette recherche peut être bénéfique pour moi ou pour d'autres personnes de la manière suivante: Ces indicateurs de qualité peuvent être utilisés pour fournir un retour d'information aux médecins généralistes en Belgique et dans d'autres pays sur la qualité des soins prodigués aux patients.**
- **Ma participation contribue à la recherche scientifique. Je sais que je ne recevrai aucune autre récompense ou compensation pour ma participation.**
- **Je comprends que ma participation à cette étude est volontaire. J'ai le droit de mettre fin à ma participation à tout moment. Je n'ai pas besoin de donner de raison pour cela et je sais qu'aucun inconvénient ne peut en résulter pour moi.**
- **Je sais que des enregistrements peuvent être faits de moi dans le cadre de cette étude : enregistrement audio pendant la discussion en face à face du panel pour l'élaboration du rapport de consensus.**
- **Mes données personnelles seront traitées conformément au règlement général sur la protection des données (AVG/GDPR). Ce faisant, seules les données strictement nécessaires pour atteindre les objectifs de la recherche seront traitées. Tout au long de l'étude, mes données resteront confidentielles à tout moment. Les chercheurs prendront des mesures pour protéger ma vie privée. Par exemple, mes données personnelles seront pseudonymisées, ce qui signifie que mes données ne pourront plus être reliées à moi sans l'utilisation d'informations supplémentaires accessibles uniquement aux chercheurs. Je comprends que mes données pseudonymisées peuvent être réutilisées pour d'autres recherches scientifiques et éventuellement dans le contexte de l'enseignement et des conférences universitaires. De plus amples informations sur le traitement de mes données personnelles figurent dans la lettre d'information ci-jointe.**

- **Je souhaite être tenu(e) informé(e) des résultats de cette recherche. Le chercheur peut me contacter à cette fin à l'adresse électronique suivante:**

Veuillez écrire votre réponse ici :

- **Bien que cette étude n'implique pas de dépistage diagnostique, il existe un faible risque que les chercheurs rencontrent par hasard des résultats de recherche imprévus dont ils jugent nécessaire de m'informer (par exemple, des indications possibles de problèmes médicaux ou psychologiques). Dans ce cas, ils peuvent me contacter à l'adresse électronique ci-dessus pour m'en informer. Si je ne souhaite pas en être informé(e), je coche la case ci-dessous.**

Veuillez choisir toutes les réponses qui conviennent :

☐ Je ne souhaite pas être informé(e) des résultats imprévus de la recherche.

- **Pour toute autre question concernant l'étude, je sais qu'après ma participation, je peux contacter:**
  Rico Paridaens (rico.paridaens@outlook.com)
- **Cette étude a été examinée et approuvée par le Comité d'éthique sociétale (SMEC) de la KU Leuven (G-2024-8020, veuillez citer ce numéro dans toute communication relative à l'étude). Pour toute plainte ou autre préoccupation concernant les aspects éthiques de cette étude, je peux contacter le SMEC : smec@kuleuven.be**
- **Je sais que je peux contacter les personnes suivantes si j'éprouve une gêne ou des difficultés à la suite de l'étude en raison des questions soulevées dans l'étude:**
  Rico Paridaens (rico.paridaens@outlook.com)

J'ai lu et compris les informations ci-dessus et j'ai obtenu des réponses à toutes mes questions concernant cette étude. Je consens à participer.

*****

Veuillez sélectionner une seule des propositions suivantes :

◯ Accord

◯ Pas d'accord

Informations personnelles

Nom et prénom :
(Cette information ne sera utilisée que pour l'envoi des résultats du premier cycle Delphi et pour l'invitation à la réunion de consensus). *

Veuillez écrire votre réponse ici :

Adresse électronique :
(Cette information ne sera utilisée que pour l'envoi des résultats du premier cycle Delphi et pour l'invitation à la réunion de consensus). *

Veuillez écrire votre réponse ici :

Âge: *

Veuillez écrire votre réponse ici :

Genre: *

Au besoin, veuillez préciser le champ ‘Autre :’.
Veuillez sélectionner une seule des propositions suivantes :

- ◯ Homme
- ◯ Femme
- ◯ Autre

Lieu de travail:
(Si hôpital : préciser)
*

Veuillez sélectionner une seule des propositions suivantes :

- ◯ Hôpital
- ◯ Pratique générale: pratique en solo/duo
- ◯ Pratique générale: cabinet de groupe

Faites le commentaire de votre choix ici :

Poste à le lieu de travail: *

Veuillez sélectionner une seule des propositions suivantes :

- ◯ Médecin généraliste
- ◯ Autre

Affiliation: *

Veuillez sélectionner une seule des propositions suivantes :

- ◯
- ◯
- ◯
- ◯
- ◯
- ◯
- ◯
- ◯
- ◯
- ◯
- ◯
- ◯ Autre

Quel DMI utilisez-vous ? *

Veuillez sélectionner une seule des propositions suivantes :

- ◯ CareConnect
- ◯ Health One
- ◯ Medispring
- ◯ Daktari
- ◯ Autre ____________

1. Exhaustivité et adéquation de la liste des problèmes

Dans quelle mesure les recommandations suivantes sont-elles pertinentes pour mesurer la qualité d'une bonne utilisation du DMI en médecine générale en ce qui concerne **l'exhaustivité et de l'adéquation de la liste des problèmes** des patients?

Indicateur / recommandation	Source	Année	Niveau de preuve
**INDICATEUR:**<br>**Quel est le pourcentage de sous-contacts dans le journal qui sont connecté avec une épisode de soin/santé?**	**EPD-scan-h**	**2009**	**Pas de gradation**
**RECOMMANDATION LIÉE:**<br>**Liste des épisodes : le DMI présente séquentiellement les épisodes ouverts avec indicateur, les épisodes fermés avec indicateur et les épisodes ouverts sans indicateur.**	**ADEPD, HIQA**	**2019, 2020**	**Pas de gradation**

Votre évaluation:

*

Veuillez sélectionner une seule des propositions suivantes :

- ◯ 1 (Médiocre)
- ◯ 2
- ◯ 3
- ◯ 4
- ◯ 5
- ◯ 6
- ◯ 7
- ◯ 8
- ◯ 9 (Excellent)
- ◯ Non évaluable

Evaluation basée sur:

Veuillez choisir toutes les réponses qui conviennent :

- ☐ L’extractibilité de le DMI
- ☐ Recommandation sur la pertinence

Indicateur / Recommandation	Source	Année	Niveau de preuve
**INDICATEUR:** Quel est le pourcentage d'élément de santé/soins avec une attention particulière qui sont effectivement étiquetés "valeur d'attention particulière"?	EPD-scan-h	2009	Pas de gradation

Informations complémentaires : Les problèmes ayant une valeur d'attention particulière sont automatiquement placés en haut de la liste des problèmes afin d'être très visibles dans le DMI. Les éléments de soins dignes d'un statut de problème sont des éléments de soins avec un code ICPC qui peuvent être placés automatiquement sur la liste des problèmes parce qu'ils sont importants pour le prestataire de soins de santé. Ces éléments de soins sont les suivants:

A12	Allergie/réaction allergique negative	N88	Epilepsie
A70	tuberculose [excl. R70]	N89	Migraine
A79	Cancer NCA	P15	Alcoolisme chronique
A85	Effet sec. subst. médicinale	P18	Usage abusif de médicamen
A90	Anom. congénitale NCA/multiple	P21	S/P du comportement de l'enfant
B72	Maladie de Hodgkin/lymphome	P70	Démence
B73	Leucémie	P71	Autre psychose organique
B74	Autre cancer du sang	P72	Schizophrénie
B76	Rupture traumat. de la rate	P73	Psychose affective
B78	Anémie hémolytique héréditaire	P74	Trouble anxieux/état anxie
B79	Autre anom. congénitale sang/ lymph/rate	P76	Dépression

B81	Anémie carence vit B12/ac. folique	P77	Suicide/tentative de suicide
B83	Purpura/défaut de coagulation	R70	tuberculose tr. resp. [excl. gegen.A70]
D74	Cancer de l'estomac	R78	Bronchite aiguë, bronchiolit (> 2x/ans)
D75	Cancer du colon/du rectum	R82	Pleurésie, épanchement pleural [excl. tbc R70]
D76	Cancer du pancréas	R84	Cancer des bronches, du poumon
D77	Autre cancer digestif/NCA	R85	Autre cancer respiratoire
D78	Tumeur bénigne/indét. du syst. dig.	R89	Anom. congénitale du syst. resp.
D81	Anom. congénitale du syst. digestif	R91	Bronchite chronique / bronchiëctasie
D85	Ulcère duodénal	R95	Mal. pulmonaire chronique obstructive / BPCO
D86	Autre ulcère peptique	R96	Asthme
D92	Maladie diverticulaire	R97	Rhinite allergique
D93	Syndrome du colon irritable	S77	Cancer de la peau
D94	Entérite chronique/colite ulcéreuse	S87	Dermatite atopique/eczéma
D97	Maladie du foie NCA	S91	Psoriasis
D98	Cholécystite/cholélithiase	T71	Cancer de la thyroïde
F81	Autre anom. congénitale de l’œil	T72	Tumeur bénigne de la thyro
F92	Cataracte	T80	Anom. congénit. endoc/ métab./nutrit.
F93	Glaucome	T81	Goitre [excl. T85]
F94	Cécité	T85	Hyperthyroïdie/thyréotoxic
H83	Otosclérose	T86	Hypothyroïdie/myxœdème

**H84**	**Presbyacousie**	**T90**	**Diabète non insulino-dépendant**
**K73**	**Anom. congénitale cardio-vasculaire**	**T92**	**Goutte**
**K74**	**Cardiopathie ischémique avec angor**	**T93**	**Trouble du métabolisme des lipides**
**K75**	**Infarctus myocardique aigu**	**U71**	**Cystite/autre infection urinaire (>2x/ans)**
**K76**	**Cardiopathie ischémique sans angor**	**U75**	**Cancer du rein**
**K77**	**Décompensation cardiaque**	**U76**	**Cancer de la vessie**
**K78**	**Fibrillation auriculaire/flutter**	**U77**	**Autre cancer urinaire**
**K79**	**Tachycardie paroxystique**	**U85**	**Anom. congénitale du tract[illegible] urinaire**
**K83**	**Valvulopathie NCA**	**U88**	**Glomérulonéph./syndr. néphrotique**
**K86**	**Hypertension non compliquée**	**U95**	**Lithiase urinaire**
**K87**	**Hypertension avec complication**	**W13**	**Stérilisation chez la femme**
**K89**	**Ischémie cérébrale transitoire**	**W72**	**Tumeur maligne avec grossesse**
**K90**	**Accident vasculaire cérébral**	**W76**	**Anom. congénitale et grossesse**
**K91**	**Maladie cérébrovasculaire**	**W80**	**Grossesse ectopique**
**K92**	**Athéroscl./mal. vasculaire périphér**	**W82**	**Avortement spontané**
**K93**	**Embolie pulmonaire**	**W92**	**Acc. complique, enfant viva[illegible]**
**K94**	**Phlébite et thrombophlébite**	**W93**	**Acc. complique, enfant mor[illegible]**
**L75**	**Fracture du fémur**	**X74**	**Mal. inflammatoire pelvienn[illegible] femme**

**L82**	**Anom. congénitale ostéo-articulaire**	**X75**	**Cancer du col de l'utérus**
**L88**	**Polyarthrite rhumatoïde séropositive**	**X76**	**Cancer du sein chez la femn**
**L89**	**Coxarthrose**	**X77**	**Autre cancer génital chez la femme**
**L95**	**Ostéoporose**	**X78**	**Fibrome utérin**
**N70**	**Poliomyélite**	**X83**	**Anom. génitale congénitale femme**
**N71**	**Méningite/encéphalite NCA**	**X87**	**Prolapsus utero-vaginal**
**N72**	**Tétanos**	**Y77**	**Cancer de la prostate**
**N74**	**Cancer du syst. neurologique**	**Y78**	**Autre cancer génital chez l'homme**
**N75**	**Tumeur bénigne neurologique**	**Y82**	**Hypospadias**
**N85**	**Anom. congénitale neurologique**	**Y83**	**Ectopie testiculaire**
**N86**	**Sclérose en plaque**	**Y84**	**Autre anom. congénitale homme**
**N87**	**Syndrome parkinsonien**	**Y85**	**Hypertrophie bénigne de la prostate**

Source: A Hiddema-van der Wal, GTh van der Werf, B Meyboom-de Jong, "Welke ICPC-codes willen huisartsen automatisch laten toevoegen aan de probleemlijst?
Traduction base sur traduction de : Michel Roland et Marc Jamoulle (cisp_bureau fr.pdf (uliege.be))

Votre évaluation:

*****

Veuillez sélectionner une seule des propositions suivantes :

- ◯ 1 (Médiocre)
- ◯ 2
- ◯ 3
- ◯ 4
- ◯ 5
- ◯ 6
- ◯ 7

◯ 8

◯ 9 (Excellent)

◯ Non évaluable

Evaluation basée sur:

Veuillez choisir toutes les réponses qui conviennent :

☐ L’extractibilité de le DMI

☐ Recommandation sur la pertinence

Indicateur / recommandation	Source	Année	Niveau de preuve
**INDICATEUR:**<br>**Un certain nombre de médicaments sont prescrits spécifiquement dans le contexte d'une maladie particulière. Pour les patients ayant une ordonnance pour ces médicaments, un élément de soins avec ce diagnostic doit être présent. L'absence d'un tel élément peut indiquer qu'il n'a pas été enregistré dans le DMI.**<br>**Ces maladies sont les suivantes**<br>• **Maladie thyroïdienne**<br>• **épilepsie**<br>• **la maladie de Parkinson**<br>• **la dépression**<br>• **Maladie cardiovasculaire**<br>• **Asthme/COPD**<br>• **Diabète**	**EPD-scan-h**	**2009**	**Pas de gradation**

Votre évaluation:

*

Veuillez sélectionner une seule des propositions suivantes :

- ◯ 1 (Médiocre)
- ◯ 2
- ◯ 3
- ◯ 4
- ◯ 5
- ◯ 6
- ◯ 7
- ◯ 8
- ◯ 9 (Excellent)
- ◯ Non évaluable

Evaluation basée sur:

Veuillez choisir toutes les réponses qui conviennent :

☐ L'extractibilité de le DMI
☐ Recommandation sur la pertinence

Indicateur / Recommandation	Source	Année	Niveau de preuve
**INDICATEUR:** **Pourcentage de patients enregistrés pour lesquels il n'y a pas de changement dans le DMI les 12 derniers mois.**	**De Lusignan et al**	**2002**	**Pas de gradation**

Votre évaluation:

*

Veuillez sélectionner une seule des propositions suivantes :

◯ 1 (Médiocre)
◯ 2
◯ 3
◯ 4
◯ 5
◯ 6
◯ 7
◯ 8
◯ 9 (Excellent)
◯ Non évaluable

Evaluation basée sur:

Veuillez choisir toutes les réponses qui conviennent :

☐ L'extractibilité de le DMI
☐ Recommandation sur la pertinence

Indicateur / Recommandation	Source	Année	Niveau de preuve
**INDICATEUR:** **Pourcentage de la population de patients, âgés de 18 ans et plus, atteints de maladies chroniques qui ont reçu au moins un des types suivants de soutien à l'autogestion de la part de leur prestataire de soins de santé primaires: Fourni un plan de traitement ; Encouragé à utiliser des groupes ou des programmes d'entraide**	**CIHI**	**2016**	**Pas de gradation**

Votre évaluation:

*

Veuillez sélectionner une seule des propositions suivantes :

- ○ 1 (Médiocre)
- ○ 2
- ○ 3
- ○ 4
- ○ 5
- ○ 6
- ○ 7
- ○ 8
- ○ 9 (Excellent)
- ○ Non évaluable

Evaluation basée sur:

Veuillez choisir toutes les réponses qui conviennent :

- ☐ L’extractibilité de le DMI
- ☐ Recommandation sur la pertinence

Indicateur / recommandation	Source	Année	Niveau de preuve
**INDICATEUR:**<br>**Quel est le pourcentage d'élément de santé/soins de la liste d'élément de santé/soins ayant un code ICPC correcte?**<br>**Le code ICPC doit être subdivisé en:**<br>• **Utilisation correcte : plaintes entre 01 et 29 et diagnostics entre 70 et 99. Cette catégorie comprend également les codes A44 (vaccination), R44 (vaccination contre la grippe) et X37 (dépistage de la population par frottis cervical).**<br>• **Utilisation incorrecte possible : A97 (pas de maladie) ou A99 (autre maladie généralisée ou non spécifiée) (en tant que code de vol) ;**<br>• **Non autorisé ou pas d'ICPC : pas d'ICPC ou un code non autorisé (intervalle 30-69), sauf A44 (vaccination préventive ou médicament), R44 (vaccination antigrippale) et X37 (dépistage de la population par frottis du col de l'utérus).**	**EPD-scan-h**	**2009**	**/**

Votre évaluation:

*****

Veuillez sélectionner une seule des propositions suivantes :

◯ 1 (Médiocre)

◯ 2

- 3
- 4
- 5
- 6
- 7
- 8
- 9 (Excellent)
- Non évaluable

Evaluation basée sur:

Veuillez choisir toutes les réponses qui conviennent :

- L’extractibilité de le DMI
- Recommandation sur la pertinence

Indicateur / Recommandation	Source	Année	Niveau de preuve
**RECOMMANDATION:**<br>**Il convient que les restrictions de communication soient enregistrées dans le DMI afin que le prestataire de soins puisse en tenir compte.**<br>**ICPC-2 codes:**<br>• **F94 Cécité**<br>• **H84 Presbyacousie**<br>• **H86 Surdité**<br>• **Dans l'intervalle 28 (fonction limitée/incapacité)**	**NHS**	**2023**	**Pas de gradation**

Votre évaluation:

*

Veuillez sélectionner une seule des propositions suivantes :

- ◯ 1 (Médiocre)
- ◯ 2
- ◯ 3
- ◯ 4
- ◯ 5
- ◯ 6
- ◯ 7
- ◯ 8
- ◯ 9 (Excellent)
- ◯ Non évaluable

Evaluation basée sur:

Veuillez choisir toutes les réponses qui conviennent :

- ☐ L’extractibilité de le DMI
- ☐ Recommandation sur la pertinence

Indicateur / recommandation	Source	Année	Niveau de preuve
**RECOMMANDATION:** **Dans le DMI, il est préférable d'enregistrer les informations supplémentaires concernant le diagnostic dans une légende ou un commentaire qui donne plus d'informations sur les aspects de la maladie.**	**HIQA**	**2018**	**Pas de gradation**

Votre évaluation:

*

Veuillez sélectionner une seule des propositions suivantes :

- ◯ 1 (Médiocre)
- ◯ 2
- ◯ 3
- ◯ 4
- ◯ 5
- ◯ 6
- ◯ 7
- ◯ 8
- ◯ 9 (Excellent)
- ◯ Non évaluable

Evaluation basée sur:

Veuillez choisir toutes les réponses qui conviennent :

- ☐ L’extractibilité de le DMI
- ☐ Recommandation sur la pertinence

Indicateur / recommandation	Source	Année	Niveau de preuve
**RECOMMANDATION:**<br>**Le DMI doit enregistrer la date de début de chaque élément de soins.**	**HIQA**	**2018**	**Pas de gradation**

Votre évaluation:

*

Veuillez sélectionner une seule des propositions suivantes :

- ◯ 1 (Médiocre)
- ◯ 2
- ◯ 3
- ◯ 4
- ◯ 5
- ◯ 6
- ◯ 7
- ◯ 8
- ◯ 9 (Excellent)
- ◯ Non évaluable

Evaluation basée sur:

Veuillez choisir toutes les réponses qui conviennent :

- ☐ L’extractibilité de le DMI
- ☐ Recommandation sur la pertinence

Indicateur / recommandation	Source	Année	Niveau de preuve
**RECOMMANDATION:** **Le DMI doit enregistrer la date de fin ou la date de fin présumée de chaque élément de soins terminé.**	**HIQA**	**2018**	**Pas de gradation**

Votre évaluation:

*

Veuillez sélectionner une seule des propositions suivantes :

- ◯ 1 (Médiocre)
- ◯ 2
- ◯ 3
- ◯ 4
- ◯ 5
- ◯ 6
- ◯ 7
- ◯ 8
- ◯ 9 (Excellent)
- ◯ Non évaluable

Evaluation basée sur:

Veuillez choisir toutes les réponses qui conviennent :

- ☐ L’extractibilité de le DMI
- ☐ Recommandation sur la pertinence

Indicateur / recommandation	Source	Année	Niveau de preuve
**RECOMMANDATION:** **Elle doit être enregistrée dans le DMI si le patient n'a pas de maladie connue. De cette manière, les autres prestataires de soins de santé savent que le dossier est complet. (ICPC-2 : A97)**	**HIQA**	**2018**	**Pas de gradation**

Votre évaluation:

*

Veuillez sélectionner une seule des propositions suivantes :

- ◯ 1 (Médiocre)
- ◯ 2
- ◯ 3
- ◯ 4
- ◯ 5
- ◯ 6
- ◯ 7
- ◯ 8
- ◯ 9 (Excellent)
- ◯ Non évaluable

Evaluation basée sur:

Veuillez choisir toutes les réponses qui conviennent :

- ☐ L’extractibilité de le DMI
- ☐ Recommandation sur la pertinence

Indicateur / recommandation	Source	Année	Niveau de preuve
**RECOMMANDATION:** **Le DMI contient toute condition ou donnée pertinente pour la suite des soins, telle que certaines procédures et conditions susceptibles de se reproduire.** **Clarification : il s'agit des interventions chirurgicales et des traitements majeurs tels que définis dans le NHG Ingrepenviewer**	**ADEPD, Domus Medica, HIQA, NCQA**	**2019, 2004, 2020, 2018**	**Pas de gradation**

Votre évaluation:

*

Veuillez sélectionner une seule des propositions suivantes :

- ◯ 1 (Médiocre)
- ◯ 2
- ◯ 3
- ◯ 4
- ◯ 5
- ◯ 6
- ◯ 7
- ◯ 8
- ◯ 9 (Excellent)
- ◯ Non évaluable

Evaluation basée sur:

Veuillez choisir toutes les réponses qui conviennent :

- ☐ L'extractibilité de le DMI
- ☐ Recommandation sur la pertinence

Indicateur / recommandation	Source	Année	Niveau de preuve
**RECOMMANDATION:** **De préférence, enregistrez les opérations et les traitements majeurs avec l'épisode correspondant. Pour ce faire, utilisez la fonctionnalité de votre DMI. Ces informations figureront également dans l'aperçu des interventions et des traitements.**	**ADEPD**	**2019**	**Pas de gradation**

Votre évaluation:

*

Veuillez sélectionner une seule des propositions suivantes :

- ◯ 1 (Médiocre)
- ◯ 2
- ◯ 3
- ◯ 4
- ◯ 5
- ◯ 6
- ◯ 7
- ◯ 8
- ◯ 9 (Excellent)
- ◯ Non évaluable

Evaluation basée sur:

Veuillez choisir toutes les réponses qui conviennent :

- ☐ L’extractibilité de le DMI
- ☐ Recommandation sur la pertinence

Indicateur / recommandation	Source	Année	Niveau de preuve
**RECOMMANDATION:** **Pour les opérations et procédures concernées, le DMI contient une légende avec des informations sur la procédure.**	**HIQA**	**2018**	**Pas de gradation**

Votre évaluation:

*

Veuillez sélectionner une seule des propositions suivantes :

- ◯ 1 (Médiocre)
- ◯ 2
- ◯ 3
- ◯ 4
- ◯ 5
- ◯ 6
- ◯ 7
- ◯ 8
- ◯ 9 (Excellent)
- ◯ Non évaluable

Evaluation basée sur:

Veuillez choisir toutes les réponses qui conviennent :

- ☐ L'extractibilité de le DMI
- ☐ Recommandation sur la pertinence

Indicator / recommandation	Source	Année	Niveau de preuve
**RECOMMANDATION:** **Pour les opérations et procédures pertinentes, le DMI contient la date à laquelle elles ont été effectuées.**	**HIQA**	**2018**	**Pas de gradation**

Votre évaluation:

*

Veuillez sélectionner une seule des propositions suivantes :

- ◯ 1 (Médiocre)
- ◯ 2
- ◯ 3
- ◯ 4
- ◯ 5
- ◯ 6
- ◯ 7
- ◯ 8
- ◯ 9 (Excellent)
- ◯ Non évaluable

Evaluation basée sur:

Veuillez choisir toutes les réponses qui conviennent :

- ☐ L'extractibilité de le DMI
- ☐ Recommandation sur la pertinence

Indicator / recommandation	Source	Année	Niveau de preuve
**RECOMMANDATION:** **Le DMI contient un enregistrement si le patient n'a pas subi d'opérations ou de procédures antérieures.**	**HIQA**	**2018**	**Pas de gradation**

Votre évaluation:

*

Veuillez sélectionner une seule des propositions suivantes :

- ◯ 1 (Médiocre)
- ◯ 2
- ◯ 3
- ◯ 4
- ◯ 5
- ◯ 6
- ◯ 7
- ◯ 8
- ◯ 9 (Excellent)
- ◯ Non évaluable

Evaluation basée sur:

Veuillez choisir toutes les réponses qui conviennent :

- ☐ L’extractibilité de le DMI
- ☐ Recommandation sur la pertinence

Cinq recommandations principales :

Quelles recommandations concernant "l'exhaustivité et l'adéquation de la liste des problèmes" vous semblent les plus appropriées pour mesurer la qualité d'une bonne utilisation du DMI en médecine générale ?

1.

*

Veuillez sélectionner une seule des propositions suivantes :

◯ En moyenne, combien d'élément de santé/soins actifs un patient a-t-il sur sa liste d'élément de santé/soins ?

◯ Quel est le pourcentage d'élément de santé/soins avec une attention particulière qui sont effectivement étiquetés « valeur d'attention particulière » ?

◯ Un certain nombre de médicaments sont prescrits spécifiquement dans le contexte d'une maladie particulière. Pour les patients ayant une ordonnance pour ces médicaments, un élément de soins avec ce diagnostic doit être présent.

◯ Pourcentage de patients enregistrés pour lesquels il n'y a pas de changement dans le DMI les 12 derniers mois.

◯ Pourcentage de la population de patients, âgés de 18 ans et plus, atteints de maladies chroniques qui ont reçu au moins un des types suivants de soutien à l'autogestion de la part de leur prestataire de soins de santé primaires: Fourni un plan de traitement ; Encouragé à utiliser des groupes ou des programmes d'entraide

◯ Quel est le pourcentage d'élément de santé/soins de la liste d'élément de santé/soins ayant un code ICPC valide ?

◯ Il convient que les restrictions de communication soient enregistrées dans le DMI afin que le prestataire de soins puisse en tenir compte.

◯ Dans le DMI, il est préférable d'enregistrer les informations supplémentaires concernant le diagnostic dans une légende ou un commentaire qui donne plus d'informations sur les aspects de la maladie.

◯ Le DMI doit enregistrer la date de début de chaque élément de soins.

◯ Le DMI doit enregistrer la date de fin ou la date de fin présumée de chaque élément de soins terminé.

◯ Elle doit être enregistrée dans le DMI si le patient n'a pas de maladie connue.

◯ Le DMI contient toute condition ou donnée pertinente pour la suite des soins, telle que certaines procédures et conditions susceptibles de se reproduire.

◯ De préférence, enregistrez les opérations et les traitements majeurs avec l'épisode correspondant.

◯ Pour les opérations et procédures concernées, le DMI contient une légende avec des informations sur la procédure.

◯ Pour les opérations et procédures pertinentes, le DMI contient la date à laquelle elles ont été effectuées.

◯ Le DMI contient un enregistrement si le patient n'a pas subi d'opérations ou de procédures antérieures.

2.

*

Veuillez sélectionner une seule des propositions suivantes :

- ◯ En moyenne, combien d'élément de santé/soins actifs un patient a-t-il sur sa liste d'élément de santé/soins ?
- ◯ Quel est le pourcentage d'élément de santé/soins avec une attention particulière qui sont effectivement étiquetés « valeur d'attention particulière » ?
- ◯ Un certain nombre de médicaments sont prescrits spécifiquement dans le contexte d'une maladie particulière. Pour les patients ayant une ordonnance pour ces médicaments, un élément de soins avec ce diagnostic doit être présent.
- ◯ Pourcentage de patients enregistrés pour lesquels il n'y a pas de changement dans le DMI les 12 derniers mois.
- ◯ Pourcentage de la population de patients, âgés de 18 ans et plus, atteints de maladies chroniques qui ont reçu au moins un des types suivants de soutien à l'autogestion de la part de leur prestataire de soins de santé primaires: Fourni un plan de traitement ; Encouragé à utiliser des groupes ou des programmes d'entraide
- ◯ Quel est le pourcentage d'élément de santé/soins de la liste d'élément de santé/soins ayant un code ICPC valide ?
- ◯ Il convient que les restrictions de communication soient enregistrées dans le DMI afin que le prestataire de soins puisse en tenir compte.
- ◯ Dans le DMI, il est préférable d'enregistrer les informations supplémentaires concernant le diagnostic dans une légende ou un commentaire qui donne plus d'informations sur les aspects de la maladie.
- ◯ Le DMI doit enregistrer la date de début de chaque élément de soins.
- ◯ Le DMI doit enregistrer la date de fin ou la date de fin présumée de chaque élément de soins terminé.
- ◯ Elle doit être enregistrée dans le DMI si le patient n'a pas de maladie connue.
- ◯ Le DMI contient toute condition ou donnée pertinente pour la suite des soins, telle que certaines procédures et conditions susceptibles de se reproduire.
- ◯ De préférence, enregistrez les opérations et les traitements majeurs avec l'épisode correspondant.
- ◯ Pour les opérations et procédures concernées, le DMI contient une légende avec des informations sur la procédure.
- ◯ Pour les opérations et procédures pertinentes, le DMI contient la date à laquelle elles ont été effectuées.
- ◯ Le DMI contient un enregistrement si le patient n'a pas subi d'opérations ou de procédures antérieures.

3.

*

Veuillez sélectionner une seule des propositions suivantes :

- ◯ En moyenne, combien d'élément de santé/soins actifs un patient a-t-il sur sa liste d'élément de santé/soins ?
- ◯ Quel est le pourcentage d'élément de santé/soins avec une attention particulière qui sont effectivement étiquetés « valeur d'attention particulière » ?
- ◯ Un certain nombre de médicaments sont prescrits spécifiquement dans le contexte d'une maladie particulière. Pour les patients ayant une ordonnance pour ces médicaments, un élément de soins avec ce diagnostic doit être présent.
- ◯ Pourcentage de patients enregistrés pour lesquels il n'y a pas de changement dans le DMI les 12 derniers mois.
- ◯ Pourcentage de la population de patients, âgés de 18 ans et plus, atteints de maladies chroniques qui ont reçu au moins un des types suivants de soutien à l'autogestion de la part de leur prestataire de soins de santé primaires: Fourni un plan de traitement ; Encouragé à utiliser des groupes ou des programmes d'entraide
- ◯ Quel est le pourcentage d'élément de santé/soins de la liste d'élément de santé/soins ayant un code ICPC valide ?
- ◯ Il convient que les restrictions de communication soient enregistrées dans le DMI afin que le prestataire de soins puisse en tenir compte.
- ◯ Dans le DMI, il est préférable d'enregistrer les informations supplémentaires concernant le diagnostic dans une légende ou un commentaire qui donne plus d'informations sur les aspects de la maladie.
- ◯ Le DMI doit enregistrer la date de début de chaque élément de soins.
- ◯ Le DMI doit enregistrer la date de fin ou la date de fin présumée de chaque élément de soins terminé.
- ◯ Elle doit être enregistrée dans le DMI si le patient n'a pas de maladie connue.
- ◯ Le DMI contient toute condition ou donnée pertinente pour la suite des soins, telle que certaines procédures et conditions susceptibles de se reproduire.
- ◯ De préférence, enregistrez les opérations et les traitements majeurs avec l'épisode correspondant.
- ◯ Pour les opérations et procédures concernées, le DMI contient une légende avec des informations sur la procédure.
- ◯ Pour les opérations et procédures pertinentes, le DMI contient la date à laquelle elles ont été effectuées.
- ◯ Le DMI contient un enregistrement si le patient n'a pas subi d'opérations ou de procédures antérieures.

4.

*

Veuillez sélectionner une seule des propositions suivantes :

- ◯ En moyenne, combien d'élément de santé/soins actifs un patient a-t-il sur sa liste d'élément de santé/soins ?
- ◯ Quel est le pourcentage d'élément de santé/soins avec une attention particulière qui sont effectivement étiquetés « valeur d'attention particulière » ?
- ◯ Un certain nombre de médicaments sont prescrits spécifiquement dans le contexte d'une maladie particulière. Pour les patients ayant une ordonnance pour ces médicaments, un élément de soins avec ce diagnostic doit être présent.
- ◯ Pourcentage de patients enregistrés pour lesquels il n'y a pas de changement dans le DMI les 12 derniers mois.
- ◯ Pourcentage de la population de patients, âgés de 18 ans et plus, atteints de maladies chroniques qui ont reçu au moins un des types suivants de soutien à l'autogestion de la part de leur prestataire de soins de santé primaires: Fourni un plan de traitement ; Encouragé à utiliser des groupes ou des programmes d'entraide
- ◯ Quel est le pourcentage d'élément de santé/soins de la liste d'élément de santé/soins ayant un code ICPC valide ?
- ◯ Il convient que les restrictions de communication soient enregistrées dans le DMI afin que le prestataire de soins puisse en tenir compte.
- ◯ Dans le DMI, il est préférable d'enregistrer les informations supplémentaires concernant le diagnostic dans une légende ou un commentaire qui donne plus d'informations sur les aspects de la maladie.
- ◯ Le DMI doit enregistrer la date de début de chaque élément de soins.
- ◯ Le DMI doit enregistrer la date de fin ou la date de fin présumée de chaque élément de soins terminé.
- ◯ Elle doit être enregistrée dans le DMI si le patient n'a pas de maladie connue.
- ◯ Le DMI contient toute condition ou donnée pertinente pour la suite des soins, telle que certaines procédures et conditions susceptibles de se reproduire.
- ◯ De préférence, enregistrez les opérations et les traitements majeurs avec l'épisode correspondant.
- ◯ Pour les opérations et procédures concernées, le DMI contient une légende avec des informations sur la procédure.
- ◯ Pour les opérations et procédures pertinentes, le DMI contient la date à laquelle elles ont été effectuées.
- ◯ Le DMI contient un enregistrement si le patient n'a pas subi d'opérations ou de procédures antérieures.

5.

*

Veuillez sélectionner une seule des propositions suivantes :

- ◯ En moyenne, combien d'élément de santé/soins actifs un patient a-t-il sur sa liste d'élément de santé/soins ?
- ◯ Quel est le pourcentage d'élément de santé/soins avec une attention particulière qui sont effectivement étiquetés « valeur d'attention particulière » ?
- ◯ Un certain nombre de médicaments sont prescrits spécifiquement dans le contexte d'une maladie particulière. Pour les patients ayant une ordonnance pour ces médicaments, un élément de soins avec ce diagnostic doit être présent.
- ◯ Pourcentage de patients enregistrés pour lesquels il n'y a pas de changement dans le DMI les 12 derniers mois.
- ◯ Pourcentage de la population de patients, âgés de 18 ans et plus, atteints de maladies chroniques qui ont reçu au moins un des types suivants de soutien à l'autogestion de la part de leur prestataire de soins de santé primaires: Fourni un plan de traitement ; Encouragé à utiliser des groupes ou des programmes d'entraide
- ◯ Quel est le pourcentage d'élément de santé/soins de la liste d'élément de santé/soins ayant un code ICPC valide ?
- ◯ Il convient que les restrictions de communication soient enregistrées dans le DMI afin que le prestataire de soins puisse en tenir compte.
- ◯ Dans le DMI, il est préférable d'enregistrer les informations supplémentaires concernant le diagnostic dans une légende ou un commentaire qui donne plus d'informations sur les aspects de la maladie.
- ◯ Le DMI doit enregistrer la date de début de chaque élément de soins.
- ◯ Le DMI doit enregistrer la date de fin ou la date de fin présumée de chaque élément de soins terminé.
- ◯ Elle doit être enregistrée dans le DMI si le patient n'a pas de maladie connue.
- ◯ Le DMI contient toute condition ou donnée pertinente pour la suite des soins, telle que certaines procédures et conditions susceptibles de se reproduire.
- ◯ De préférence, enregistrez les opérations et les traitements majeurs avec l'épisode correspondant.
- ◯ Pour les opérations et procédures concernées, le DMI contient une légende avec des informations sur la procédure.
- ◯ Pour les opérations et procédures pertinentes, le DMI contient la date à laquelle elles ont été effectuées.
- ◯ Le DMI contient un enregistrement si le patient n'a pas subi d'opérations ou de procédures antérieures.

Si vous avez des suggestions de recommandations qui, selon vous, ne figurent pas dans la liste ci-dessus, vous pouvez les noter dans l'encadré ci-dessous.

Veuillez toujours indiquer clairement une recommandation et sa motivation.

Veuillez écrire votre réponse ici :

2. Enregistrement structuré dans le DMI

Dans quelle mesure les recommandations suivantes sont-elles pertinentes pour mesurer la qualité d'une bonne utilisation du DMI en médecine générale en ce qui concerne **l'enregistrement structuré des consultations dans le DMI**?

Indicateur / recommandation	Source	Année	Niveau de preuve
**INDICATEUR:**<br>**Quel est le pourcentage de sous-contacts dans le journal connecté avecdes éléments de soins/santé avec un code ICPC valide?**<br>**Le code ICPC doit être subdivisé en:**<br>• **Utilisation correcte: plaintes entre 01 et 29 et diagnostics entre 70 et 99. Cette catégorie comprend également les codes A44 (vaccination), R44 (vaccination contre la grippe) et X37 (dépistage de la population par frottis cervical).**<br>• **Utilisation incorrecte possible : A97 (pas de maladie) ou A99 (autre maladie généralisée ou non spécifiée) (en tant que code de vol) ;**<br>• **Non autorisé ou pas d'ICPC : pas d'ICPC ou un code non autorisé (intervalle 30-69), sauf A44 (vaccination préventive ou médicament), R44 (vaccination antigrippale) et X37 (dépistage de la population par frottis du col de l'utérus).**	**EPD-scan-h**	**2009**	**Pas de gradation**

Votre évaluation:

*

Veuillez sélectionner une seule des propositions suivantes :

◯ 1 (Médiocre)

◯ 2

- ◯ 3
- ◯ 4
- ◯ 5
- ◯ 6
- ◯ 7
- ◯ 8
- ◯ 9 (Excellent)
- ◯ Non évaluable

Evaluation basée sur:

Veuillez choisir toutes les réponses qui conviennent :

- ☐ L’extractibilité de le DMI
- ☐ Recommandation sur la pertinence

Indicateur / recommandation	Source	Année	Niveau de preuve
**INDICATEUR:**<br>**Quel est le pourcentage de contacts partiels enregistrés lors de consultations, de contacts téléphoniques et de visites à domicile qui comportent une ligne Subjectif, Objectif, Evaluation ou Planning?**	**EPD-scan-h**	**2009**	**Pas de gradation**
**RECOMMANDATION LIEE:**<br>**Rapport de contact partiel : le DMI présente les règles SOEP du du sous-contact auquel le médecin généraliste se réfère.**<br>**Le médecin généraliste sélectionne d'autres sous-contacts de l'épisode si nécessaire.**	**HASP**	**2018**	**Pas de gradation**

Votre évaluation:

*

Veuillez sélectionner une seule des propositions suivantes :

- ◯ 1 (Médiocre)
- ◯ 2
- ◯ 3
- ◯ 4
- ◯ 5
- ◯ 6
- ◯ 7
- ◯ 8
- ◯ 9 (Excellent)
- ◯ Non évaluable

Evaluation basée sur:

Veuillez choisir toutes les réponses qui conviennent :

- [ ] L’extractibilité de le DMI
- [ ] Recommandation sur la pertinence

Indicateur / recommandation	Source	Année	Niveau de preuve
**INDICATEUR:** **Nombre de consultations pour les sources online (par example. CDLH)**	**Hamade et al.**	**2008**	**Pas de gradation**

Votre évaluation:

*

Veuillez sélectionner une seule des propositions suivantes :

- ◯ 1 (Médiocre)
- ◯ 2
- ◯ 3
- ◯ 4
- ◯ 5
- ◯ 6
- ◯ 7
- ◯ 8
- ◯ 9 (Excellent)
- ◯ Non évaluable

Evaluation basée sur:

Veuillez choisir toutes les réponses qui conviennent :

- [ ] L’extractibilité de le DMI
- [ ] Recommandation sur la pertinence

Indicateur / recommandation	Source	Année	Niveau de preuve
**INDICATEUR:**<br>**Nombre de changements d'horaires par semaine pour 1000 patients avec dossier médical global**	**Hamade et al.**	**2008**	**Pas de gradation**
**RECOMMANDATION LIEE:**<br>**Encounter forms or notes have a notation, regarding follow-up care, calls or visits, when indicated. The specific time of return is noted in weeks, months or as needed.**	**NCQA**	**2018**	**Pas de gradation**

Votre évaluation:

*

Veuillez sélectionner une seule des propositions suivantes :

- ◯ 1 (Médiocre)
- ◯ 2
- ◯ 3
- ◯ 4
- ◯ 5
- ◯ 6
- ◯ 7
- ◯ 8
- ◯ 9 (Excellent)
- ◯ Non évaluable

Evaluation basée sur:

Veuillez choisir toutes les réponses qui conviennent :

- ☐ L'extractibilité de le DMI
- ☐ Recommandation sur la pertinence

Indicateur / recommandation	Source	Année	Niveau de preuve
**RECOMMANDATION:**<br>**Consultation, laboratory and imaging reports filed in the chart are initialed by the practitioner who ordered them, to signify review. (Review and signature by professionals other than the ordering practitioner do not meet this requirement.) If the reports are presented electronically or by some other method, there is also representation of review by the ordering practitioner. Consultation and abnormal laboratory and imaging study results have an explicit notation in the record of followup plans.**	**NCQA**	**2018**	**Pas de gradation**

Votre évaluation:

*

Veuillez sélectionner une seule des propositions suivantes :

- ◯ 1 (Médiocre)
- ◯ 2
- ◯ 3
- ◯ 4
- ◯ 5
- ◯ 6
- ◯ 7
- ◯ 8
- ◯ 9 (Excellent)
- ◯ Non évaluable

Evaluation basée sur:

Veuillez choisir toutes les réponses qui conviennent :

☐ L'extractibilité de le DMI
☐ Recommandation sur la pertinence

Cinq recommandations principales :

Quelles recommandations concernant "exhaustivité et actualisation de la liste des médicaments" vous semblent les plus appropriées pour mesurer la qualité d'une bonne utilisation du DMI en médecine générale ?

1.

*

Veuillez sélectionner une seule des propositions suivantes :

◯ Quel est le pourcentage de sous-contacts dans le journal connecté avecdes éléments de soins/santé avec un code ICPC valide?

◯ Quel est le pourcentage de contacts partiels enregistrés lors de consultations, de contacts téléphoniques et de visites à domicile qui comportent une ligne Subjectif, Objectif, Evaluation ou Planning?

◯ Nombre de consultations pour les sources online (par example. CDLH)

◯ Nombre de changements d'horaires par semaine pour 1000 patients avec dossier médical global

◯ Consultation, laboratory and imaging reports filed in the chart are initialed by the practitioner who ordered them, to signify review

2.

*

Veuillez sélectionner une seule des propositions suivantes :

◯ Quel est le pourcentage de sous-contacts dans le journal connecté avecdes éléments de soins/santé avec un code ICPC valide?

◯ Quel est le pourcentage de contacts partiels enregistrés lors de consultations, de contacts téléphoniques et de visites à domicile qui comportent une ligne Subjectif, Objectif, Evaluation ou Planning?

◯ Nombre de consultations pour les sources online (par example. CDLH)

◯ Nombre de changements d'horaires par semaine pour 1000 patients avec dossier médical global

◯ Consultation, laboratory and imaging reports filed in the chart are initialed by the practitioner who ordered them, to signify review

3.

*

Veuillez sélectionner une seule des propositions suivantes :

◯ Quel est le pourcentage de sous-contacts dans le journal connecté avecdes éléments de soins/santé avec un code ICPC valide?

◯ Quel est le pourcentage de contacts partiels enregistrés lors de consultations, de contacts téléphoniques et de visites à domicile qui comportent une ligne Subjectif, Objectif, Evaluation ou Planning?

◯ Nombre de consultations pour les sources online (par example. CDLH)

◯ Nombre de changements d'horaires par semaine pour 1000 patients avec dossier médical global

◯ Consultation, laboratory and imaging reports filed in the chart are initialed by the practitioner who ordered them, to signify review

Si vous avez des suggestions de recommandations qui, selon vous, ne figurent pas dans la liste ci-dessus, vous pouvez les noter dans l'encadré ci-dessous.

Veuillez toujours indiquer clairement une recommandation et sa motivation.

Veuillez écrire votre réponse ici :

3. Exhaustivité et actualisation de la liste des médicaments

Dans quelle mesure les recommandations suivantes sont-elles pertinentes pour mesurer la qualité d'une bonne utilisation du DMI en médecine générale en ce qui concerne **l'exhaustivité et actualisation de la liste des médicaments** des patients?

Indicateur / recommandation	Source	Année	Niveau de preuve
**INDICATEUR:**<br>**Quel est le pourcentage des médicaments sur la liste des « médicaments actuels » qui sont incorrectement étiquetées comme étant des médicaments actuels?**<br>**Le résumé professionnel devrait mentionner les médicaments actuels (c'est-à-dire les médicaments que le patient prend actuellement, que ce soit de manière temporaire ou chronique (>6 mois)) et les médicaments arrêtés au cours des derniers mois (4-6 mois selon la source). En règle générale, un médecin généraliste prescrit des médicaments pour trois mois, avec des exceptions à six mois pour la pilule, par exemple. Les médicaments non prescrits depuis plus de six mois ne sont vraisemblablement plus d'actualité.**	**EPD-scan-h**	**2009**	**Pas de gradation**
**RECOMMANDATION LIEE:**<br>**Le DMI présente les prescriptions actuelles. Le DMI permet d'ajouter des médicaments antérieurs à l'épisode dont il est question, y compris la raison de l'arrêt, et/ou l'automédication.**	**NHG ADEP, Domus Medica, SSMG, NHS, HIQA**	**2019, 2004, ?, 2023, 2020**	**Pas de gradation**

Votre évaluation:

*

Veuillez sélectionner une seule des propositions suivantes :

◯ 1 (Médiocre)

- ◯ 2
- ◯ 3
- ◯ 4
- ◯ 5
- ◯ 6
- ◯ 7
- ◯ 8
- ◯ 9 (Excellent)
- ◯ Non évaluable

Evaluation basée sur:

Veuillez choisir toutes les réponses qui conviennent :

- ☐ L’extractibilité de le DMI
- ☐ Recommandation sur la pertinence

Indicateur / recommandation	Source	Année	Niveau de preuve
**INDICATEUR:**<br>**Quel est le pourcentage de prescriptions connecté avec un épisode de soins/santé?**<br>**Pour cet indicateur, nous avons sélectionné les médicaments actuels et les médicaments arrêtés au cours des quatre derniers mois.**<br>**Le code ICPC doit être subdivisé en :**<br>• **Utilisation correcte : plaintes entre 01 et 29 et diagnostics entre 70 et 99. Cette catégorie comprend également les codes A44 (vaccination), R44 (vaccination contre la grippe) et X37 (dépistage de la population par frottis cervical).**<br>• **Utilisation incorrecte possible : A97 (pas de maladie) ou A99 (autre maladie généralisée ou non spécifiée) (en tant que code de vol) ;**<br>• **Non autorisé ou pas d'ICPC : pas d'ICPC ou un code non autorisé (intervalle 30-69), sauf A44 (vaccination préventive ou médicament), R44 (vaccination antigrippale) et X37 (dépistage de la population par frottis du col de l'utérus).**	**EPD-scan-h**	**2009**	**Pas de gradation**

Votre évaluation:

*****

Veuillez sélectionner une seule des propositions suivantes :

- ◯ 1 (Médiocre)
- ◯ 2
- ◯ 3
- ◯ 4
- ◯ 5
- ◯ 6
- ◯ 7
- ◯ 8
- ◯ 9 (Excellent)
- ◯ Non évaluable

Evaluation basée sur:

Veuillez choisir toutes les réponses qui conviennent :

- ☐ L’extractibilité de le DMI
- ☐ Recommandation sur la pertinence

Recommandation	Source	Année	Niveau de preuve
**INDICATEUR:**<br>**Détails complets sur la dose et le régime concernant l'effet de la dose ou les réactions indésirables aux médicaments.**	**De Lusignan et al**	**2002**	**Pas de gradation**
**RECOMMENDATION LIEE:**<br>• **Pour chaque médicament prescrit, la posologie doit être enregistrée dans le DMI.**<br>• **Pour chaque médicament prescrit, la moment d'admission doit être enregistrée dans le DMI.**<br>• **Pour chaque médicament prescrit, la durée d'administration doit être enregistrée dans le DMI.**<br>• **Le mode d'administration doit être consigné dans le DMI pour chaque médicament prescrit.**<br>• **Pour chaque médicament prescrit, la date de début doit être enregistrée dans le DMI.**	**HIQA**	**2018**	**Pas de gradation**

Votre évaluation:

*****

Veuillez sélectionner une seule des propositions suivantes :

- ◯ 1 (Médiocre)
- ◯ 2
- ◯ 3
- ◯ 4
- ◯ 5
- ◯ 6
- ◯ 7

- 8
- 9 (Excellent)
- Non évaluable

Evaluation basée sur:

Veuillez choisir toutes les réponses qui conviennent :

- L’extractibilité de le DMI
- Recommandation sur la pertinence

Indicateur / recommandation	Source	Année	Niveau de preuve
**INDICATEUR:** **Nombre de prescriptions de médicaments par semaine pour 1 000 patients avec dossier médical global?** **Il s'agit d'une mesure approximative du nombre d'ordonnances qui ne sont pas rédigées sous forme numérique. Il s'agit également d'un outil de détection des données manquantes au fil du temps.**	**De Lusignan.**	**2002**	**/**

Votre évaluation:

*

Veuillez sélectionner une seule des propositions suivantes :

- ◯ 1 (Médiocre)
- ◯ 2
- ◯ 3
- ◯ 4
- ◯ 5
- ◯ 6
- ◯ 7
- ◯ 8
- ◯ 9 (Excellent)
- ◯ Non évaluable

Evaluation basée sur:

Veuillez choisir toutes les réponses qui conviennent :

- ☐ L'extractibilité de le DMI
- ☐ Recommandation sur la pertinence

Indicateur / recommandation	Source	Année	Niveau de preuve
**RECOMMANDATION:** **Si le patient ne prend pas de médicaments, cela doit être correctement noté dans le DMI.**	**HIQA**	**2018**	**Pas de gradation**

Votre évaluation:

*

Veuillez sélectionner une seule des propositions suivantes :

- ◯ 1 (Médiocre)
- ◯ 2
- ◯ 3
- ◯ 4
- ◯ 5
- ◯ 6
- ◯ 7
- ◯ 8
- ◯ 9 (Excellent)
- ◯ Non évaluable

Evaluation basée sur:

Veuillez choisir toutes les réponses qui conviennent :

- ☐ L'extractibilité de le DMI
- ☐ Recommandation sur la pertinence

Trois recommandations principales :

Quelles recommandations concernant "exhaustivité et actualisation de la liste des médicaments" vous semblent les plus appropriées pour mesurer la qualité d'une bonne utilisation du DMI en médecine générale ?

1.

*

Veuillez sélectionner une seule des propositions suivantes :

◯ Quel est le pourcentage des médicaments sur la liste des « médicaments actuels » qui sont incorrectement étiquetées comme étant des médicaments actuels?

◯ Quel est le pourcentage de prescriptions connecté avec un épisode de soins/santé?

◯ Détails complets sur la dose et le régime concernant l'effet de la dose ou les réactions indésirables aux médicaments.

◯ Si le patient ne prend pas de médicaments, cela doit être correctement noté dans le DMI.

◯ Nombre de prescriptions de médicaments par semaine pour 1 000 patients avec dossier médical global?

2.

*****

Veuillez sélectionner une seule des propositions suivantes :

◯ Quel est le pourcentage des médicaments sur la liste des « médicaments actuels » qui sont incorrectement étiquetées comme étant des médicaments actuels?

◯ Quel est le pourcentage de prescriptions connecté avec un épisode de soins/santé?

◯ Détails complets sur la dose et le régime concernant l'effet de la dose ou les réactions indésirables aux médicaments.

◯ Si le patient ne prend pas de médicaments, cela doit être correctement noté dans le DMI.

◯ Nombre de prescriptions de médicaments par semaine pour 1 000 patients avec dossier médical global?

3.

*****

Veuillez sélectionner une seule des propositions suivantes :

◯ Quel est le pourcentage des médicaments sur la liste des « médicaments actuels » qui sont incorrectement étiquetées comme étant des médicaments actuels?

◯ Quel est le pourcentage de prescriptions connecté avec un épisode de soins/santé?

◯ Détails complets sur la dose et le régime concernant l'effet de la dose ou les réactions indésirables aux médicaments.

◯ Si le patient ne prend pas de médicaments, cela doit être correctement noté dans le DMI.

◯ Nombre de prescriptions de médicaments par semaine pour 1 000 patients avec dossier médical global?

Si vous avez des suggestions de recommandations qui, selon vous, ne figurent pas dans la liste ci-dessus, vous pouvez les noter dans l'encadré ci-dessous.

Veuillez toujours indiquer clairement une recommandation et sa motivation.

Veuillez écrire votre réponse ici :

4. Facteurs de risque / surveillance des médicaments

Dans quelle mesure les recommandations suivantes sont-elles pertinentes pour mesurer la qualité d'une bonne utilisation du DMI en médecine générale en ce qui concerne **les facteurs de risque et le surveillance des médicaments du patient** dans le DMI?

Indicateur / recommandation	Source	Année	Niveau de preuve
**INDICATEUR:** **Nombre de nouveaux programmes de dépistage du cancer colorectal ou du cancer du sein par semaine pour 1000 patients avec dossier médical global**	**Hamade et al.**	**2008**	**Pas de gradation**

Votre évaluation:

*

Veuillez sélectionner une seule des propositions suivantes :

- ◯ 1 (Médiocre)
- ◯ 2
- ◯ 3
- ◯ 4
- ◯ 5
- ◯ 6
- ◯ 7
- ◯ 8
- ◯ 9 (Excellent)
- ◯ Non évaluable

Evaluation basée sur:

Veuillez choisir toutes les réponses qui conviennent :

- ☐ L'extractibilité de le DMI
- ☐ Recommandation sur la pertinence

Indicateur / recommandation	Source	Année	Niveau de preuve
**INDICATEUR:** **Combien de patients ont des médicaments contre-indiqués enregistrés ?** **Les contre-indications sont des conditions qui doivent être prises en compte lors de la prescription de médicaments. Il s'agit par exemple du diabète sucré ou de l'insuffisance rénale. Il peut également s'agir de contre-indications temporaires telles que la grossesse.**	**EPD-scan-h**	**2009**	**Pas de gradation**
**RECOMMANDATION LIEE:** **Hypersensibilité aux médicaments, contre-indications à la prescription. Le DMI présente respectivement une hypersensibilité et une contre-indication aux médicaments.**	**ADEPD**	**2019**	**Pas de gradation**

Votre évaluation:

*

Veuillez sélectionner une seule des propositions suivantes :

- ◯ 1 (Médiocre)
- ◯ 2
- ◯ 3
- ◯ 4
- ◯ 5
- ◯ 6
- ◯ 7
- ◯ 8

◯ 9 (Excellent)

◯ Non évaluable

Evaluation basée sur:

Veuillez choisir toutes les réponses qui conviennent :

☐ L’extractibilité de le DMI

☐ Recommandation sur la pertinence

Indicateur / recommandation	Source	Année	Niveau de preuve
**INDICATEUR:**<br>**Pour combien de patients une allergie ou une intolérance aux médicaments a-t-elle été enregistrée ?**	**EPD-scan-h**	**2008**	**Pas de gradation**
**RECOMMANDATION LIEE:**<br>**The patient summary SHALL identify the substance that the patient has a susceptibility to an allergy upon exposure to the substance. It includes allergies, intolerances and adverse reactions to all substances,not only those arising from medications or medicines. It also describes other clinical information that is imperative to know so that the life or health of the patient does not come under threat. For example, intolerance to aspirin due to gastrointestinal bleeding.**<br>**The patient summary SHOULD describe the type of reaction event as determined by the healthcare practitioner.**<br>**The patient summary SHOULD include the severity of the symptom as determined by the healthcare practitioner.**<br>**The patient summary SHOULD contain a record of the date and or time of the onset of the reaction.**	**HIQA**	**2020**	**Pas de gradation**

Votre évaluation:

*

Veuillez sélectionner une seule des propositions suivantes :

- ◯ 1 (Médiocre)
- ◯ 2
- ◯ 3
- ◯ 4
- ◯ 5
- ◯ 6
- ◯ 7
- ◯ 8
- ◯ 9 (Excellent)
- ◯ Non évaluable

Evaluation basée sur:

Veuillez choisir toutes les réponses qui conviennent :

- ☐ L’extractibilité de le DMI
- ☐ Recommandation sur la pertinence

Indicateur / recommandation	Source	Année	Niveau de preuve
**RECOMMANDATION:** **Certaines informations médicales sont importantes pour l'ensemble de la chaîne de soins. Le NHG recommande l'enregistrement des informations suivantes : prophylaxie de la crise d'Addison, endocardite, maladies hémorragiques, endoprothèse, patient immunodéprimé, asplénie (fonctionnelle), thrombose ou micro-organismes particulièrement résistants.**	**ADEPD**	**2019**	**Pas de gradation**

Votre évaluation:

*

Veuillez sélectionner une seule des propositions suivantes :

- ◯ 1 (Médiocre)
- ◯ 2
- ◯ 3
- ◯ 4
- ◯ 5
- ◯ 6
- ◯ 7
- ◯ 8
- ◯ 9 (Excellent)
- ◯ Non évaluable

Evaluation basée sur:

Veuillez choisir toutes les réponses qui conviennent :

- ☐ L’extractibilité de le DMI
- ☐ Recommandation sur la pertinence

Indicateur / recommandation	Source	Année	Niveau de preuve
**RECOMMANDATION:** **L'examen physique peut être enregistré en texte libre ou en tant que diagnostic dans le DMI. S'il est enregistré en tant que diagnostic, il peut être réutilisé pour des aperçus dans le temps et pour l'aide à la décision. Dans la mesure du possible, l'examen physique doit donc être enregistré en tant que diagnostic dans le DMI.** **Examples des diagnostic: le poids, la taille, la tension artérielle, le rythme cardiaque, la consommation d'alcool et de nicotine, la consommation de substances, l'intensité de l'activité physique....** **Une liste complète peut être consultée à l'adresse suivante: NHG - Bepalingenviewer**	**ADEPD**	**2019**	**Pas de gradation**

Votre évaluation:

*

Veuillez sélectionner une seule des propositions suivantes :

- ◯ 1 (Médiocre)
- ◯ 2
- ◯ 3
- ◯ 4
- ◯ 5
- ◯ 6
- ◯ 7
- ◯ 8
- ◯ 9 (Excellent)
- ◯ Non évaluable

Evaluation basée sur:

Veuillez choisir toutes les réponses qui conviennent :

☐ L’extractibilité de le DMI
☐ Recommandation sur la pertinence

Indicateur / recommandation	Source	Année	Niveau de preuve
**RECOMMANDATION:** **Pour tous les patients de 12 ans et plus, est notée dans le DMI si le patient utilise des cigarettes, d'alcool et de substances.**	**NCQA, HASP, NHS**	**2018, 2017, 2023**	**Pas de gradation**

Votre évaluation:

*

Veuillez sélectionner une seule des propositions suivantes :

◯ 1 (Médiocre)
◯ 2
◯ 3
◯ 4
◯ 5
◯ 6
◯ 7
◯ 8
◯ 9 (Excellent)
◯ Non évaluable

Evaluation basée sur:

Veuillez choisir toutes les réponses qui conviennent :

☐ L’extractibilité de le DMI
☐ Recommandation sur la pertinence

Indicateur / recommandation	Source	Année	Niveau de preuve
**RECOMMANDATION:** **Si la section "Anamnèse familiale" est présente dans le DMI: enregistrez ici les informations sur les troubles qui surviennent chez les membres de la famille, pour chaque trouble chez quels membres de la famille il survient, à quel âge il a commencé chez eux et éventuellement à quel âge ils en sont morts ; - la source de ces informations est souvent le patient lui-même - laissez les champs vides si les informations ne sont pas claires ou si elles sont inconnues.**	**ADEPH**	**2019**	**Pas de gradation**

Votre évaluation:

*

Veuillez sélectionner une seule des propositions suivantes :

- ◯ 1 (Médiocre)
- ◯ 2
- ◯ 3
- ◯ 4
- ◯ 5
- ◯ 6
- ◯ 7
- ◯ 8
- ◯ 9 (Excellent)
- ◯ Non évaluable

Evaluation basée sur:

Veuillez choisir toutes les réponses qui conviennent :

- [ ] L'extractibilité de le DMI
- [ ] Recommandation sur la pertinence

Indicateur / recommandation	Source	Année	Niveau de preuve
**RECOMMANDATION:** **Si le patient n'a pas d'allergies connues ou d'antécédents de réactions indésirables, cela est noté de manière appropriée dans le dossier.**	**NCQA HIQA**	**2018 2020**	**Pas de gradation**

Votre évaluation:

*

Veuillez sélectionner une seule des propositions suivantes :

- ◯ 1 (Médiocre)
- ◯ 2
- ◯ 3
- ◯ 4
- ◯ 5
- ◯ 6
- ◯ 7
- ◯ 8
- ◯ 9 (Excellent)
- ◯ Non évaluable

Evaluation basée sur:

Veuillez choisir toutes les réponses qui conviennent :

- [ ] L'extractibilité de le DMI
- [ ] Recommandation sur la pertinence

Indicateur / recommandation	Source	Année	Niveau de preuve
**RECOMMANDATION:** **Examen psychogériatrique : si une personne fragile est sélectionnée, cela est noté dans le DMI dans les sections convenues concernant la fragilité.**	**HASP**	**2017**	**Pas de gradation**

Votre évaluation:

*

Veuillez sélectionner une seule des propositions suivantes :

- ○ 1 (Médiocre)
- ○ 2
- ○ 3
- ○ 4
- ○ 5
- ○ 6
- ○ 7
- ○ 8
- ○ 9 (Excellent)
- ○ Non évaluable

Evaluation basée sur:

Veuillez choisir toutes les réponses qui conviennent :

- ☐ L’extractibilité de le DMI
- ☐ Recommandation sur la pertinence

Indicateur / recommandation	Source	Année	Niveau de preuve
**RECOMMANDATION:**<br>**Les données sociales sont enregistrées dans la section du fichier consacrée aux données sociales. Les données sociales comprennent l'éducation, la profession, l'emploi et le statut socio-économique. La langue et l'alphabétisation, les compétences en matière de santé, la situation du logement, la situation familiale, la présence de soins informels, l'historique des migrations, les croyances et les événements émotionnels intenses survenus dans le passé sont également inclus dans les données sociales.**	**ADEPD**	**2018**	**Pas de gradation**

Votre évaluation:

*

Veuillez sélectionner une seule des propositions suivantes :

- 1 (Médiocre)
- 2
- 3
- 4
- 5
- 6
- 7
- 8
- 9 (Excellent)
- Non évaluable

Evaluation basée sur:

Veuillez choisir toutes les réponses qui conviennent :

- ☐ L’extractibilité de le DMI
- ☐ Recommandation sur la pertinence

Cinq recommandations principales :

Quelles recommandations concernant "facteurs de risque / surveillance des médicaments" vous semblent les plus appropriées pour mesurer la qualité d'une bonne utilisation du DMI en médecine générale ?

1.

*

Veuillez sélectionner une seule des propositions suivantes :

- ○ Nombre de nouveaux programmes de dépistage du cancer colorectal ou du cancer du sein par semaine pour 1000 patients avec dossier médical global.
- ○ Combien de patients ont des médicaments contre-indiqués enregistrés ?
- ○ Pour combien de patients une allergie ou une intolérance aux médicaments a-t-elle été enregistrée ?
- ○ Le NHG recommande l'enregistrement des informations suivantes : prophylaxie de la crise d'Addison, endocardite, maladies hémorragiques, endoprothèse, patient immunodéprimé, asplénie (fonctionnelle), thrombose ou micro-organismes particulièrement résistants.
- ○ Dans la mesure du possible, l'examen physique doit donc être enregistré en tant que diagnostic dans le DMI.
- ○ Pour tous les patients de 12 ans et plus, est notée dans le DMI si le patient utilise des cigarettes, d'alcool et de substances.
- ○ Si la section "Anamnèse familiale" est présente dans le DMI: enregistrez ici les informations sur les troubles qui surviennent chez les membres de la famille
- ○ Si le patient n'a pas d'allergies connues ou d'antécédents de réactions indésirables, cela est noté de manière appropriée dans le dossier.
- ○ Examen psychogériatrique : si une personne fragile est sélectionnée, cela est noté dans le DMI dans les sections convenues concernant la fragilité.
- ○ Les données sociales sont enregistrées dans la section du fichier consacrée aux données sociales.

2.

*

Veuillez sélectionner une seule des propositions suivantes :

- ◯ Nombre de nouveaux programmes de dépistage du cancer colorectal ou du cancer du sein par semaine pour 1000 patients avec dossier médical global.
- ◯ Combien de patients ont des médicaments contre-indiqués enregistrés ?
- ◯ Pour combien de patients une allergie ou une intolérance aux médicaments a-t-elle été enregistrée ?
- ◯ Le NHG recommande l'enregistrement des informations suivantes : prophylaxie de la crise d'Addison, endocardite, maladies hémorragiques, endoprothèse, patient immunodéprimé, asplénie (fonctionnelle), thrombose ou micro-organismes particulièrement résistants.
- ◯ Dans la mesure du possible, l'examen physique doit donc être enregistré en tant que diagnostic dans le DMI.
- ◯ Pour tous les patients de 12 ans et plus, est notée dans le DMI si le patient utilise des cigarettes, d'alcool et de substances.
- ◯ Si la section "Anamnèse familiale" est présente dans le DMI: enregistrez ici les informations sur les troubles qui surviennent chez les membres de la famille
- ◯ Si le patient n'a pas d'allergies connues ou d'antécédents de réactions indésirables, cela est noté de manière appropriée dans le dossier.
- ◯ Examen psychogériatrique : si une personne fragile est sélectionnée, cela est noté dans le DMI dans les sections convenues concernant la fragilité.
- ◯ Les données sociales sont enregistrées dans la section du fichier consacrée aux données sociales.

3.

*

Veuillez sélectionner une seule des propositions suivantes :

- ◯ Nombre de nouveaux programmes de dépistage du cancer colorectal ou du cancer du sein par semaine pour 1000 patients avec dossier médical global.
- ◯ Combien de patients ont des médicaments contre-indiqués enregistrés ?
- ◯ Pour combien de patients une allergie ou une intolérance aux médicaments a-t-elle été enregistrée ?
- ◯ Le NHG recommande l'enregistrement des informations suivantes : prophylaxie de la crise d'Addison, endocardite, maladies hémorragiques, endoprothèse, patient immunodéprimé, asplénie (fonctionnelle), thrombose ou micro-organismes particulièrement résistants.
- ◯ Dans la mesure du possible, l'examen physique doit donc être enregistré en tant que diagnostic dans le DMI.
- ◯ Pour tous les patients de 12 ans et plus, est notée dans le DMI si le patient utilise des cigarettes, d'alcool et de substances.
- ◯ Si la section "Anamnèse familiale" est présente dans le DMI: enregistrez ici les informations sur les troubles qui surviennent chez les membres de la famille
- ◯ Si le patient n'a pas d'allergies connues ou d'antécédents de réactions indésirables, cela est noté de manière appropriée dans le dossier.
- ◯ Examen psychogériatrique : si une personne fragile est sélectionnée, cela est noté dans le DMI dans les sections convenues concernant la fragilité.
- ◯ Les données sociales sont enregistrées dans la section du fichier consacrée aux données sociales.

4.

*

Veuillez sélectionner une seule des propositions suivantes :

◯ Nombre de nouveaux programmes de dépistage du cancer colorectal ou du cancer du sein par semaine pour 1000 patients avec dossier médical global.

◯ Combien de patients ont des médicaments contre-indiqués enregistrés ?

◯ Pour combien de patients une allergie ou une intolérance aux médicaments a-t-elle été enregistrée ?

◯ Le NHG recommande l'enregistrement des informations suivantes : prophylaxie de la crise d'Addison, endocardite, maladies hémorragiques, endoprothèse, patient immunodéprimé, asplénie (fonctionnelle), thrombose ou micro-organismes particulièrement résistants.

◯ Dans la mesure du possible, l'examen physique doit donc être enregistré en tant que diagnostic dans le DMI.

◯ Pour tous les patients de 12 ans et plus, est notée dans le DMI si le patient utilise des cigarettes, d'alcool et de substances.

◯ Si la section "Anamnèse familiale" est présente dans le DMI: enregistrez ici les informations sur les troubles qui surviennent chez les membres de la famille

◯ Si le patient n'a pas d'allergies connues ou d'antécédents de réactions indésirables, cela est noté de manière appropriée dans le dossier.

◯ Examen psychogériatrique : si une personne fragile est sélectionnée, cela est noté dans le DMI dans les sections convenues concernant la fragilité.

◯ Les données sociales sont enregistrées dans la section du fichier consacrée aux données sociales.

5.

*

Veuillez sélectionner une seule des propositions suivantes :

◯ Nombre de nouveaux programmes de dépistage du cancer colorectal ou du cancer du sein par semaine pour 1000 patients avec dossier médical global.

◯ Combien de patients ont des médicaments contre-indiqués enregistrés ?

◯ Pour combien de patients une allergie ou une intolérance aux médicaments a-t-elle été enregistrée ?

◯ Le NHG recommande l'enregistrement des informations suivantes : prophylaxie de la crise d'Addison, endocardite, maladies hémorragiques, endoprothèse, patient immunodéprimé, asplénie (fonctionnelle), thrombose ou micro-organismes particulièrement résistants.

◯ Dans la mesure du possible, l'examen physique doit donc être enregistré en tant que diagnostic dans le DMI.

◯ Pour tous les patients de 12 ans et plus, est notée dans le DMI si le patient utilise des cigarettes, d'alcool et de substances.

◯ Si la section "Anamnèse familiale" est présente dans le DMI: enregistrez ici les informations sur les troubles qui surviennent chez les membres de la famille

◯ Si le patient n'a pas d'allergies connues ou d'antécédents de réactions indésirables, cela est noté de manière appropriée dans le dossier.

◯ Examen psychogériatrique : si une personne fragile est sélectionnée, cela est noté dans le DMI dans les sections convenues concernant la fragilité.

◯ Les données sociales sont enregistrées dans la section du fichier consacrée aux données sociales.

Si vous avez des suggestions de recommandations qui, selon vous, ne figurent pas dans la liste ci-dessus, vous pouvez les noter dans l'encadré ci-dessous.

Veuillez toujours indiquer clairement une recommandation et sa motivation.

Veuillez écrire votre réponse ici :

5. Identification du patient / Informations de contact

Dans quelle mesure les recommandations suivantes sont-elles pertinentes pour mesurer la qualité d'une bonne utilisation du DMI en médecine générale en ce qui concerne **l'identification du patient et l'information de contact** dans le DMI?

Indicateur / recommandation	Source	Année	Niveau de preuve
**RECOMMANDATION:** **Le service des urgences (hôpital) dans lequel le patient est connu doit être enregistré dans le DMI.**	**NHS**	**2023**	**Pas de gradation**

Votre évaluation:

*

Veuillez sélectionner une seule des propositions suivantes :

- ◯ 1 (Médiocre)
- ◯ 2
- ◯ 3
- ◯ 4
- ◯ 5
- ◯ 6
- ◯ 7
- ◯ 8
- ◯ 9 (Excellent)
- ◯ Non évaluable

Evaluation basée sur:

Veuillez choisir toutes les réponses qui conviennent :

- [ ] L’extractibilité de le DMI
- [ ] Recommandation sur la pertinence

Indicateur / recommandation	Source	Année	Niveau de preuve
**RECOMMANDATION:** **Le DMI doit indiquer si le patient est hospitalisé ou non.**	**NHS**	**2023**	**Pas de gradation**

Votre évaluation:

*

Veuillez sélectionner une seule des propositions suivantes :

- ○ 1 (Médiocre)
- ○ 2
- ○ 3
- ○ 4
- ○ 5
- ○ 6
- ○ 7
- ○ 8
- ○ 9 (Excellent)
- ○ Non évaluable

Evaluation basée sur:

Veuillez choisir toutes les réponses qui conviennent :

- [ ] L’extractibilité de le DMI
- [ ] Recommandation sur la pertinence

Indicateur / recommandation	Source	Année	Niveau de preuve
**RECOMMANDATION:** **Les coordonnées des personnes de contact, des aidants informels et des personnes désignées comme représentants sont consignées dans le dossier.**	**ADEP HIQA SSMG NHS**	**2019 2018 ? 2023**	**Pas de gradation**

Votre évaluation:

*

Veuillez sélectionner une seule des propositions suivantes :

- ◯ 1 (Médiocre)
- ◯ 2
- ◯ 3
- ◯ 4
- ◯ 5
- ◯ 6
- ◯ 7
- ◯ 8
- ◯ 9 (Excellent)
- ◯ Non évaluable

Evaluation basée sur:

Veuillez choisir toutes les réponses qui conviennent :

- ☐ L'extractibilité de le DMI
- ☐ Recommandation sur la pertinence

Indicateur / recommandation	Source	Année	Niveau de preuve
**RECOMMANDATION:** **Le DMI doit contenir des informations sur l'équipe soignante qui suit le patient.**	**NHS**	**2023**	**Pas de gradation**

Votre évaluation:

*

Veuillez sélectionner une seule des propositions suivantes :

- ◯ 1 (Médiocre)
- ◯ 2
- ◯ 3
- ◯ 4
- ◯ 5
- ◯ 6
- ◯ 7
- ◯ 8
- ◯ 9 (Excellent)
- ◯ Non évaluable

Evaluation basée sur:

Veuillez choisir toutes les réponses qui conviennent :

- ☐ L’extractibilité de le DMI
- ☐ Recommandation sur la pertinence

Indicateur / recommandation	Source	Année	Niveau de preuve
**RECOMMANDATION:** **Le DMI doit contenir des informations personnelles pertinentes pour le prestataire de soins de santé, à savoir : l'adresse du patient, son employeur, son numéro de téléphone au domicile et au travail et sa situation de famille.**	**NCQA**	**2018**	**Pas de gradation**

Votre évaluation:

*

Veuillez sélectionner une seule des propositions suivantes :

- ◯ 1 (Médiocre)
- ◯ 2
- ◯ 3
- ◯ 4
- ◯ 5
- ◯ 6
- ◯ 7
- ◯ 8
- ◯ 9 (Excellent)
- ◯ Non évaluable

Evaluation basée sur:

Veuillez choisir toutes les réponses qui conviennent :

- ☐ L’extractibilité de le DMI
- ☐ Recommandation sur la pertinence

Trois recommandations principales :

Quelles recommandations concernant "identification du patient" vous semblent les plus appropriées pour mesurer la qualité d'une bonne utilisation du DMI en médecine générale ?

1.

*

Veuillez sélectionner une seule des propositions suivantes :

- ◯ Le service des urgences (hôpital) dans lequel le patient est connu doit être enregistré dans le DMI.
- ◯ Le DMI doit indiquer si le patient est hospitalisé ou non.
- ◯ Les coordonnées des personnes de contact, des aidants informels et des personnes désignées comme représentants sont consignées dans le dossier.
- ◯ Le DMI doit contenir des informations sur l'équipe soignante qui suit le patient.
- ◯ Le DMI doit contenir des informations personnelles pertinentes pour le prestataire de soins de santé, à savoir : l'adresse du patient, son employeur, son numéro de téléphone au domicile et au travail et sa situation de famille.

2.

*

Veuillez sélectionner une seule des propositions suivantes :

- ◯ Le service des urgences (hôpital) dans lequel le patient est connu doit être enregistré dans le DMI.
- ◯ Le DMI doit indiquer si le patient est hospitalisé ou non.
- ◯ Les coordonnées des personnes de contact, des aidants informels et des personnes désignées comme représentants sont consignées dans le dossier.
- ◯ Le DMI doit contenir des informations sur l'équipe soignante qui suit le patient.
- ◯ Le DMI doit contenir des informations personnelles pertinentes pour le prestataire de soins de santé, à savoir : l'adresse du patient, son employeur, son numéro de téléphone au domicile et au travail et sa situation de famille.

3.

*

Veuillez sélectionner une seule des propositions suivantes :

- ◯ Le service des urgences (hôpital) dans lequel le patient est connu doit être enregistré dans le DMI.
- ◯ Le DMI doit indiquer si le patient est hospitalisé ou non.
- ◯ Les coordonnées des personnes de contact, des aidants informels et des personnes désignées comme représentants sont consignées dans le dossier.
- ◯ Le DMI doit contenir des informations sur l'équipe soignante qui suit le patient.
- ◯ Le DMI doit contenir des informations personnelles pertinentes pour le prestataire de soins de santé, à savoir : l'adresse du patient, son employeur, son numéro de téléphone au domicile et au travail et sa situation de famille.

Si vous avez des suggestions de recommandations qui, selon vous, ne figurent pas dans la liste ci-dessus, vous pouvez les noter dans l'encadré ci-dessous.

Veuillez toujours indiquer clairement une recommandation et sa motivation.

Veuillez écrire votre réponse ici :

6. Statut vaccinal

Dans quelle mesure les recommandations suivantes sont-elles pertinentes pour mesurer la qualité d'une bonne utilisation du DMI en médecine générale en ce qui concerne **le statut vaccinal du patient**?

Indicateur / recommandation	Source	Année	Niveau de preuve
**INDICATEUR:**<br>**Pourcentage de la population âgée actuellement de 7 ans ayant reçu les vaccinations primaires recommandées pour les enfants. (Calendrier de vaccination | vaccination-info)**	**CIHI**	**2016**	**Pas de gradation**
**RECOMMANDATION LIEE:**<br>**Le carnet de vaccination (pour les enfants) est à jour ou un historique approprié a été établi dans le dossier médical (pour les adultes).**	**NCQA, NHS, Domus Medica, SSMG, HIQA**	**2018, 2023, 2004, ?, 2020**	**Pas de gradation**

Votre évaluation:

*

Veuillez sélectionner une seule des propositions suivantes :

◯ 1 (Médiocre)
◯ 2
◯ 3
◯ 4
◯ 5
◯ 6
◯ 7
◯ 8
◯ 9 (Excellent)
◯ Non évaluable

Evaluation basée sur:

Veuillez choisir toutes les réponses qui conviennent :

☐ L’extractibilité de le DMI
☐ Recommandation sur la pertinence

Indicateur / recommandation	Source	Année	Niveau de preuve
**INDICATEUR:**<br>**Pourcentage des patients âgés de 65 ans et plus qui ont été vaccinés contre la grippe.**	**CIHI**	**2016**	**Pas de gradation**
**RECOMMANDATION LIEE:**<br>**Le carnet de vaccination (pour les enfants) est à jour ou un historique approprié a été établi dans le dossier médical (pour les adultes).**	**NCQA, NHS, Domus Medica, SSMG, HIQA**	**2018, 2023, 2004, ?, 2020**	**Pas de gradation**

Votre évaluation:

*

Veuillez sélectionner une seule des propositions suivantes :

- ◯ 1 (Médiocre)
- ◯ 2
- ◯ 3
- ◯ 4
- ◯ 5
- ◯ 6
- ◯ 7
- ◯ 8
- ◯ 9 (Excellent)
- ◯ Non évaluable

Evaluation basée sur:

Veuillez choisir toutes les réponses qui conviennent :

- ☐ L'extractibilité de le DMI
- ☐ Recommandation sur la pertinence

Indicateur / Recommandation	Source	Année	Niveau de preuve
**RECOMMANDATION:** **Le DMI doit indiquer que le patient n'a pas été vacciné.**	**HIQA**	**2018**	**/**

Votre évaluation:

*

Veuillez sélectionner une seule des propositions suivantes :

- ○ 1 (Médiocre)
- ○ 2
- ○ 3
- ○ 4
- ○ 5
- ○ 6
- ○ 7
- ○ 8
- ○ 9 (Excellent)
- ○ Non évaluable

Evaluation basée sur:

Veuillez choisir toutes les réponses qui conviennent :

- ☐ L’extractibilité de le DMI
- ☐ Recommandation sur la pertinence

Indicator / recommandation	Source	Année	Niveau de preuve
**RECOMMANDATION:** **Le DMI doit contenir la date d'administration de chaque vaccin.**	**HIQA**	**2018**	**/**

Votre évaluation:

*

Veuillez sélectionner une seule des propositions suivantes :

- ◯ 1 (Médiocre)
- ◯ 2
- ◯ 3
- ◯ 4
- ◯ 5
- ◯ 6
- ◯ 7
- ◯ 8
- ◯ 9 (Excellent)
- ◯ Non évaluable

Evaluation basée sur:

Veuillez choisir toutes les réponses qui conviennent :

- ☐ L’extractibilité de le DMI
- ☐ Recommandation sur la pertinence

Deux recommandations principales :

Quelles recommandations concernant "le statut vaccinal" vous semblent les plus appropriées pour mesurer la qualité d'une bonne utilisation du DMI en médecine générale ?

1.

*

Veuillez sélectionner une seule des propositions suivantes :

◯ Pourcentage de la population âgée actuellement de 7 ans ayant reçu les vaccinations primaires recommandées pour les enfants.

◯ Pourcentage des patients âgés de 65 ans et plus qui ont été vaccinés contre la grippe.

◯ Le DMI doit indiquer que le patient n'a pas été vacciné.

◯ Le DMI doit contenir la date d'administration de chaque vaccin.

2.

*

Veuillez sélectionner une seule des propositions suivantes :

◯ Pourcentage de la population âgée actuellement de 7 ans ayant reçu les vaccinations primaires recommandées pour les enfants.

◯ Pourcentage des patients âgés de 65 ans et plus qui ont été vaccinés contre la grippe.

◯ Le DMI doit indiquer que le patient n'a pas été vacciné.

◯ Le DMI doit contenir la date d'administration de chaque vaccin.

Si vous avez des suggestions de recommandations qui, selon vous, ne figurent pas dans la liste ci-dessus, vous pouvez les noter dans l'encadré ci-dessous.

Veuillez toujours indiquer clairement une recommandation et sa motivation.

Veuillez écrire votre réponse ici :

7. Volontés du patient

Dans quelle mesure les recommandations suivantes sont-elles pertinentes pour mesurer la qualité d'une bonne utilisation du DMI en médecine générale en ce qui concerne l'enregistrement des **volontés du patient** dans le DMI?

Indicateur / recommandation	Source	Année	Niveau de preuve
**RECOMMANDATION:** **Les rapports sur les conversations des patients concernant leurs souhaits en matière de traitements et de fin de vie sont principalement enregistrés dans les rapports SOEP dans un sous-contact "Conversation sur les souhaits en matière de fin de vie/traitement" (ICPC A20, version 2018).**	**ADEPD, NHS, HASP**	**2019, 2023, 2018**	**Pas de gradation**

Votre évaluation:

*

Veuillez sélectionner une seule des propositions suivantes :

- ◯ 1 (Médiocre)
- ◯ 2
- ◯ 3
- ◯ 4
- ◯ 5
- ◯ 6
- ◯ 7
- ◯ 8
- ◯ 9 (Excellent)
- ◯ Non évaluable

Evaluation basée sur:

Veuillez choisir toutes les réponses qui conviennent :

- ☐ L’extractibilité de le DMI
- ☐ Recommandation sur la pertinence

Indicateur / recommandation	Source	Année	Niveau de preuve
**RECOMMANDATION:** **Les décisions finales sur l'opportunité de traiter ou non dans des situations spécifiques sont enregistrées dans la section du dossier "Limites de traitement".** **Ces limites de traitement sont les suivantes: enregistrement de la réanimation cardio-pulmonaire (RCP) ; hospitalisation, admission en unité de soins intensifs, ventilation artificielle, administration d'un produit sanguin, autre traitement (en texte libre) ou administration d'antibiotiques.**	**ADEPD**	**2019**	**Pas de gradation**

Votre évaluation:

*****

Veuillez sélectionner une seule des propositions suivantes :

- ◯ 1 (Médiocre)
- ◯ 2
- ◯ 3
- ◯ 4
- ◯ 5
- ◯ 6
- ◯ 7
- ◯ 8
- ◯ 9 (Excellent)
- ◯ Non évaluable

Evaluation basée sur:

Veuillez choisir toutes les réponses qui conviennent :

- [ ] L'extractibilité de le DMI
- [ ] Recommandation sur la pertinence

Indicateur / recommandation	Source	Année	Niveau de preuve
**RECOMMANDATION:** **Les déclarations écrites de volonté, par exemple une déclaration d'euthanasie ou de non-réanimation, proposées par le patient au médecin généraliste sont ajoutées au DMI en tant que correspondance.**	**ADEPD**	**2019**	**Pas de gradation**

Votre évaluation:

*

Veuillez sélectionner une seule des propositions suivantes :

- ○ 1 (Médiocre)
- ○ 2
- ○ 3
- ○ 4
- ○ 5
- ○ 6
- ○ 7
- ○ 8
- ○ 9 (Excellent)
- ○ Non évaluable

Evaluation basée sur:

Veuillez choisir toutes les réponses qui conviennent :

- ☐ L’extractibilité de le DMI
- ☐ Recommandation sur la pertinence

Indicateur / recommandation	Source	Année	Niveau de preuve
**RECOMMANDATION:**<br><br>**Les objectifs des soins centrés sur la personne sont-ils consignés dans le dossier ? Dans les soins centrés sur la personne, les objectifs du patient sont au centre des préoccupations. Le patient détermine les objectifs des soins avec ses prestataires de soins de santé. Ces objectifs ne sont souvent pas de nature médicale, mais présentent des similitudes.**<br><br>**Par exemple, un patient atteint de BPCO souhaite pouvoir se promener tous les jours à l'extérieur et discuter avec ses voisins. Une adaptation adéquate de la BPCO, combinée à un entraînement à la marche et à la respiration, peut rendre l'objectif fixé réalisable. Cela nécessite une coordination entre le médecin généraliste (définition de la BPCO), les poh-somatics (contrôles réguliers de la BPCO) et un physiothérapeute spécialisé (entraînement à la marche et à la respiration). C'est indique clairement qui fait quoi et qui est responsable de quoi.**	**ADEPD**	**2019**	**Pas de gradation**

Votre évaluation:

*****

Veuillez sélectionner une seule des propositions suivantes :

◯ 1 (Médiocre)

◯ 2

- ◯ 3
- ◯ 4
- ◯ 5
- ◯ 6
- ◯ 7
- ◯ 8
- ◯ 9 (Excellent)
- ◯ Non évaluable

Evaluation basée sur:

Veuillez choisir toutes les réponses qui conviennent :

- ☐ L’extractibilité de le DMI
- ☐ Recommandation sur la pertinence

Indicateur / recommandation	Source	Année	Niveau de preuve
**RECOMMANDATION:**<br>**Le DMI doit contenir des informations sur les voies de dépistage applicables à chaque patient ou sur les souhaits du patient concernant ce dépistage.**<br>**En Belgique, le dépistage de la population concerne le cancer du sein, le cancer du col de l'utérus, le cancer du côlon et les maladies congénitales (Soorten bevolkingsonderzoeken | Bevolkingsonderzoek).**<br>**Remarque : nous parlons ici de l'acceptation/du rejet des plans de soins et non de la présence de plans individuels (comme le recommendation du topic 4).**	**ADEPD**	**2019**	**Pas de gradation**

Votre évaluation:

*

Veuillez sélectionner une seule des propositions suivantes :

- ◯ 1 (Médiocre)
- ◯ 2
- ◯ 3
- ◯ 4
- ◯ 5
- ◯ 6
- ◯ 7
- ◯ 8
- ◯ 9 (Excellent)
- ◯ Non évaluable

Evaluation basée sur:

Veuillez choisir toutes les réponses qui conviennent :

- [ ] L’extractibilité de le DMI
- [ ] Recommandation sur la pertinence

Trois recommandations principales :

Quelles recommandations concernant "les volontés du patient" vous semblent les plus appropriées pour mesurer la qualité d'une bonne utilisation du DMI en médecine générale ?

1.

*

Veuillez sélectionner une seule des propositions suivantes :

◯ Les rapports sur les conversations des patients concernant leurs souhaits en matière de traitements et de fin de vie sont principalement enregistrés dans les rapports SOEP dans un sous-contact "Conversation sur les souhaits en matière de fin de vie/traitement" (ICPC A20, version 2018).

◯ Les décisions finales sur l'opportunité de traiter ou non dans des situations spécifiques sont enregistrées dans la section du dossier "Limites de traitement".

◯ Les déclarations écrites de volonté, par exemple une déclaration d'euthanasie ou de non-réanimation, proposées par le patient au médecin généraliste sont ajoutées au DMI en tant que correspondance.

◯ Les objectifs des soins centrés sur la personne sont-ils consignés dans le dossier ?

◯ Le DMI doit contenir des informations sur les voies de dépistage applicables à chaque patient ou sur les souhaits du patient concernant ce dépistage.

2.

*

Veuillez sélectionner une seule des propositions suivantes :

◯ Les rapports sur les conversations des patients concernant leurs souhaits en matière de traitements et de fin de vie sont principalement enregistrés dans les rapports SOEP dans un sous-contact "Conversation sur les souhaits en matière de fin de vie/traitement" (ICPC A20, version 2018).

◯ Les décisions finales sur l'opportunité de traiter ou non dans des situations spécifiques sont enregistrées dans la section du dossier "Limites de traitement".

◯ Les déclarations écrites de volonté, par exemple une déclaration d'euthanasie ou de non-réanimation, proposées par le patient au médecin généraliste sont ajoutées au DMI en tant que correspondance.

◯ Les objectifs des soins centrés sur la personne sont-ils consignés dans le dossier ?

◯ Le DMI doit contenir des informations sur les voies de dépistage applicables à chaque patient ou sur les souhaits du patient concernant ce dépistage.

3.

*

Veuillez sélectionner une seule des propositions suivantes :

- ◯ Les rapports sur les conversations des patients concernant leurs souhaits en matière de traitements et de fin de vie sont principalement enregistrés dans les rapports SOEP dans un sous-contact "Conversation sur les souhaits en matière de fin de vie/traitement" (ICPC A20, version 2018).
- ◯ Les décisions finales sur l'opportunité de traiter ou non dans des situations spécifiques sont enregistrées dans la section du dossier "Limites de traitement".
- ◯ Les déclarations écrites de volonté, par exemple une déclaration d'euthanasie ou de non-réanimation, proposées par le patient au médecin généraliste sont ajoutées au DMI en tant que correspondance.
- ◯ Les objectifs des soins centrés sur la personne sont-ils consignés dans le dossier ?
- ◯ Le DMI doit contenir des informations sur les voies de dépistage applicables à chaque patient ou sur les souhaits du patient concernant ce dépistage.

Si vous avez des suggestions de recommandations qui, selon vous, ne figurent pas dans la liste ci-dessus, vous pouvez les noter dans l'encadré ci-dessous.

Veuillez toujours indiquer clairement une recommandation et sa motivation.

Veuillez écrire votre réponse ici :

Le fin

Merci d'avoir participé à la première partie de l'enquête visant à créer un ensemble d'indicateurs sur l'utilisation correcte du dossier électronique du patient par les médecins généralistes. Nous espérons pouvoir vous attendre le 22 octobre 2024 à 20h00 pour la table ronde. Vous recevrez un autre courriel de rappel à cet effet.

Date	Quoi?	Comment?	Durée?
30 septembre 2024 23:59	Date limite pour remplir le questionnaire	Online enquête	30 min
5 novembre 2024 20h00	Réunion de consensus	Online	120 min
30 novembre 2024 23:59	Date limite de lecture de la liste finale des indicateurs	Courriel	15 min

Si vous avez des suggestions de recommandations qui, selon vous, ne figurent pas dans la liste ci-dessus, vous pouvez les noter dans l'encadré ci-dessous.

Veuillez toujours indiquer clairement une recommandation et sa motivation.

Veuillez écrire votre réponse ici :

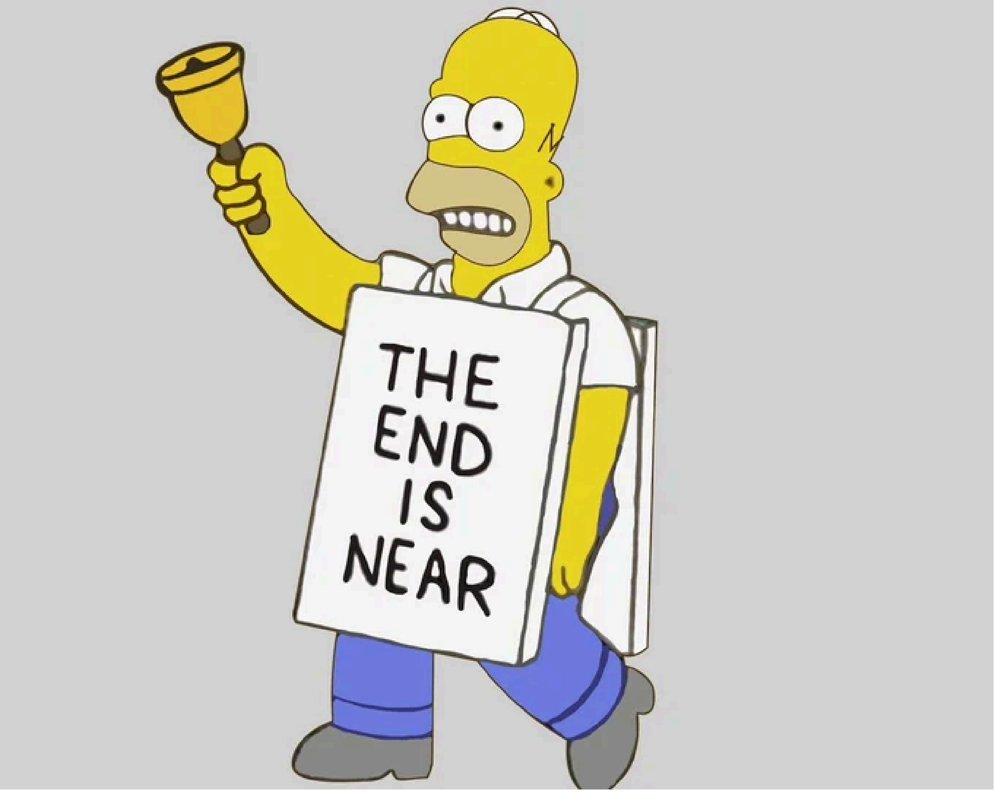

16/11/2024 – 17:01

Envoyer votre questionnaire.

Merci d'avoir complété ce questionnaire.
