# Supplementary material for: Development of Quality Indicators for the Correct Use of Electronic Medical Records in Primary Care: Modified Delphi Study
Source: JMIR Med Inform. 2026 Jan 19;14:e80057. doi: 10.2196/80057 (PMC12865340; doi:10.2196/80057)
Supplement: Multimedia Appendix 4 [file medinform_v14i1e80057_app4.pdf]

Development of quality indicators for the proper use of electronic patient records in general practice

Dear Sir, Dear
Madam,

First of all, we would like to thank you for your willingness to participate in our expert panel. The research in which the expert panel plays an important role is being conducted by the Academic Centre for General Practice at KU Leuven and aims to develop a selection of quality indicators for the proper use of electronic medical records in general practice. A specific requirement for indicator selection is that they must be directly extractable from the Electronic Medical Record (EMR). The ultimate goal is to use the quality indicators to provide automatic feedback to GPs on the quality of their patients' medical records. In this way, we aim to improve the quality of care.

You have been invited to participate in the selection of quality indicators for the proper use of electronic patient records in general practice because of your knowledge and expertise. As stated in the invitation, we are using the Rand modified Delphi method to select quality indicators for this study. This is a consensus method that is divided into three steps. In the first step, we ask you to score a list of recommendations according to their importance, usefulness and relevance in primary care. The aim is to use your help to draw up a short and powerful set of recommendations which, after completing the entire procedure, will ultimately be translated into a core set of quality indicators. Completing the questionnaire takes approximately 30 minutes. We would like to receive your completed questionnaire before **23:59** on **30 September 2024.**

In the second step, the results of the completed questionnaires will be analysed. During a face-to-face panel discussion, we will try to reach a consensus on the acceptance, rejection or reformulation of the potential indicators. This discussion, the date of which will be announced later, will take approximately two hours.

In the third and final step, the aim is to submit the final list of indicators in writing to all the experts on the panel in order to obtain final approval. This step will take no longer than 15 minutes and the date will also be announced later.

If you have any questions or comments, please contact us by e-mail (rico.paridaens@outlook.com).

Yours sincerely,

The project group consisting of:

Dr Rico Paridaens, general practitioner, KU Leuven Professor Dr Bert Vaes, general practitioner, KU Leuven Dr Steve Van den Bulck, general practitioner, KU Leuven

There are 148 questions in this survey.

General information about the study

General objectives of the research

The research, in which the expert panel plays an important role, is being conducted by the Academic Centre for General Practice at KU Leuven and aims to develop a selection of quality indicators for the proper use of electronic medical records in general practice. A specific requirement for indicator selection is that they can be extracted directly from the Electronic Medical Record (EMR). The ultimate goal is to use the quality indicators to provide automatic feedback to GPs about the quality of their patients' medical records. In this way, we aim to improve the quality of care.

Information about the processing of your personal data

As part of your participation in this study, personal data about you will be collected and processed. This processing will be carried out in accordance with the General Data Protection Regulation (GDPR). The following categories of personal data will be processed during this study: name, age, gender, position, workplace: hospital/practice and department for reporting in the consensus report, audio recording during the face-to-face panel discussion for processing into the consensus report.

Use of your personal data

Only personal data that is necessary for the purposes of this study will be collected and processed. Your data will be pseudonymised for the purposes of this study. This means that data that could identify you, such as name, age, gender, position, workplace: hospital/practice, will be disconnected from the other research data and replaced by a unique, random code. In this way, it will no longer be immediately apparent which data originates from which specific person. Only the researcher can link the data back to a specific person using the unique code. However, this will only happen in exceptional cases, for example if you exercise your right to access or rectify your data. For the quality of the research, it is important that we are able to mention your name, age, gender, position, workplace: hospital/practice as a participant in the study. You will not be identified in the output of this research, such as publications. However, the results will never refer directly to a person in the output of this research and will always be stated as a general conclusion.

The legal basis for the processing of your data is the public interest. This means that the research will lead to an increase in knowledge and insight that will benefit society (directly or indirectly). Discontinuation of participation in the study means that the data collected previously can still be legally included in the study and does not have to be deleted by KU Leuven.

Your data will be stored by the researchers for 10 years after the end of the research at a secure storage location at KU Leuven.

Your rights

You always have the right to request more information about the use of your data. In addition, you can invoke the right to access and the right to correct (rectify) your data, insofar as these rights do not render the purposes of the research impossible or seriously impede them.

If you wish to exercise any of these rights, please contact the researchers using the contact details at the top of this letter.

Reuse of your data

It is possible that your pseudonymised data may be reused for scientific research by:

- researchers and/or academic partners who collaborate with or receive data from KU Leuven (including, under certain conditions, master's or doctoral students),

Making data available is essential for validating research results and advancing scientific knowledge.

In the event of reuse of your data, the necessary contractual agreements will always be made to protect personal data and to determine the responsibilities and liabilities of the parties in accordance with the relevant legislation.

In the context of reuse, your personal data may be transferred outside the European Economic Area, provided that appropriate measures have been taken to protect your personal data in accordance with the relevant legislation. In particular, this data will in principle only be transferred in pseudonymised form, and all parties involved in the research will be obliged to respect the confidentiality of the personal data.

You will be informed transparently about any reuse of your data. This will be done by email.

Any reuse will always be in accordance with the applicable legislation and KU Leuven's policy on the matter. This policy includes, among other things, that an independent body will monitor the protection of personal data and your rights.

Please note that your pseudonymised data may also be made available on certain data platforms for further scientific research, with strictly secure and controlled access. This may happen, for example, in the context of publications about results. The data platform provides an access policy and undertakes to regulate access to data in accordance with applicable legislation.

Contact details

KU Leuven acts as the controller in the context of this research. More specifically, only researchers Rico Paridaens, Bert Vaes and Steve Van den Bulck will have access to your personal data. If you have specific questions about this research, including the processing of your personal data, please contact them.

For further questions and concerns about the processing of your personal data, please contact the data protection officer for scientific research at KU Leuven (dpo@kuleuven.be). Please clarify which research project you are referring to by stating the title and the names of the researchers.

If, after contacting the data protection officer, you wish to lodge a complaint about how your information is being handled, you can contact the Belgian Data Protection Authority (www.gegevensbeschermingsautoriteit.be).

Specific information about the research

From recommendation to indicator

To draw up the list of recommendations in this document, we used national and international guidelines (see references). We started by compiling a list containing all the recommendations for the proper use of electronic medical records in general practice from all the sources consulted. However, it would have been impossible to use such an extensive list to select relevant indicators. Therefore, in the next step, we selected those recommendations that were useful in primary care and that could potentially be extracted from the EMD. These are two important conditions in the context of our study. We also checked whether the recommendations were formulated in a 'SMART' manner, i.e. Specific, Measurable, Achievable, Relevant and Time-bound. The result is a basic set of recommendations as presented in this document.

At this stage, you are asked to score all recommendations according to the extent to which you consider the specific recommendations to be important and extractable from the EMD in order to measure the quality of the proper use of the electronic medical record in general practice. For further explanation, see the instructions below.

Instructions for completion

Firstly, we request that you complete the attached informed consent form. This questionnaire contains 50 indicators and recommendations, divided into the following categories:

1. Completeness and adequacy of the problem list (16 indicators/recommendations);
2. Structured recording in EMD (5 indicators/recommendations);
3. Completeness and timeliness of medication overview (5 indicators/recommendations);
4. Risk factors/medication monitoring (10 indicators/recommendations);
5. Patient identification/contact information (5 indicators/recommendations);
6. Vaccination status (4 indicators/recommendations); and
7. Patient's wishes (5 indicators/recommendations).

We ask you to assess each recommendation on the extent to which it can be extracted from the EMD and is important for measuring the quality of the electronic medical record in general practice using a 9-point Likert scale. This is a scale from 1 to 9, with 1 being the lowest score (poor measure of quality) and 9 being the highest score (excellent measure of quality).

To assess whether a recommendation is eligible for measuring the quality of care, you can use the following criteria:

- The recommendation is relevant to the primary care process. The recommendation improves the patient's health.
- The recommendation improves the patient's quality of life.
- The recommendation improves the effectiveness of care for the patient.
- The recommendation can be translated into an indicator that can be automatically extracted from the EMD.

In addition to a score according to the 9-point Likert scale, we also ask you, after assessing all recommendations from one category, to draw up a top 5 of recommendations per category based on their suitability for measuring the quality of care. For each category, we offer you the opportunity to add to this top 5.

We offer you the opportunity to formulate your own comments, add to the recommendations or write your own recommendations at the end of the questionnaire.

Sources used

The sources consulted for drawing up the list of recommendations are listed below. The abbreviations used in the questionnaire (for the source), the year of publication or last update and the country of origin are also mentioned.

- Domus Medica: Verdonck P, Strobbe J, Steenackers J et al. The electronic medical record. Huisarts Nu March 2004; 33(2).
- SSMG: Société Scientifique de Médecine Générale (SSMG). Organisation de la pratique. [cited 2024 Jan 27]; Available from: https://www.ssmg.be/organisation-pratique/
- NCQA: National Committee for Quality Assurance (NCQA). Guidelines for Medical Record Documentation. 2018 [cited 2024 Jan 27]; Available from: https://www.ncqa.org/wp-content/uploads/2018/07/20180110_Guidelines_Medical_Record_Documentation.pdf
- ADEPD: Duineveld B, Kole HM, Van Werven H. NHG Guideline Adequate record keeping with the electronic patient record (ADEPD). 2019 [cited 2024 Jan 27]; Available from: https://www.nhg.org/praktijkvoering/informatisering/richtlijn-adequate-

record-keeping-epd/

- HASP: Federation of Medical Specialists (FMS), Dutch College of General Practitioners (NHG). Guideline on information exchange between general practitioners and medical specialists (HASP. 2017 [cited 2024 Jan 27]; Available from: https://www.nhg.org/praktijkvoering/gegevensuitwisseling/gegevensuitwisseling-huisarts-specialist-hasp/
- NHS: National Health Service (NHS). Summary Care Records. Version 1.3, 19 April 2023. [cited 27 January 2024]; Available from: https://www.england.nhs.uk/long-read/summary-care-records-scr/
- HIQA: Health Information and Quality Authority. Recommendations on the implementation of a national electronic patient summary in Ireland. 2020 [cited 27 January 2024]; Available from: https://www.hiqa.ie/reports-and-publications/health-information/recommendations-implementation-national-electronic
- Hiddema-van der Wal: Hiddema-van der Wal A, van der Werf GTh, Meyboom-de Jong B. Which ICPC codes do GPs want to have automatically added to the problem list? Huisarts en Wetenschap 46(10) September 2003. Pages 539-543
- EPD-scan-h: Lea Jabaaij, Robert Verheij, Khing Njoo, Henk van den Hoogen, Waling Tiersma, Herman Levelink. Measuring the quality of registration in general practitioners' electronic patient records using the EPD-scan-h (EPD-scan-h). ISBN 978-90-6905-896-2. 2008
- Hamade et al: Hamade N, Terry A, Malvankar-Mehta M. Interventions to improve the use of EMRs in primary health care: a systematic review and meta-analysis. BMJ Health Care Inform. 2019 May;26(1):e000023.
- De Lusignan et al: De Lusignan S. Does Feedback Improve the Quality of Computerised Medical Records in Primary Care? Journal of the American Medical Informatics Association. 1 July 2002;9(4):395–401.
- CIHI: Ottawa - Ontario : Canadian Institute for Health Information. Pan-Canadian primary health care indicator - update report. 2016. Canada. ISBN: 978-1-77109-146-6

Informed Consent

Title of the study:
Development of quality indicators for the proper use of electronic patient records in general practice

Name + contact details of supervisor and researcher(s):

- Researcher: Rico Paridaens,rico.paridaens@outlook.com , 0470 83 39 09
- Supervisor: Bert Vaes,bert.vaes@kuleuven.be , 0474 33 05 13, Department of Public Health and Primary Care, Academic Centre for General Practice

Aim and methodology of the research:
INTEGO aims to establish quality indicators for the most important clinical pictures in general practice and, more generally, for the use of the EPD by general practitioners, using RAND-Modified Delphi procedures. This is a procedure in which each participant systematically goes through a number of steps to ultimately arrive at a consensus report with recommendations that is supported by all participants. For this research, we will determine quality indicators for:

During the study, you will go through the following steps:

1. Online questionnaire: You will be asked to score a list of potential quality indicators/recommendations on their ability to measure the quality of the subject using a Likert scale from 1 (= lowest score) to 9 (= highest score). You will receive a feedback report on the first round with your personal score, the median score of all participants and, based on this, the potential of the quality indicator/recommendation.
2. Face-to-face panel discussion: The moderate recommendations (questionable results) are discussed, as are the newly introduced indicators. The strong and weak indicators are only discussed if comments have been made about them. Based on the conclusions of the face-to-face meeting, a set of recommendations is drawn up, which is submitted to all panel members for approval for a third and final assessment.

Duration of the experiment: +/- 30 min

I understand and agree to the

following:

- I understand what is expected of me during this study. I know that I will participate in the following trials or tests: Online survey and face-to-face panel discussion
- I am aware that there may be risks or inconveniences associated with my participation: Possible conflicts during the discussions of the face-to-face panel discussion
- I or others may benefit from this research in the following ways: These quality indicators can be used
-

to provide feedback to general practitioners in Belgium and other countries about the quality of patient care.

- My participation contributes to scientific research. I understand that I will not receive any further reward or compensation for my participation.
- I understand that my participation in this study is voluntary. I have the right to withdraw from the study at any time. I do not need to give a reason for doing so, and I understand that this will not result in any disadvantage to me.
- I am aware that recordings may be made of me during this study: audio recordings during the face-to-face panel discussion for the development of the consensus report. My personal data will be processed in accordance with the General Data Protection Regulation (GDPR).
- Only data that is strictly necessary for achieving the research objectives will be processed. My data will be treated confidentially throughout the research. The researchers will take measures to protect my privacy. For example, my personal data will be pseudonymised, which means that my data can no longer be linked to me without the use of additional information that is only accessible to the researchers. I understand that my pseudonymised data may be reused for other scientific research and possibly also in the context of education and academic lectures. More information about the processing of my personal data can be found in the attached information letter.

I would like to be kept informed of the results of this study. The researcher may contact me at the following email address:

Enter your answer here:

- **Although this study does not involve diagnostic screening, there is a small chance that the researchers may accidentally come across unforeseen research results that they deem necessary to inform me about (e.g. possible indications of medical or psychological problems). In that case, they may contact me via the above email address to inform me of this. If I do not wish to be informed, I will tick the box below.**

Select all options that apply to you:

I do not wish to be informed of any unexpected research findings.

☐

- **For further questions about the study, I know that after my participation I can contact:**
  Rico Paridaens (rico.paridaens@outlook.com)
- **This study has been reviewed and approved by the Social and Ethical Committee (SMEC) of KU Leuven (G-2024-8020, please quote this number in any communication about the study). For any complaints or other concerns regarding ethical aspects of this study, I can contact SMEC: smec@ kuleuven.be**
- **I know that I can contact the following if, after the investigation, I experience discomfort or difficulties as a result of the issues addressed in the investigation:**
  Rico Paridaens (rico.paridaens@outlook.com)

Personal information

I have read and understood the above information and have received answers to all my questions regarding this study. I agree to participate.

*****

Please select one of the following options:

◯ Agree

◯ Disagree

Surname and first name:
(This information will only be used to send the results of the first Delphi round and to send invitations to the panel discussion.) *

Enter your answer here:

Email address:
(This information will only be used to send you the results of the first Delphi round and to invite you to the panel discussion.) *

Enter your answer here:

Age: *

Enter your answer here:

Gender: *

If you select 'Other:', please explain your choice in the corresponding text box. Select one of the following options:

- ◯ Male
- ◯ Female
- ◯ Other

Workplace:
(e.g. hospital: specify)
*

Select one of the following options:

- ◯ Hospital
- ◯ General practice: solo/duo practice General
- ◯ practice: group practice

Please explain your answer here:

Position at workplace: *

Select one of the following options: General

- ◯ practitioner
- ◯ Other

Affiliation: *

Select one of the following options:

- ◯ Ghent University
- ◯ Hasselt University KU
- ◯ Leuven University of
- ◯ Antwerp
- ◯ Vrije Universiteit Brussel Université
- ◯ Liège
- ◯ Catholic University of Louvain
- ◯ Saint-Louis University - Brussels
- ◯ University of Namur
- ◯ University of Mons
- ◯ Free University of Brussels Other
- ◯
- ◯

Which EMD do you use? *

Select one of the following options:

- ◯ CareConnect
- ◯ HealthOne
- ◯ Medispring Daktari
- ◯ Other
- ◯

1. Completeness and adequacy of problem list

To what extent are the following recommendations relevant for measuring the quality of the proper use of the EMD in general practice in relation to the **completeness and adequacy of the** patient **problem list**?

Indicator / Recommendation	Source	Year	Level of evidence
**INDICATOR:**<br><br>**How many active care elements does a patient have on average on his/her list of care elements?**	**EPD scan-h**	**2009**	**No gradation**
**RELATED RECOMMENDATION:**<br><br>**Episode list: the EPD presents open episodes with flag, closed episodes with flag, and open episodes without flag in succession.**	**ADEPD, HIQA**	**2019 2020**	**No grading**

Your assessment:

*

Choose one of the following options:

- ◯ 1 (Poor)
- ◯ 2
- ◯ 3
- ◯ 4
- ◯ 5
- ◯ 6
- ◯ 7
- ◯ 8
- ◯ 9 (Excellent)
- ◯ Not assessable

Assessment based on:

Select all options that apply to you:

- ☐ EMD extractability
- ☐ Relevance of recommendation

Indicator / Recommendation		Source	Year	Level
of evidence **INDICATOR:** Percentage of problem status the label 'special attention value'?	EPD Nonegradation scan-h	2009 episodes indeed have		

Additional information: Problems requiring special attention are automatically placed at the top of the problem list so that they are clearly visible in the
EPD. Care elements with problem status are care elements with an ICPC code that qualify for automatic placement on the problem list because they are important to the care provider. These care elements are:

A12	allergy/allergic reaction neg.	N88	epilepsy all forms
A70	tuberculosis contraindicated [excl. R70]	N89	migraine
A79	malignant neoplasm (unknown primary site)	P15	chronic alcohol abuse
A85	drug reaction	P18	drug abuse
A90	congenital syndromes/multiple abnormalities	P21	overactive child/hyperkinetic syndrome
B72	Hodgkin's disease/other malignant lymphomas	P70	dementia (including senile/Alzheimer's)
B73	leukaemia	P71	other organic psychoses
B74	other malignant neoplastic diseases of blood/blood-forming organs	P72	schizophrenia, all forms
B76	Spleen rupture	P73	affective psychoses
B78	hereditary haemolytic anaemia	P74	anxiety disorder/pathological anxiety
B79	other specified disorders of blood/blood-forming organs	P76	depression
B81	pernicious/folic acid deficiency anaemia	P77	suicide attempt

B83	purpura/coagulation disorder/immune thrombosis	R70	tuberculosis tr. resp. [ex c contraindication A70]
D74	malignant neoplasm stomach	R78	acute bronchitis (>2x/year)
D75	malignant neoplasm of the colon/rectum	R82	c pleurisy all forms [ex tbc R70]
D76	malignant neoplasm of pancreas	R84	malignant neoplasm of bronchus/lung
D77	malignant neoplasm of the digestive tract, unspecified	R85	other malignant neoplasn resp.
D78	benign tumours of the gastrointestinal tract	R89	Congenital anomalies resp.
D81	congenital anomaly of the digestive tract	R91	chronic bronchitis/bronchiectasis
D85	duodenal ulcer	R95	emphysema/COPD
D86	other peptic ulcers	R96	asthma
D92	diverticulosis/diverticulitis	R97	hay fever/allergic rhinitis
D93	spastic colon/IBS	S77	malignant neoplasm of skin/subcutis
D94	ulcerative colitis/chronic enteritis	S87	constitutional eczema
D97	cirrhosis/other liver diseases neg.	S91	c psoriasis (including arthropathy)
D98	cholecystitis/cholelithiasis	T71	malignant neoplasm of thyroid gland
F81	other congenital anomalies of the eye	T72	benign neoplasm of thyroid gland
F92	cataract	T80	and. applied. endo. kl./metab.
F93	glaucoma	T81	r goitre/nodules thyroid [excl. T85]
F94	blindness/visual impairment (all forms)	T85	hyperthyroidism (with/without goitre)
H83	otosclerosis	T86	e hypothyroidism/myxoede ma
H84	presbyacusis	T90	diabetes mellitus

K73	congenital circulatory abnormality	T92	gout
K74	angina pectoris	T93	lipid metabolism disorder
K75	acute myocardial infarction	U71	urinary tract infections (>2x/year)
K76	and chronic ischaemic heart disease	U75	malignant neoplasm of kidney
K77	cardiac decompensation	U76	malignant neoplasm of the bladder
K78	atrial fibrillation/flutter	U77	other malignant neoplasms of urinary tract
K79	paroxysmal tachycardia	U85	congenital anomalies of urinary tract
K83	valvular disease, non-rheumatic/unspecified	U88	glomerulonephritis/nephrosis
K86	hypertension without organ damage	U95	urolithiasis (all forms/localised)
K87	hypertension with organ damage.	W13	female sterilisation/referral
K89	transient ischaemic attack/TIA	W72	malignant neoplasm related to pregnancy
K90	cerebrovascular accident (CVA) [excluding TIA]	W76	congenital abnormality of mother as pregnancy complication
K91	atherosclerosis [excluding coronary/cerebral]	W80	ectopic pregnancy
K92	other peripheral arterial diseases	W82	spontaneous abortion/abortion n.o.s.
K93	pulmonary embolism/pulmonary infarction	W92	complicated delivery of live birth
K94	thrombophlebitis/phlebothrombosis	W93	complicated delivery of stillborn infant
L75	femur fracture	X74	inflammation of the small pelvis/PID
L82	Congenital musculoskeletal disorders	X75	malignant neoplasm of the cervix uteri
L88	rheumatoid arthritis/related conditions	X76	malignant neoplasm of the breast (female)

L89	coxarthrosis	X77	and malignant neoplasms of the reproductive organs (female)
L95	osteoporosis	X78	benign neoplastic uterus/cervix uteri
N70	poliomyelitis/other enterovirus infection	X83	congenital abnormality of female genital organs
N71	meningitis/encephalitis	X87	vaginal/uterine prolapse
N72	tetanus	Y77	malignant neoplasms of the prostate gland
N74	malignant neoplasm nervous system	Y78	and. mal. neopl. gen.org/breast. (male)
N75	benign neoplasm nervous system	Y82	hypospadias
N85	congenital anomalies of the nervous system	Y83	cryptorchidism/undescended testicle
N86	multiple sclerosis	Y84	and. app. afw. gesl.o (male)
N87	Parkinsonism/Parkinson's disease	Y85	benign prostatic hyperplasia

Source: A Hiddema-van der Wal, GTh van der Werf, B Meyboom-de Jong, “Which ICPC codes do general practitioners want to have automatically added to the problem list?

Your opinion:

*

Choose one of the following options: ◯ 1 (Poor)

◯ 2

◯ 3

◯ 4

◯ 5

◯ 6

◯ 7

◯ 8

◯ 9 (Excellent)

◯ Not assessable

Rating based on:

Select all options that apply to you:

☐ EMD extractability Relevance of

☐ recommendation

Indicator / recommendation	Source	Year	Level of evidence
**INDICATOR:**<br>A number of medications are prescribed specifically for a particular disease. For patients with a prescription for this medication, a care element with that diagnosis must be present. The absence of this may indicate a lack of registration of this care element in the EPD. These conditions are:<br>• Thyroid disease<br>• Epilepsy<br>• Parkinson's disease<br>• Depression<br>• Cardiovascular disease<br>• Asthma/COPD<br>• Diabetes	EHR scan-h	2009	No grading

Your assessment:

*

Choose one of the following options:

- ◯ 1 (Poor)
- ◯ 2
- ◯ 3
- ◯ 4
- ◯ 5
- ◯ 6
- ◯ 7
- ◯ 8
- ◯ 9 (Excellent)
- ◯ Not assessable

Assessment based on:

Select all options that apply to you:

☐ EMD extractability Relevance of
☐ recommendation

Indicator / recommendation	Source	Year	Level of evidence
INDICATOR: Percentage of registered patients for whom there was no change in the EPD over the past 12 months.	De Lusignan et al	2002	No gradation

Your assessment:

*

Choose one of the following options:

- ◯ 1 (Poor)
- ◯ 2
- ◯ 3
- ◯ 4
- ◯ 5
- ◯ 6
- ◯ 7
- ◯ 8
- ◯ 9 (Excellent)
- ◯ Not assessable

Assessment based on:

Select all options that apply to you:

☐ EMD extractability Relevance of
☐ recommendation

Indicator / recommendation	Source	Year	Evidence level
**INDICATOR:** **Percentage of the patient population, aged 18 years and older, with chronic health conditions who received at least one of the following types of self-management support from their primary care provider: Provided with a treatment plan; Encouraged to use self-help groups or programmes**	CIHI	2016	No gradation

Your rating:

*

Choose one of the following options:

- ◯ 1 (Poor)
- ◯ 2
- ◯ 3
- ◯ 4
- ◯ 5
- ◯ 6
- ◯ 7
- ◯ 8
- ◯ 9 (Excellent)
- ◯ Not assessable

Assessment based on:

Select all options that apply to you:

- ☐ EMD extractability Relevance of
- ☐ recommendation

Indicator / recommendation	Source	Year	Level of evidence
**INDICATOR:**<br><br>**What percentage of episodes on the episode list have a correct ICPC code?**<br><br>**ICPC code to be further divided into:**<br><br>• **Correct usage: Complaints in the range 01 to 29 and diagnoses in the range 70 to 99. This category also includes A44 (vaccination), R44 (influenza vaccination)**<br>• **and X37 (cervical smear screening). Possible incorrect use: A97 (no disease) or A99 (other generalised or unspecified disease) (as flight code);**<br>• **Unauthorised or no ICPC: No ICPC or an unauthorised code (range 30-69), with the exception of A44 (preventive vaccination or medication), R44 (influenza vaccination) and X37 (cervical smear screening).**	**EPD scan-h**	**2009**	**/**

Your opinion:

*

Choose one of the following options: 1

◯ (Poor)

- ○ 2
- ○ 3
- ○ 4
- ○ 5
- ○ 6
- ○ 7
- ○ 8
- ○ 9 (Excellent)
- ○ Not assessable

Assessment based on:

Select all options that apply to you:

- ☐ EMD extractability Relevance of
- ☐ recommendation

Indicator / recommendation	Source	Year	Level of evidence
**RECOMMENDATION:**<br>**It is advisable that communication limitations are recorded in the EPD so that the care provider can take them into account.**<br>**ICPC-2 codes:**<br>• F94 blind/visual impairment (all forms)<br>• H84 Presbyacusis<br>• H86 Deafness<br>• In range 28 (Limited function/disability)	NHS	2023	No gradation

Your assessment:

*

Choose one of the following options:

- ◯ 1 (Poor)
- ◯ 2
- ◯ 3
- ◯ 4
- ◯ 5
- ◯ 6
- ◯ 7
- ◯ 8
- ◯ 9 (Excellent)
- ◯ Not assessable

Assessment based on:

Select all options that apply to you:

- ☐ EMD extractability Relevance of
- ☐ recommendation

Indicator / recommendation	Source	Year	Level of evidence
**RECOMMENDATION:** **In the EPD, additional information regarding the diagnosis is best recorded in a caption or comment that provides more information about the aspects of the condition.**	**HIQA**	**2018**	**No evidence**

Your opinion:

*

Select one of the following options:

- ◯ 1 (Poor)
- ◯ 2
- ◯ 3
- ◯ 4
- ◯ 5
- ◯ 6
- ◯ 7
- ◯ 8
- ◯ 9 (Excellent)
- ◯ Not assessable

Assessment based on:

Select all options that apply to you:

- ☐ EMD extractability Relevance of
- ☐ recommendation

Indicator / recommendation	Source	Year	Level of evidence
**RECOMMENDATION:** The start date of each care element must be recorded in the EPD.	HIQA	2018	No grading

Your opinion:

*

Select one of the following options:

- ◯ 1 (Poor)
- ◯ 2
- ◯ 3
- ◯ 4
- ◯ 5
- ◯ 6
- ◯ 7
- ◯ 8
- ◯ 9 (Excellent)
- ◯ Not assessable

Assessment based on:

Select all options that apply to you:

- ☐ EMD extractability Relevance of
- ☐ recommendation

Indicator / recommendation	Source	Year	Level of evidence
**RECOMMENDATION:** The end date or presumed end date of each completed care element must be recorded in the EPD.	HIQA	2018	No grading

Your opinion:

*

Select one of the following options:

- ◯ 1 (Poor)
- ◯ 2
- ◯ 3
- ◯ 4
- ◯ 5
- ◯ 6
- ◯ 7
- ◯ 8
- ◯ 9 (Excellent)
- ◯ Not assessable

Assessment based on:

Select all options that apply to you:

- ☐ EMD extractability Relevance of
- ☐ recommendation

Indicator / Recommendation	Source	Year	Level of evidence
**RECOMMENDATION:** **If the patient has no known illnesses, this should be recorded in the EPD. This ensures that other healthcare providers know that the file is complete. (ICPC-2: A97)**	**HIQA**	**2018**	**/**

Your opinion:

*

Choose one of the following options:

- ◯ 1 (Poor)
- ◯ 2
- ◯ 3
- ◯ 4
- ◯ 5
- ◯ 6
- ◯ 7
- ◯ 8
- ◯ 9 (Excellent)
- ◯ Not assessable

Assessment based on:

Select all options that apply to you:

- ☐ EMD extractability Relevance of
- ☐ recommendation

Recommendation	Source	Year	Level of evidence
**RECOMMENDATION:**<br>**The EPD contains: all conditions or data that are relevant to further care, such as certain procedures and conditions that may recur.**<br>**Clarification: this refers to operations and major treatments as described in the NHG Intervention Viewer**	**ADEPD, Domus Medica, HIQA, NCQA**	**2019, 2004, 2020, 2018**	**No rating**

Your rating:

*

Choose one of the following options:

- ◯ 1 (Poor)
- ◯ 2
- ◯ 3
- ◯ 4
- ◯ 5
- ◯ 6
- ◯ 7
- ◯ 8
- ◯ 9 (Excellent)
- ◯ Not assessable

Assessment based on:

Select all options that apply to you:

- ☐ EMD extractability Relevance of
- ☐ recommendation

Indicator / recommendation	Source	Year	Level of evidence
**RECOMMENDATION:** Preferably record operations and important treatments in the relevant episode. Do this using the functionality of your HIS. This will also ensure that this information is included in the Procedures and Treatments overview.	ADEPD	2019	/

Your opinion:

*

Choose one of the following options:

- ◯ 1 (Poor)
- ◯ 2
- ◯ 3
- ◯ 4
- ◯ 5
- ◯ 6
- ◯ 7
- ◯ 8
- ◯ 9 (Excellent)
- ◯ Not assessable

Assessment based on:

Select all options that apply to you:

- ☐ EMD extractability Relevance of
- ☐ recommendation

Indicator / recommendation	Source	Year	Level of evidence
**RECOMMENDATION:** The EPD contains a caption with information about the procedure for relevant operations and interventions.	HIQA	2018	No grading

Your opinion:

*

Select one of the following options:

- ◯ 1 (Poor)
- ◯ 2
- ◯ 3
- ◯ 4
- ◯ 5
- ◯ 6
- ◯ 7
- ◯ 8
- ◯ 9 (Excellent)
- ◯ Not assessable

Assessment based on:

Select all options that apply to you:

- ☐ EMD extractability Relevance of
- ☐ recommendation

Indicator / recommendation	Source	Year	Level of evidence
**RECOMMENDATION:** The EPD contains the date on which relevant operations and procedures were performed.	HIQA	2018	No grading

Your opinion:

*

Select one of the following options:

- ◯ 1 (Poor)
- ◯ 2
- ◯ 3
- ◯ 4
- ◯ 5
- ◯ 6
- ◯ 7
- ◯ 8
- ◯ 9 (Excellent)
- ◯ Not assessable

Assessment based on:

Select all options that apply to you:

- ☐ EMD extractability Relevance of
- ☐ recommendation

Indicator / recommendation	Source	Year	Level of evidence
**RECOMMENDATION:** The EPD contains a record if the patient has not had any operations or procedures in the past.	HIQA	2018	Adjust the gradation

Your opinion:

*

Select one of the following options:

- ◯ 1 (Poor)
- ◯ 2
- ◯ 3
- ◯ 4
- ◯ 5
- ◯ 6
- ◯ 7
- ◯ 8
- ◯ 9 (Excellent)
- ◯ Not assessable

Assessment based on:

Select all options that apply to you:

- ☐ EMD extractability Relevance of
- ☐ recommendation

Top 5 recommendations:

Which recommendations for the "completeness and adequacy of the problem list" do you consider most suitable for measuring the quality of good use of the EPD in general practice?

1.

*

Choose one of the following options:

- How many active care elements does a patient have on average on his/her list of care elements?
- What percentage of episodes worthy of problem status are indeed labelled 'special attention'?
- A number of medications are prescribed specifically for a particular disease. For patients with a prescription for this medication, a care element with that diagnosis must be present.
- Percentage of registered patients for whom there has been no change in the EPD over the past 12 months.
- Percentage of the patient population, aged 18 years and older, with chronic health problems who have received at least one of the following types of self-management support from their primary care provider: Provided with a treatment plan; Encouraged to use self-help groups or programmes
- What percentage of episodes on the episode list have a valid ICPC code?
- It is recommended that communication limitations be recorded in the EPD so that the healthcare provider can take them into account.
- In the EPD, additional information regarding the diagnosis is best recorded in a caption or comment that provides more information about the aspects of the condition.
- The start date of each care element must be recorded in the EPD.
- The end date or presumed end date of each completed care element must be recorded in the EPD.
- If the patient has no known illnesses, this should be recorded in the EPD.
- The EPD contains: operations and procedures that are important for further care, such as certain procedures and conditions that may recur.
- Preferably record operations and important treatments under the relevant episode.
- The EPD contains a caption with information about the procedure for relevant operations and procedures. The
- EPD contains the date on which relevant operations and procedures were performed.
- The EPD contains a record if the patient has not had any operations or procedures in the past.

2.

*

Choose one of the following options:

- ◯ How many active care elements does a patient have on average on his/her list of care elements?
- ◯ What percentage of episodes worthy of problem status are indeed labelled 'special attention'?
- ◯ A number of medications are prescribed specifically for a particular disease. For patients with a prescription for this medication, a care element with that diagnosis must be present.
- ◯ Percentage of registered patients for whom there has been no change in the EPD over the past 12 months.
- ◯ Percentage of the patient population, aged 18 years and older, with chronic health problems who have received at least one of the following types of self-management support from their primary care provider: Provided with a treatment plan; Encouraged to use self-help groups or programmes
- ◯ What percentage of episodes on the episode list have a valid ICPC code?
- ◯ It is recommended that communication limitations be recorded in the EPD so that the healthcare provider can take them into account.
- ◯ In the EPD, additional information regarding the diagnosis is best recorded in a caption or comment that provides more information about the aspects of the condition.
- ◯ The start date of each care element must be recorded in the EPD.
- ◯ The end date or presumed end date of each completed care element must be recorded in the EPD.
- ◯ If the patient has no known illnesses, this should be recorded in the EPD.
- ◯ The EPD contains: operations and procedures that are important for further care, such as certain procedures and conditions that may recur.
- ◯ Preferably record operations and important treatments under the relevant episode.
- ◯ The EPD contains a caption with information about the procedure for relevant operations and procedures. The
- ◯ EPD contains the date on which relevant operations and procedures were performed.
- ◯ The EPD contains a record if the patient has not had any operations or procedures in the past.
- ◯
- ◯

3.

*

Choose one of the following options:

- ◯ How many active care elements does a patient have on average on his/her list of care elements?
- ◯ What percentage of episodes worthy of problem status are indeed labelled 'special attention'?
- ◯ A number of medications are prescribed specifically for a particular disease. For patients with a prescription for this medication, a care element with that diagnosis must be present.
- ◯ Percentage of registered patients for whom there has been no change in the EPD over the past 12 months.
- ◯ Percentage of the patient population, aged 18 years and older, with chronic health problems who have received at least one of the following types of self-management support from their primary care provider: Provided with a treatment plan; Encouraged to use self-help groups or programmes
- ◯ What percentage of episodes on the episode list have a valid ICPC code?
- ◯ It is recommended that communication limitations be recorded in the EPD so that the healthcare provider can take them into account.
- ◯ In the EPD, additional information regarding the diagnosis is best recorded in a caption or comment that provides more information about the aspects of the condition.
- ◯ The start date of each care element must be recorded in the EPD.
- ◯ The end date or presumed end date of each completed care element must be recorded in the EPD.
- ◯ If the patient has no known illnesses, this should be recorded in the EPD.
- ◯ The EPD contains: operations and procedures that are important for further care, such as certain procedures and conditions that may recur.
- ◯ Preferably record operations and important treatments under the relevant episode.
- ◯ The EPD contains a caption with information about the procedure for relevant operations and procedures. The
- ◯ EPD contains the date on which relevant operations and procedures were performed.
- ◯ The EPD contains a record if the patient has not had any operations or procedures in the past.

4.

*

Choose one of the following options:

- ◯ How many active care elements does a patient have on average on his/her list of care elements?
- ◯ What percentage of episodes worthy of problem status are indeed labelled 'special attention'?
- ◯ A number of medications are prescribed specifically for a particular disease. For patients with a prescription for this medication, a care element with that diagnosis must be present.
- ◯ Percentage of registered patients for whom there has been no change in the EPD over the past 12 months.
- ◯ Percentage of the patient population, aged 18 years and older, with chronic health problems who have received at least one of the following types of self-management support from their primary care provider: Provided with a treatment plan; Encouraged to use self-help groups or programmes
- ◯ What percentage of episodes on the episode list have a valid ICPC code?
- ◯ It is recommended that communication limitations be recorded in the EPD so that the healthcare provider can take them into account.
- ◯ In the EPD, additional information regarding the diagnosis is best recorded in a caption or comment that provides more information about the aspects of the condition.
- ◯ The start date of each care element must be recorded in the EPD.
- ◯ The end date or presumed end date of each completed care element must be recorded in the EPD.
- ◯ If the patient has no known illnesses, this should be recorded in the EPD.
- ◯ The EPD contains: operations and procedures that are important for further care, such as certain procedures and conditions that may recur.
- ◯ Preferably record operations and important treatments under the relevant episode.
- ◯ The EPD contains a caption with information about the procedure for relevant operations and procedures. The
- ◯ EPD contains the date on which relevant operations and procedures were performed.
- ◯ The EPD contains a record if the patient has not had any operations or procedures in the past.
- ◯
- ◯

5.

*****

Choose one of the following options:

- ◯ How many active care elements does a patient have on average on his/her list of care elements?
- ◯ What percentage of episodes worthy of problem status are indeed labelled 'special attention'?
- ◯ A number of medications are prescribed specifically for a particular disease. For patients with a prescription for this medication, a care element with that diagnosis must be present.
- ◯ Percentage of registered patients for whom there has been no change in the EPD over the past 12 months.
- ◯ Percentage of the patient population, aged 18 years and older, with chronic health problems who have received at least one of the following types of self-management support from their primary care provider: Provided with a treatment plan; Encouraged to use self-help groups or programmes
- ◯ What percentage of episodes on the episode list have a valid ICPC code?
- ◯ It is recommended that communication limitations be recorded in the EPD so that the healthcare provider can take them into account.
- ◯ In the EPD, additional information regarding the diagnosis is best recorded in a caption or comment that provides more information about the aspects of the condition.
- ◯ The start date of each care element must be recorded in the EPD.
- ◯ The end date or presumed end date of each completed care element must be recorded in the EPD.
- ◯ If the patient has no known illnesses, this should be recorded in the EPD.
- ◯ The EPD contains: operations and procedures that are important for further care, such as certain procedures and conditions that may recur.
- ◯ Preferably record operations and important treatments under the relevant episode.
- ◯ The EPD contains a caption with information about the procedure for relevant operations and procedures. The
- ◯ EPD contains the date on which relevant operations and procedures were performed.
- ◯ The EPD contains a record if the patient has not had any operations or procedures in the past.

If you have any suggestions for recommendations that you believe are missing from the above list, please note them in the box below. Please always clearly state a recommendation and the reason for this recommendation.

Enter your answer here:

2. Structured registration in EPD

To what extent are the following recommendations relevant for measuring the quality of the proper use of the EMD in general practice in relation to **structured registration in the EPD**?

Indicator / recommendation	Source	Year	Level of evidence
**INDICATOR:**<br><br>**What percentage of the sub-contacts in the journal are linked to care elements with a valid ICPC code?**<br><br>**ICPC code further divided into:**<br><br>◆ **Correct use: Complaints in the range 01 to 29 and diagnoses in the range 70 to 99. This category also includes A44 (vaccination), R44 (influenza vaccination) and X37 (cervical smear screening). Possible incorrect use: A97 (no disease) or A99 (other generalised or unspecified disease) (as flight code);**<br>◆ **Unauthorised or no ICPC: No ICPC or an unauthorised code (range 30-69), with the exception of A44 (preventive vaccination or medication), R44 (influenza vaccination) and X37 (cervical smear screening).**	**EPD scan-h**	**2009**	**No grading**

Your assessment:

*

Choose one of the following options:

- 1 (Poor)
- 2
- 3
- 4
- 5
- 6
- 7
- 8
- 9 (Excellent)
- Not assessable

Assessment based on:

Select all options that apply to you:

- [ ] EMD extractability Relevance of
- [ ] recommendation

Indicator / recommendation	Source	Year	Level of evidence
**INDICATOR:** **What percentage of the sub-contacts recorded during consultations, telephone contacts and home visits have a Subjective, Objective, Evaluation or Planning rule?**	**EPD scan-h**	**2009**	**No gradation**
**RELATED RECOMMENDATION:** **Partial contact report: the EPD presents SOEP rules from the partial contact from which the GP refers. If necessary, the GP selects additional sub-contacts from the episode.**	**HASP**	**2017**	**No gradation**

Your assessment:

*

Choose one of the following options:

- ◯ 1 (Poor)
- ◯ 2
- ◯ 3
- ◯ 4
- ◯ 5
- ◯ 6
- ◯ 7
- ◯ 8
- ◯ 9 (Excellent)
- ◯ Not assessable

Assessment based on:

Select all options that apply to you:

☐ EMD extractability Relevance of
☐ recommendation

Indicator / Recommendation	Source	Year	Level of evidence
**INDICATOR:** **Number of consultations for online sources (e.g. CDLH)**	**Hamade et al.**	**2008**	**No gradation**

Your assessment:

*

Choose one of the following options:

- ◯ 1 (Poor)
- ◯ 2
- ◯ 3
- ◯ 4
- ◯ 5
- ◯ 6
- ◯ 7
- ◯ 8
- ◯ 9 (Excellent)
- ◯ Not assessable

Assessment based on:

Select all options that apply to you:

☐ EMD extractability Relevance of
☐ recommendation

Indicator / recommendation	Source	Year	Level of evidence
**INDICATOR:**<br>Number of changes in schedules per week per 1,000 GMD patients	Hamade et al.	2008	No gradation
**RELATED RECOMMENDATION:**<br>Encounter forms or notes have a notation, regarding follow-up care, calls or visits, when indicated. The specific time of return is noted in weeks, months or as needed.	NCQA	2018	No grading

Your opinion:

*

Choose one of the following options:

- ◯ 1 (Poor)
- ◯ 2
- ◯ 3
- ◯ 4
- ◯ 5
- ◯ 6
- ◯ 7
- ◯ 8
- ◯ 9 (Excellent)
- ◯ Not assessable

Assessment based on:

Select all options that apply to you:

- ☐ EMD extractability Relevance of
- ☐ recommendation

Indicator / recommendation	Source	Year	Level of evidence
**RECOMMENDATION:** Consultation, laboratory and imaging reports filed in the chart are initialed by the practitioner who ordered them, to signify review. (Review and signature by professionals other than the ordering practitioner do not meet this requirement.) If the reports are presented electronically or by some other method, there is also representation of review by the ordering practitioner. Consultation and abnormal laboratory and imaging study results have an explicit notation in the record of follow-up plans.	NCQA	2018	No grading

Your assessment:

*

Choose one of the following options:

- ◯ 1 (Poor)
- ◯ 2
- ◯ 3
- ◯ 4
- ◯ 5
- ◯ 6
- ◯ 7
- ◯ 8
- ◯ 9 (Excellent)
- ◯ Not assessable

Assessment based on:

Select all options that apply to you:

☐ EMD extractability Relevance of
☐ recommendation

Top 5 recommendations:

Which recommendations for the "completeness and timeliness of medication overview" do you consider most suitable for measuring the quality of proper use of the EPD in general practice?

1.

*

Choose one of the following options:

○ What percentage of the partial contacts in the journal are linked to care elements with a valid ICPC code?
○ What percentage of the partial contacts recorded during consultations, telephone contacts and home visits have a Subjective, Objective, Evaluation or Planning rule?
○ Number of consultations for online sources (e.g. CDLH)
○ Number of changes to schedules per week per 1,000 GMD patients
○ Consultation, laboratory and imaging reports filed in the chart are initialed by the practitioner who ordered them, to signify review

2.

*

Select one of the following options:

○ What percentage of the partial contacts in the journal are linked to care elements with a valid ICPC code?
○ What percentage of the sub-contacts recorded during consultations, telephone contacts and home visits have a Subjective, Objective, Evaluation or Planning rule?
○ Number of consultations for online sources (e.g. CDLH)
○ Number of changes to schedules per week per 1,000 GMD patients
○ Consultation, laboratory and imaging reports filed in the chart are initialed by the practitioner who ordered them, to signify review

3.

*

Select one of the following options:

◯ What percentage of the partial contacts in the journal are linked to care elements with a valid ICPC code?

◯ What percentage of the sub-contacts recorded during consultations, telephone contacts and home visits have a Subjective, Objective, Evaluation or Planning rule?

◯ Number of consultations for online sources (e.g. CDLH)

◯ Number of changes to schedules per week per 1,000 GMD patients

◯ Consultation, laboratory and imaging reports filed in the chart are initialed by the practitioner who ordered them, to signify review

If you have any suggestions for recommendations that you believe are missing from the above list, please note them in the box below. Please always clearly state a recommendation and the reason for this recommendation.

Enter your answer here:

3. Completeness and timeliness of medication overview

To what extent are the following recommendations relevant for measuring the quality of the proper use of the EMD in general practice in relation to the **completeness and timeliness of** patients' **medication overviews**?

Indicator / Recommendation	Source	Year	Level of evidence
**INDICATOR:**<br>What percentage of the medication on the 'current medication' list is incorrectly labelled as current medication?<br>The professional summary should include an overview of current medication (i.e. medication that the patient is currently taking, either temporarily or chronically (> 6 months)) and medication that has been discontinued in recent months (4-6 months depending on the source). As a rule, a general practitioner prescribes medication for three months, with exceptions up to six months for the contraceptive pill, for example.<br>Medication that has not been prescribed for more than 6 months is probably no longer current.	EPD-scan-h	2009	No gradation
**RELATED RECOMMENDATION:**<br>The EPD presents the current prescriptions. The HIS allows you to add previous medication to the episode from which the referral is made, including the reason for stopping and/or self-medication.	ADEPD, Domus Medica, SSMG, NHS, HIQA	2019, 2004, ?, 2023, 2020	No grading

Your rating:

*

Select one of the following options: 1 (Poor)

- ◯ 2
- ◯ 3
- ◯ 4
- ◯ 5
- ◯ 6
- ◯ 7
- ◯ 8
- ◯ 9 (Excellent)
- ◯ Not assessable
- ◯

Assessment based on:

Select all options that apply to you:

- ☐ EMD extractability Relevance of
- ☐ recommendation

Indicator / Recommendation	Source	Year	Level of evidence
**INDICATOR:**<br>What percentage of prescriptions are linked to an episode?<br>For this indicator, we selected the current medication and the medication that has been discontinued in the past four months.<br>ICPC code to be further divided into:<br>• Correct usage: Complaints in the range 01 to 29 and diagnoses in the range 70 to 99. This category also includes A44 (vaccination), R44 (influenza vaccination)<br>• and X37 (cervical smear screening). Possible incorrect use: A97 (no disease) or A99 (other generalised or unspecified disease) (as flight code);<br>• Unauthorised or no ICPC: No ICPC or an unauthorised code (range 30-69), with the exception of A44 (preventive vaccination or medication), R44 (influenza vaccination) and X37 (cervical smear screening).	EPD scan-h	2009	No grading

Your assessment:

*

Choose one of the following options: 1

- ◯ (Poor)
- ◯ 2
- ◯ 3
- ◯ 4
- ◯ 5
- ◯ 6
- ◯ 7
- ◯ 8
- ◯ 9 (Excellent)
- ◯ Not assessable

Assessment based on:

Select all options that apply to you:

- ☐ EMD extractability Relevance of
- ☐ recommendation

Indicator / Recommendation	Source	Year	Level of evidence
**INDICATOR:**<br><br>**Full dosage and treatment schedule with regard to dose-effect or side effects of medication.**	**De Lusignan et al**	**2002**	**No grading**
**RELATED RECOMMENDATION:**<br><br>• **The dosage for each prescribed medication must be recorded in the EPD.**<br>• **For each prescribed medication, the time of administration must be recorded in the EPD.**<br>• **For each prescribed medication, the duration of administration must be recorded in the EPD.**<br>• **For each prescribed medication, the method of administration must be recorded in the EPD.**<br>• **For each prescribed medication, the start date must be recorded in the EPD.**	**HIQA**	**2018**	**No grading**

Your opinion:

*

Choose one of the following options:

◯ 1 (Poor)

◯ 2

- 3
- 4
- 5
- 6
- 7
- 8
- 9 (Excellent)
- Not assessable

Assessment based on:

Select all options that apply to you:

- [ ] EMD extractability Relevance of
- [ ] recommendation

Indicator / recommendation	Source	Year	Level of evidence
**INDICATOR:** **Number of prescriptions for medication per week per 1,000 GMD patients?** **This is a rough measure of how many prescriptions are not issued digitally. It is also a detection tool for missing data over time.**	**De Lusignan.**	**2002**	**No gradation**

Your assessment:

*

Choose one of the following options:

- ◯ 1 (Poor)
- ◯ 2
- ◯ 3
- ◯ 4
- ◯ 5
- ◯ 6
- ◯ 7
- ◯ 8
- ◯ 9 (Excellent)
- ◯ Not assessable

Assessment based on:

Select all options that apply to you:

- ☐ EMD extractability
- ☐ Relevance of recommendation

Indicator / Recommendation	Source	Year	Level of evidence
**RECOMMENDATION:** If the patient is not taking any medication, this should be correctly noted in the EPD.	HIQA	2018	No grading

Your opinion:

*

Select one of the following options:

- ◯ 1 (Poor)
- ◯ 2
- ◯ 3
- ◯ 4
- ◯ 5
- ◯ 6
- ◯ 7
- ◯ 8
- ◯ 9 (Excellent)
- ◯ Not assessable

Assessment based on:

Select all options that apply to you:

- ☐ EMD extractability Relevance of
- ☐ recommendation

Top 3 recommendations:

Which recommendations for the "completeness and timeliness of medication overview" do you consider most suitable for measuring the quality of proper use of the EPD in general practice?

1.

*

Choose one of the following options:

- What percentage of the medication on the 'current medication' list is incorrectly labelled as current medication? What percentage of prescriptions are linked to an episode?
- Complete dosage and treatment schedule with regard to dose-effect or side effects of medication. If the patient is not taking medication, this must be correctly noted in the EPD.
- Number of prescriptions for medication per week per 1,000 GMD patients?

2.

*

Choose one of the following options:

- What percentage of the medication on the 'current medication' list is incorrectly labelled as current medication? What percentage of prescriptions are linked to an episode?
- Full dosage and treatment schedule with regard to dose-effect or side effects of medication. If the patient is not taking medication, this must be correctly noted in the EPD.
- Number of prescriptions for medication per week per 1,000 GMD patients?

3.

*

Choose one of the following options:

- What percentage of the medication on the 'current medication' list is incorrectly labelled as current medication? What percentage of prescriptions are linked to an episode?
- Complete dosage and treatment schedule with regard to dose-effect or side effects of medication. If the patient is not taking medication, this must be correctly noted in the EPD.
- Number of prescriptions for medication per week per 1,000 GMD patients?

If you have any suggestions for recommendations that you believe are missing from the above list, please note them in the box below. Please clearly state your recommendation and the reason for it.

Enter your answer here:

4. Risk factors / Medication monitoring

To what extent are the following recommendations relevant for measuring the quality of the proper use of the EMD in general practice in relation to **risk factors and medication monitoring of patients**?

Indicator / recommendation	Source	Year	Level of evidence
**INDICATOR:** **Number of new appointments for colorectal cancer or breast cancer screening per week per 1,000 GMD patients**	**Hamade et al.**	**2008**	**No level of evidence**

Your assessment:

*

Choose one of the following options:

- 1 (Poor)
- 2
- 3
- 4
- 5
- 6
- 7
- 8
- 9 (Excellent)
- Not assessable

Assessment based on:

Select all options that apply to you:

- [ ] EMD extractability Relevance of
- [ ] recommendation

Indicator / Recommendation	Source	Year	Level of evidence
**INDICATOR:**<br>**How many patients have been registered with a contraindication to medication?**<br>**Contraindications are conditions that must be taken into account when prescribing medication. Examples include diabetes mellitus or renal insufficiency. They can also be temporary contraindications, such as during pregnancy.**	**EPD scan-h**	**2009**	**No level of evidence**
**LINKED RECOMMENDATION:**<br>**Hypersensitivity to medication, contraindications for prescription. The EPD presents medication hypersensitivity and contraindications respectively.**	**ADEPD**	**2019**	**No gradation**

Your assessment:

*

Choose one of the following options:

- ◯ 1 (Poor)
- ◯ 2
- ◯ 3
- ◯ 4
- ◯ 5
- ◯ 6
- ◯ 7
- ◯ 8

◯ 9 (Excellent)

◯ Not assessable

Assessment based on:

Select all options that apply to you:

☐ EMD extractability Relevance of

☐ recommendation

Indicator / Recommendation	Source	Year	Level of evidence
**INDICATOR:**<br>**How many patients have a recorded drug allergy or intolerance?**	**EPD scan-h**	**2008**	**No level of evidence**
**RELATED RECOMMENDATION:**<br>**The patient summary SHALL identify the substance to which the patient has a susceptibility to an allergy upon exposure. It includes allergies, intolerances and adverse reactions to all substances, not only those arising from medications or medicines. It also describes other clinical information that is imperative to know so that the life or health of the patient does not come under threat. For example, intolerance to aspirin due to gastrointestinal bleeding.**<br>**The patient summary SHOULD describe the type of reaction event as determined by the healthcare practitioner.**<br>**The patient summary SHOULD include the severity of the symptom as determined by the healthcare practitioner.**<br>**The patient summary SHOULD contain a record**	**HIQA**	**2020**	**No grading**

Indicator / Recommendation	Source	Year	Level of evidence
**of the date and/or time of the onset of the reaction.**			

Your opinion:

*

Choose one of the following options: 1

- ◯ (Poor)
- ◯ 2
- ◯ 3
- ◯ 4
- ◯ 5
- ◯ 6
- ◯ 7
- ◯ 8
- ◯ 9 (Excellent)
- ◯ Not assessable

Assessment based on:

Select all options that apply to you:

- ☐ EMD extractability Relevance of
- ☐ recommendation

Indicator / Recommendation	Source	Year	Level of evidence
**RECOMMENDATION:** **Certain medical information is important for the entire care chain. The NHG recommends registration of: prophylaxis for addison crisis, endocarditis, bleeding disorders, endoprosthesis, immunocompromised patient, (functional) asplenia, thrombosis or particularly resistant microorganisms.**	**ADEPD**	**2019**	**No grading**

Your opinion:

*

Choose one of the following options:

- ◯ 1 (Poor)
- ◯ 2
- ◯ 3
- ◯ 4
- ◯ 5
- ◯ 6
- ◯ 7
- ◯ 8
- ◯ 9 (Excellent)
- ◯ Not assessable

Assessment based on:

Select all options that apply to you:

- ☐ EMD extractability
- ☐ Relevance of recommendation

Indicator / recommendation	Source	Year	Level of evidence
**RECOMMENDATION:**<br><br>**Physical examinations can be recorded in free text or as diagnostic tests in the EPD. If recorded as a diagnostic test, it can be reused for overviews over time and for decision support. Where possible, physical examinations should therefore be recorded as diagnostic tests in the EPD as much as possible.**<br><br>**Diagnostic determinations include, for example: weight, height, blood pressure, heart rhythm, alcohol and nicotine use, substance use, amount of physical activity, etc.**<br><br>**A complete list can be found at: NHG - Test Viewer**	**ADEPD**	**2019**	**No gradation**

Your assessment:

*

Choose one of the following options:

- ◯ 1 (Poor)
- ◯ 2
- ◯ 3
- ◯ 4
- ◯ 5
- ◯ 6
- ◯ 7
- ◯ 8
- ◯ 9 (Excellent)

Not assessable

Assessment based on:

Select all options that apply to you:

- [ ] EMD extractability Relevance of
- [ ] recommendation

Indicator / recommendation	Source	Year	Level of evidence
**RECOMMENDATION:** **For all patients aged 12 years and older, a note should be made in the EMD regarding the use of cigarettes, alcohol and drugs.**	**NCQA, HASP, NHS**	**2018, 2017, 2023**	**No level of evidence**

Your opinion:

*

Choose one of the following options:

- 1 (Poor)
- 2
- 3
- 4
- 5
- 6
- 7
- 8
- 9 (Excellent)
- Not assessable

Assessment based on:

Select all options that apply to you:

- [ ] EMD extractability Relevance of
- [ ] recommendation

Indicator / Recommendation	Source	Year	Level of evidence
**RECOMMENDATION:** **If the family medical history section is available in the EPD, record information about conditions that occur in blood relatives, specifying for each condition which family members are affected, at what age it started and, if applicable, at what age they died from it. The source of this information is often the patient themselves. Leave the fields blank if the information is unclear or unknown.**	ADEPH	2019	No level of evidence

Your assessment:

*

Choose one of the following options:

- ◯ 1 (Poor)
- ◯ 2
- ◯ 3
- ◯ 4
- ◯ 5
- ◯ 6
- ◯ 7
- ◯ 8
- ◯ 9 (Excellent)
- ◯ Not assessable

Assessment based on:

Select all options that apply to you:

☐ EMD extractability Relevance of
☐ recommendation

Indicator / Recommendation	Source	Year	Level of evidence
**RECOMMENDATION:** **For patients with no known drug allergies or intolerances, this should be noted in the EPD.**	**NCQA HIQA**	**2018 2020**	**No level of evidence**

Your assessment:

*

Choose one of the following options:

◯ 1 (Poor)
◯ 2
◯ 3
◯ 4
◯ 5
◯ 6
◯ 7
◯ 8
◯ 9 (Excellent)
◯ Not assessable

Assessment based on:

Select all options that apply to you:

☐ EMD extractability Relevance of
☐ recommendation

Indicator / Recommendation	Source	Year	Level of evidence
**RECOMMENDATION:** **Psychogeriatric examination: if a vulnerable person is selected, this is noted in the EPD in the agreed sections on vulnerability.**	**HASP**	**2017**	**No level of evidence**

Your opinion:

*

Select one of the following options:

- ◯ 1 (Poor)
- ◯ 2
- ◯ 3
- ◯ 4
- ◯ 5
- ◯ 6
- ◯ 7
- ◯ 8
- ◯ 9 (Excellent)
- ◯ Not assessable

Assessment based on:

Select all options that apply to you:

- ☐ EMD extractability Relevance of
- ☐ recommendation

Indicator / Recommendation	Source	Year	Level of evidence
**RECOMMENDATION:** **Social data is recorded in the Social Data section of the file. Social data includes: education, occupation, work and socio-economic status. Language and language skills, health skills, living situation, family situation, presence of informal care, migration history, beliefs and intense emotional events in the past also fall under social data.**	**ADEPD**	**2019**	**No level of evidence**

Your assessment:

*

Choose one of the following options:

- ◯ 1 (Poor)
- ◯ 2
- ◯ 3
- ◯ 4
- ◯ 5
- ◯ 6
- ◯ 7
- ◯ 8
- ◯ 9 (Excellent)
- ◯ Not assessable

Assessment based on:

Select all options that apply to you:

- [ ] EMD extractability Relevance of
- [ ] recommendation

Top 5 recommendations:

Which recommendations for "risk factors/medication monitoring" do you consider most suitable for measuring the quality of proper use of the EPD in general practice?

1.

*

Choose one of the following options:

- Number of new appointments for colon cancer or breast cancer screening per week per 1,000 GMD patients For how many
- patients is a medication contraindication registered?
- How many patients have a registered drug allergy or intolerance?
- The NHG recommends recording: prophylaxis for Addison's crisis, endocarditis, bleeding disorders, endoprosthesis, immunocompromised patients, (functional) asplenia, thrombosis or particularly resistant microorganisms.
- Where possible, the physical examination should therefore be recorded as a diagnostic determination in the EPD as much as possible.
- For all patients aged 12 and older, a note should be made in the EMD regarding the use of cigarettes, alcohol and drugs.
- If the EPD contains a section on family history, record information about conditions that occur in blood relatives.
- For patients with no known drug allergies or intolerances, this should be noted in the EPD.
- Psychogeriatric examination: if a vulnerable person is selected, this is noted in the EPD in the agreed sections on vulnerability.
- Social data is recorded in the Social Data section of the file.

2.

*

Select one of the following options:

- Number of new appointments for colon cancer or breast cancer screening per week per 1,000 GMD patients For how many
- patients is a medication contraindication recorded?
- How many patients have a registered drug allergy or intolerance?
- The NHG recommends recording: prophylaxis for Addison's crisis, endocarditis, bleeding disorders, endoprosthesis, immunocompromised patients, (functional) asplenia, thrombosis or particularly resistant microorganisms.
- Where possible, the physical examination should therefore be recorded as a diagnostic determination in the EPD as much as possible.
- For all patients aged 12 years and older, a note should be made in the EMD regarding the use of cigarettes, alcohol and drugs.
- If the EPD contains a section on family medical history, record information about conditions that occur in blood relatives.
- For patients with no known drug allergies or intolerances, this should be noted in the EPD.
- Psychogeriatric examination: if a vulnerable person is selected, this is noted in the EPD in the agreed sections on vulnerability.
- Social data is recorded in the Social Data section of the file.

3.

*

Select one of the following options:

- Number of new appointments for colon cancer or breast cancer screening per week per 1,000 GMD patients How many
- patients have a contraindication to medication recorded?
- How many patients have a registered drug allergy or intolerance?
- The NHG recommends recording: prophylaxis for Addison's crisis, endocarditis, bleeding disorders, endoprosthesis, immunocompromised patients, (functional) asplenia, thrombosis or particularly resistant microorganisms.
- Where possible, the physical examination should therefore be recorded as a diagnostic determination in the EPD as much as possible.
- For all patients aged 12 and older, a note should be made in the EMD regarding the use of cigarettes, alcohol and drugs.
- If the EPD contains a section on family history, record information about conditions that occur in blood relatives.
- For patients with no known drug allergies or intolerances, this should be noted in the EPD.
- Psychogeriatric examination: if a vulnerable person is selected, this is noted in the EPD in the agreed sections on vulnerability.
- Social data is recorded in the Social Data section of the file.

4.

*

Select one of the following options:

- Number of new appointments for colon cancer or breast cancer screening per week per 1,000 GMD patients For how many
- patients is a medication contraindication recorded?
- How many patients have a registered drug allergy or intolerance?
- The NHG recommends recording: prophylaxis for Addison's crisis, endocarditis, bleeding disorders, endoprosthesis, immunocompromised patients, (functional) asplenia, thrombosis or particularly resistant microorganisms.
- Where possible, the physical examination should therefore be recorded as a diagnostic determination in the EPD as much as possible.
- For all patients aged 12 and older, a note should be made in the EMD regarding the use of cigarettes, alcohol and drugs.
- If the EPD contains a section on family history, record information about conditions that occur in blood relatives.
- For patients with no known drug allergies or intolerances, this should be noted in the EPD.
- Psychogeriatric examination: if a vulnerable person is selected, this is noted in the EPD in the agreed sections on vulnerability.
- Social data is recorded in the Social Data section of the file.

5.

*

Select one of the following options:

- Number of new appointments for colon cancer or breast cancer screening per week per 1,000 GMD patients For how many
- patients is a medication contraindication recorded?
- How many patients have a registered drug allergy or intolerance?
- The NHG recommends recording: prophylaxis for Addison's crisis, endocarditis, bleeding disorders, endoprosthesis, immunocompromised patients, (functional) asplenia, thrombosis or particularly resistant microorganisms.
- Where possible, the physical examination should therefore be recorded as a diagnostic determination in the EPD as much as possible.
- For all patients aged 12 years and older, a note should be made in the EMD regarding the use of cigarettes, alcohol and drugs.
- If the EPD contains a section on family medical history, record information about conditions that occur in blood relatives.
- For patients with no known drug allergies or intolerances, this should be noted in the EPD.
- Psychogeriatric examination: if the patient is vulnerable, this should be noted in the EPD in the agreed sections on vulnerability.
- Social data is recorded in the Social Data section of the file.

If you have any suggestions for recommendations that you believe are missing from the above list, please note them in the box below. Please always clearly state a recommendation and the reason for this recommendation.

Enter your answer here:

5. Patient identification / Contact information

To what extent are the following recommendations relevant for measuring the quality of the proper use of the EMD in general practice in relation to **patient identification and contact information in the EPD**?

Indicator / recommendation	Source	Year	Level of evidence
**RECOMMENDATION:** **The EPD must record which emergency department (hospital) the patient is known to.**	**NHS**	**2023**	**No gradation**

Your assessment:

*

Choose one of the following options:

- 1 (Poor)
- 2
- 3
- 4
- 5
- 6
- 7
- 8
- 9 (Excellent)
- Not assessable

Assessment based on:

Select all options that apply to you:

- [ ] EMD extractability Relevance of
- [ ] recommendation

Indicator / recommendation	Source	Year	Level of evidence
**RECOMMENDATION:** **The EPD must record whether the patient has been hospitalised.**	NHS	2023	No grading

Your assessment:

*

Choose one of the following options:

- ○ 1 (Poor)
- ○ 2
- ○ 3
- ○ 4
- ○ 5
- ○ 6
- ○ 7
- ○ 8
- ○ 9 (Excellent)
- ○ Not assessable

Assessment based on:

Select all options that apply to you:

- [ ] EMD extractability Relevance of
- [ ] recommendation

Indicator / recommendation	Source	Year	Level of evidence
**RECOMMENDATION:** **The contact details of the contact persons, informal carers and persons designated as representatives are recorded in the file.**	ADEP<br>HIQA<br>SSMG<br>NHS	2019<br>2018<br>?<br>2023	No rating

Your opinion:

*

Select one of the following options:

- ◯ 1 (Poor)
- ◯ 2
- ◯ 3
- ◯ 4
- ◯ 5
- ◯ 6
- ◯ 7
- ◯ 8
- ◯ 9 (Excellent)
- ◯ Not assessable

Assessment based on:

Select all options that apply to you:

- ☐ EMD extractability Relevance of
- ☐ recommendation

Indicator / recommendation	Source	Year	Level of evidence
**RECOMMENDATION:** The EPD should contain information about the care team that monitors the patient.	NHS	2023	No grading

Your opinion:

*

Select one of the following options:

- ◯ 1 (Poor)
- ◯ 2
- ◯ 3
- ◯ 4
- ◯ 5
- ◯ 6
- ◯ 7
- ◯ 8
- ◯ 9 (Excellent)
- ◯ Not assessable

Assessment based on:

Select all options that apply to you:

- ☐ EMD extractability Relevance of
- ☐ recommendation

Indicator / recommendation	Source	Year	Level of evidence
**RECOMMENDATION:** The EPD should contain personal information relevant to the healthcare provider, namely: patient's address, employer, home and work telephone numbers, and marital status.	NCQA	2018	No grading

Your opinion:

*

Select one of the following options:

- ◯ 1 (Poor)
- ◯ 2
- ◯ 3
- ◯ 4
- ◯ 5
- ◯ 6
- ◯ 7
- ◯ 8
- ◯ 9 (Excellent)
- ◯ Not assessable

Assessment based on:

Select all options that apply to you:

- ☐ EMD extractability
- ☐ Relevance of recommendation

Top 3 recommendations:

Which recommendations for "patient identification" do you consider most suitable for measuring the quality of proper use of the EPD in general practice?

1.

*

Choose one of the following options:

- The EPD must record which emergency service (hospital) the patient is known to. The EPD must
- record whether the patient is hospitalised.
- The contact details of the contact persons, informal carers and persons designated as representatives must be recorded in the file.
- The EPD must contain information about the care team that is monitoring the patient.
- The EPD should contain personal information relevant to the healthcare provider, namely: patient's address, employer, home and work telephone numbers, and marital status.

2.

*

Choose one of the following options:

- The EPD must record which emergency service (hospital) the patient is known to. The EPD must
- record whether the patient is hospitalised.
- The contact details of the contact persons, informal carers and persons designated as representatives are recorded in the file.
- The EPD must contain information about the care team that monitors the patient.
- The EPD must contain personal information relevant to the care provider, namely: the patient's address, employer, home and work telephone numbers and marital status.

3.

*

Select one of the following options:

- The EPD must record which emergency service (hospital) the patient is known to. The EPD must
- record whether the patient is hospitalised.
- The contact details of the contact persons, informal carers and persons designated as representatives are recorded in the file.
- The EPD must contain information about the care team that monitors the patient.
- The EPD must contain personal information relevant to the care provider, namely: the patient's address, employer, home and work telephone numbers and marital status.

If you have any suggestions for recommendations that you believe are missing from the above list, please note them in the box below. Please clearly state your recommendation and the reasons for it.

Enter your answer here:

6. Vaccination status

To what extent are the following recommendations relevant for measuring the quality of the proper use of the EMD in general practice in relation to the **vaccination status of patients**?

Indicator / recommendation	Source	Year	Level of evidence
**INDICATOR:** Percentage of the population aged 7 years or older who have received all basic vaccinations. (Overview basic vaccination schedule)	CIHI	2016	No gradation
**RELATED RECOMMENDATION:** A vaccination record (for children) is up to date or an appropriate history is included in the medical record (for adults).	NCQA, NHS, Domus Medica, SSMG, HIQA	2018, 2023, 2004, ?, 2020	No grading

Your rating:

*

Choose one of the following options:

- ◯ 1 (Poor)
- ◯ 2
- ◯ 3
- ◯ 4
- ◯ 5
- ◯ 6
- ◯ 7
- ◯ 8
- ◯ 9 (Excellent)
- ◯ Not assessable

Assessment based on:

Select all options that apply to you:

- ☐ EMD extractability Relevance of
- ☐ recommendation

Indicator / recommendation	Source	Year	Level of evidence
**INDICATOR:**<br>**Percentage of the patient population aged 65 and older who received a flu vaccination.**	**CIHI**	**2016**	**No grading**
**RELATED RECOMMENDATION:**<br>**A vaccination record (for children) is up to date or an appropriate history is included in the medical record (for adults).**	**NCQA, NHS, Domus Medica, SSMG, HIQA**	**2018, 2023, 2004, ?, 2020**	**No grading**

Your rating:

*

Choose one of the following options:

- ◯ 1 (Poor)
- ◯ 2
- ◯ 3
- ◯ 4
- ◯ 5
- ◯ 6
- ◯ 7
- ◯ 8
- ◯ 9 (Excellent)
- ◯ Not assessable

Assessment based on:

Select all options that apply to you:

- ☐ EMD extractability Relevance of
- ☐ recommendation

Indicator / recommendation	Source	Year	Level of evidence
**RECOMMENDATION:** **The EPD should contain information that the patient has not been vaccinated.**	**HIQA**	**2018**	**/**

Your opinion:

*

Select one of the following options:

- ◯ 1 (Poor)
- ◯ 2
- ◯ 3
- ◯ 4
- ◯ 5
- ◯ 6
- ◯ 7
- ◯ 8
- ◯ 9 (Excellent)
- ◯ Not assessable

Assessment based on:

Select all options that apply to you:

- ☐ EMD extractability Relevance of
- ☐ recommendation

Indicator / recommendation	Source	Year	Level of evidence
**RECOMMENDATION:** The EPD should contain the date of administration for each vaccination.	HIQA	2018	/

Your opinion:

*

Select one of the following options:

- 1 (Poor)
- 2
- 3
- 4
- 5
- 6
- 7
- 8
- 9 (Excellent)
- Not assessable

Assessment based on:

Select all options that apply to you:

- EMD extractability Relevance of
- recommendation

Top 2 recommendations:

Which recommendations for "vaccination status" do you consider most suitable for measuring the quality of good use of the EPD in general practice?

1.

*

Choose one of the following options:

- Percentage of the population aged 7 years or older who have received all basic vaccinations. Percentage
- of the patient population aged 65 years and older who have received a flu vaccination. The EPD must
- contain information that the patient has not been vaccinated.
- The EPD must contain the date of administration for each vaccination.

2.

*

Select one of the following options:

- ◯ Percentage of the population aged 7 years or older who have received all basic vaccinations. Percentage
- ◯ of the patient population aged 65 years or older who have received a flu vaccination. The EPD must
- ◯ contain information that the patient has not been vaccinated.
- ◯ The EPD must contain the date of administration for each vaccination.

If you have any suggestions for recommendations that you believe are missing from the above list, please note them in the box below. Please always clearly state a recommendation and the reason for this recommendation.

Enter your answer here:

7. Patient's wishes

To what extent are the following recommendations relevant for measuring the quality of the proper use of the EMD in general practice in relation to the registration of **the patient's wishes** in the EPD?

Indicator / Recommendation	Source	Year	Level of evidence
**RECOMMENDATION:** **The reporting of discussions with the patient about treatment preferences and end-of-life care is primarily recorded in SOEP reports in an episode entitled 'End-of-life discussion/treatment preferences' (ICPC A20, version 2018).**	ADEPD, NHS, HASP	2019 2023, 2018	No gradation

Your rating:

*

Choose one of the following options:

- ◯ 1 (Poor)
- ◯ 2
- ◯ 3
- ◯ 4
- ◯ 5
- ◯ 6
- ◯ 7
- ◯ 8
- ◯ 9 (Excellent)
- ◯ Not assessable

Assessment based on:

Select all options that apply to you:

- ☐ EMD extractability Relevance of
- ☐ recommendation

Indicator / recommendation	Source	Year	Level of evidence
**RECOMMENDATION:** The final decisions on whether or not to treat in specific situations are recorded in the 'Treatment limits' section of the file. These treatment limits are: registration of cardiopulmonary resuscitation (CPR); admission to hospital, admission to intensive care, artificial ventilation, administration of a blood product, other treatment (in free text) or administration of antibiotics.	ADEPD	2019	No gradation

Your assessment:

*

Choose one of the following options:

- ◯ 1 (Poor)
- ◯ 2
- ◯ 3
- ◯ 4
- ◯ 5
- ◯ 6
- ◯ 7
- ◯ 8
- ◯ 9 (Excellent)
- ◯ Not assessable

Assessment based on:

Select all options that apply to you:

☐ EMD extractability Relevance of
☐ recommendation

Indicator / recommendation	Source	Year	Level of evidence
**RECOMMENDATION:** **Written declarations of intent, such as an euthanasia declaration or a do-not-resuscitate declaration, which the patient presents to their general practitioner, are added to the EPD as correspondence.**	**ADEPD**	**2019**	**No gradation**

Your opinion:

*

Choose one of the following options:

- ○ 1 (Poor)
- ○ 2
- ○ 3
- ○ 4
- ○ 5
- ○ 6
- ○ 7
- ○ 8
- ○ 9 (Excellent)
- ○ Not assessable

Assessment based on:

Select all options that apply to you:

☐ EMD extractability Relevance of
☐ recommendation

Indicator / recommendation	Source	Year	Level of evidence
**RECOMMENDATION:**<br><br>**Are there any goals for person-centred care (Individual Care Plan = ICP) recorded in the file? Person-centred care focuses on the patient's goals. The patient determines the goals for their care together with their care providers. These goals are often not medically oriented, but they do have points of contact with medicine.**<br><br>**For example: a COPD patient wants to be able to go for a walk outside every day and chat with the neighbours. Proper management of COPD in combination with walking and breathing training can make this goal achievable.**<br>**This requires coordination between the general practitioner (COPD management), the somatic care nurse (regular COPD check-ups) and a specialised physiotherapist (walking and breathing training).**<br>**The IZP provides clarity about who does what and who is responsible for what.**	**ADEPD**	**2019**	**No gradation**

Your assessment:

*

Choose one of the following options:

- 1 (Poor)
- 2
- 3
- 4
- 5
- 6
- 7
- 8
- 9 (Excellent)
- Not assessable

Assessment based on:

Select all options that apply to you:

- [ ] EMD extractability Relevance of
- [ ] recommendation

Indicator / recommendation	Source	Year	Level of evidence
**RECOMMENDATION:**<br>The EPD should contain information about the screening programmes applicable to each patient or the patient's wishes regarding this screening.<br>In Belgium, the following population screening programmes are available: breast cancer, cervical cancer, colon cancer, congenital disorders. (Types of population screening | Population screening)<br>Note: this concerns the acceptance/rejection of care approaches and not the presence of individual plans (as recommended in topic 4).	ADEPD	2019	No gradation

Your opinion:

*

Choose one of the following options:

- ◯ 1 (Poor)
- ◯ 2
- ◯ 3
- ◯ 4
- ◯ 5
- ◯ 6
- ◯ 7
- ◯ 8
- ◯ 9 (Excellent)
- ◯ Not assessable

Assessment based on:

Select all options that apply to you:

☐ EMD extractability Relevance of
☐ recommendation

Top 3 recommendations:

Which recommendations for the "patient's wishes" do you consider most appropriate for measuring the quality of proper use of the EPD in general practice?

1.

*

Choose one of the following options:

◯ The reporting of discussions with the patient about treatment wishes and end-of-life care is primarily recorded in SOEP reports in an episode entitled 'End-of-life discussion/treatment wishes' (ICPC A20, version 2018).

◯ The final decisions on whether or not to treat in specific situations are recorded in the "Treatment limits" section of the file.

◯ Written declarations of intent, such as an euthanasia declaration or a do-not-resuscitate declaration, which the patient presents to the GP, are added to the EPD as correspondence.

◯ Are there any goals for person-centred care (Individual Care Plan = ICP) recorded in the file?

◯ The EPD must contain information about the screening procedures applicable to each patient or the patient's wishes regarding this screening.

2.

*

Choose one of the following options:

◯ The reporting of discussions with the patient about treatment wishes and end-of-life care is primarily recorded in SOEP reports in an episode entitled 'End-of-life discussion/treatment wishes' (ICPC A20, version 2018).

◯ The final decisions on whether or not to treat in specific situations are recorded in the 'Treatment limits' section of the file.

◯ Written declarations of intent, such as an euthanasia declaration or a do-not-resuscitate declaration, which the patient presents to the GP, are added to the EPD as correspondence.

◯ Are there any goals for person-centred care (Individual Care Plan = ICP) recorded in the file?

◯ The EPD must contain information regarding the screening procedures applicable to each patient or the patient's wishes regarding this screening.

3.

*

Choose one of the following options:

◯ The reporting of discussions with the patient about treatment wishes and end-of-life care is primarily recorded in SOEP reports in an episode entitled 'End-of-life discussion/treatment wishes' (ICPC A20, version 2018).

◯ The final decisions on whether or not to treat in specific situations are recorded in the 'Treatment limits' section of the file.

◯ Written declarations of intent, such as an euthanasia declaration or a do-not-resuscitate declaration, which the patient presents to the GP, are added to the EPD as correspondence.

◯ Are there any goals for person-centred care (Individual Care Plan = ICP) recorded in the file?

◯ The EPD must contain information regarding the screening procedures applicable to each patient or the patient's wishes regarding this screening.

If you have any suggestions for recommendations that you believe are missing from the above list, please note them in the box below. Please clearly state your recommendation and the reason for it.

Enter your answer here:

The end

Thank you for participating in the first part of the survey to develop a set of indicators for the correct use of electronic patient records by general practitioners. We hope to see you at the panel discussion on 5 November 2024 at 8 p.m. You will receive a reminder email.

Date	What?	How?	Duration?
30 September 2024, 11:59 p.m.	Deadline for completing the questionnaire	Online survey	30 min
5 November 2024, 8:00 p.m.	Panel discussion	Online	120 min
30 November 2024 23:59	Deadline for reviewing final list of indicators	Email	15 min

If you have any suggestions for recommendations that you think are missing from the above list, please note them in the box below. Please always clearly state a recommendation and your reasons for this recommendation.

Enter your answer here:

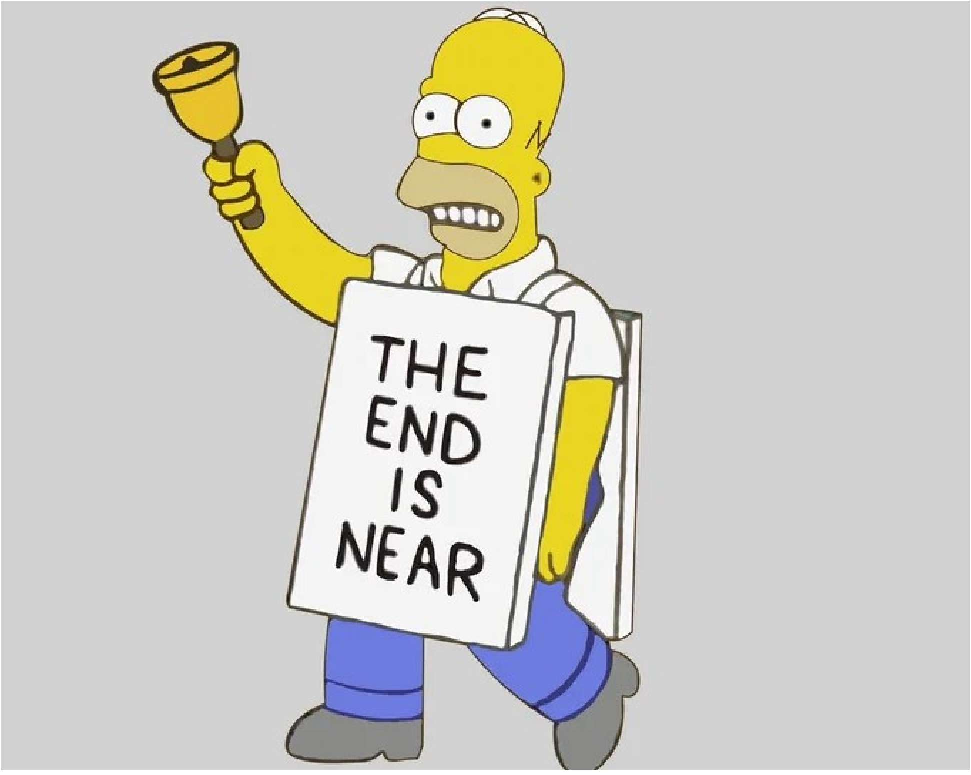

16-11-2024 – 17:01

Submit your survey.

Thank you for participating in this survey.
