# Supplementary material for: Development of Quality Indicators for the Correct Use of Electronic Medical Records in Primary Care: Modified Delphi Study
Source: JMIR Med Inform. 2026 Jan 19;14:e80057. doi: 10.2196/80057 (PMC12865340; doi:10.2196/80057)
Supplement: Multimedia Appendix 5 [file medinform_v14i1e80057_app5.pdf]

Quality indicators for the correct use of electronic medical records in primary care

Kwaliteitsindicatoren en aanbevelingen voor het correct gebruik van het elektronisch patiëntendossier bij huisartsen

Indicateurs de qualité et recommandations pour le bon usage du dossier médical informatisé chez les médecins généralistes

#	Omschrijving (NL)	Description (FR)	Definition and classification
**Topic 1.**	**Volledigheid en adequaatheid probleemlijst**	**Exhaustivité et adéquation de la liste des problèmes**	**Completeness and adequacy of the problem list**
**1.1**	Hoeveel actieve zorgelementen heeft een patiënt gemiddeld op zijn/haar lijst met zorgelementen?	Combien d'éléments de santé/soins actifs un patient a en moyenne sur sa liste d'éléments de santé/soins ?	The mean of active problem items listed on a patient’s problem list.
**1.2**	Percentage van de episodes op de episodelijst heeft een gecodeerde diagnose?	Pourcentage d'éléments de santé/soins de la liste d'éléments de santé/soins avec un code diagnostique.	Percentage of items on the problem list that are linked to an encoded diagnosis
**1.3**	Percentage van patiënten met medicatie voor schildklierlijden in de medicatielijst met een gecodeerde diagnose schildklierlijden?	Pourcentage des patients avec des médicaments pour une maladie thyroïdienne dans la liste des médicaments associés à un code diagnostique d'une maladie thyroïdienne.	Percentage of patients with medication for thyroid disease in their medication list with an encoded diagnosis of thyroid disease on the problem list.
**1.4**	Percentage van patiënten met medicatie voor epilepsie in de medicatielijst met een gecodeerde diagnose epilepsie?	Pourcentage des patients avec des médicaments pour l'épilepsie dans la liste des médicaments associés à un code diagnostique d'épilepsie.	Percentage of patients with medication for epilepsy in their medication list with an encoded diagnosis of epilepsy on the problem list.
**1.5**	Percentage van patiënten met medicatie voor ziekte van Parkinson in de medicatielijst met een gecodeerde diagnose ziekte van Parkinson?	Pourcentage des patients avec des médicaments pour la maladie de Parkinson dans la liste des médicaments associés à un code diagnostique de la maladie de Parkinson.	Percentage of patients with medication for Parkinson's disease in their medication list with an encoded diagnosis of Parkinson's disease on the problem list.
**1.6**	Percentage van patiënten met medicatie voor depressie in de medicatielijst met een gecodeerde diagnose depressie?	Pourcentage des patients avec des médicaments pour la dépression dans la liste des médicaments associés à un code diagnostique de dépression.	Percentage of patients with medication for depression in their medication list with an encoded diagnosis of depression on the problem list.
**1.7**	Percentage van patiënten met medicatie voor cardiovasculaire ziekte in de medicatielijst met een gecodeerde diagnose cardiovasculaire ziekte?	Pourcentage des patients avec des médicaments pour une maladie cardiovasculaire dans la liste des médicaments associés à un code diagnostique d'une maladie cardiovasculaire.	Percentage of patients with medication for cardiovascular disease in their medication list with an encoded diagnosis of cardiovascular disease on the problem list.

**1.8**	Percentage van patiënten met medicatie voor astma/COPD in de medicatielijst met een gecodeerde diagnose astma/COPD?	Pourcentage des patients avec des médicaments pour asthme/COPD dans la liste des médicaments associés à un code diagnostique d'asthme/BPCO.	Percentage of patients with medication for asthma/chronic obstructive pulmonary disease in their medication list with an encoded diagnosis of asthma/chronic obstructive pulmonary disease on the problem list.
**1.9**	Percentage van patiënten met medicatie voor diabetes in de medicatielijst met een gecodeerde diagnose diabetes?	Pourcentage des patients avec des médicaments pour le diabète dans la liste des médicaments associés à un code diagnostique de diabète.	Percentage of patients with medication for diabetes in their medication list with an encoded diagnosis of diabetes on the problem list.
**1.10**	Percentage van gecodeerde items op de probleemlijst met een bijkomende beschrijving of opmerking.	Pourcentage d'éléments codés dans la liste des problèmes accompagnés d'une description ou d'une remarque supplémentaire.	Percentage of encoded items in the problem list with an additional description or comment.
**1.11**	Percentage van gecodeerde items op de probleemlijst met een registratie van een vermoedelijke of effectieve startdatum.	Pourcentage d'éléments codés dans la liste des problèmes avec enregistrement d'une date de début présumée ou effective.	Percentage of encoded items in the problem list for which the estimated or actual date of onset has been registered.
**1.12**	Percentage van inactieve gecodeerde items op de probleemlijst met een registratie van een vermoedelijke of effectieve einddatum.	Pourcentage d'éléments codés inactifs dans la liste des problèmes avec enregistrement d'une date de fin présumée ou effective.	Percentage of inactive items on the problem list for which the estimated or actual date the condition was resolved has been registered
**1.13**	Percentage van items op de lijst met operaties en procedures die gecodeerd geregistreerd zijn.	Pourcentage d'éléments de la liste des opérations et procédures avec un code diagnostique.	Percentage of surgeries and other procedures that have been registered as an encoded item.
**1.14**	Percentage van items op de lijst met operaties en procedures die gelinkt zijn met een gerelateerde zorgepisode.	Pourcentage d'éléments sur la liste des opérations et procédures connecté à un épisode de soins.	Percentage of surgeries and other procedures in the patient history that have been linked to the related episode of care.
**1.15**	Percentage van items op de lijst met operaties en procedures met een bijkomende beschrijving of opmerking.	Pourcentage d'éléments de la liste des opérations et procédures accompagnés d'une description ou d'une remarque supplémentaire.	Percentage of surgeries and other procedures in the patient's history with an additional description or comment.
**1.16**	Percentage van items op de lijst met operaties en procedures met een datum en/of tijd waarop de operatie of ingreep plaats vond; of gepland is.	Pourcentage d'éléments figurant sur la liste des opérations et procédures avec une date et/ou une heure à laquelle l'opération ou l'intervention a eu lieu ou est prévue.	Percentage of surgeries and other procedures in the patient summary for which the date and or time on which the procedure was or is intended to be performed is registered.
**Topic 2.**	**Gestructureerd registreren in EPD**	**Enregistrement structuré dans le DMI**	**Encoded registration in the EMR**
**2.1**	Hoeveel deelcontacten worden er gemiddeld per contact aangemaakt?	Combien de sous-contacts sont enregistrés en moyenne par contact ?	The mean number of sub-contacts created per contact during the observation period.
**2.2**	Percentage van deelcontacten in het journaal gekoppeld aan zorgelementen met een gecodeerde diagnose?	Pourcentage de sous-contacts pendant la période d'observation associés à un épisode de soins.	Percentage of sub-contacts during the observation period connected with an episode of care.

**2.3**	Percentage de deelcontacten die zijn vastgelegd tijdens consulten, telefonische contacten en huisbezoeken hebben een Subjectief-, Objectief-, Evaluatie- of Planning-regel?	Pourcentage de contacts partiels enregistrés lors de consultations, de contacts téléphoniques et de visites à domicile comportant une ligne « Subjectif », « Objectif », « Évaluation » ou « Planification ».	Percentage of sub-contacts (registered as consultation, home visit, or teleconsultation) with a Subjective-, Objective-, Evaluation- and Planning-item.
**2.4**	Hoeveel veranderingen zijn er in status van planningen (procedures/handelingen) per observatieperiode per 1000 GMD patiënten?	Quel est le nombre de changements de statut de planification (procédures/actions) pour 1 000 patients ayant un dossier médical global, par période d'observation ?	The number of patients with a Global Medical Record in the practice with changes in the status of tasks (procedures/actions) over the total number of patients with a Global Medical Record in the practice.
**Topic 3.**	**Compleetheid en actualiteit medicatie-overzicht**	**Exhaustivité et actualisation de la liste des médicaments**	**Completeness and actuality of the medication list**
**3.1**	Percentage van de medicatie op de lijst ‘actuele medicatie’ is onterecht als actuele medicatie gelabeld?	Pourcentage de médicaments sur la liste des « médicaments actuels » incorrectement étiquetés comme tels.	Percentage of medications on the medication list labelled as 'active', that are no longer active.
**3.2**	Percentage van de medicatie is gekoppeld aan een zorgelement?	Pourcentage de médicaments connectés à un élément de soins/santé.	Percentage of active medications on the medication list that are linked to a health condition on the problem list.
**3.3**	Percentage van actieve medicatie met een volledige dosis- en behandelingsschema?	Pourcentage de médicaments actifs avec une enregistrement complet de la posologie et du schéma thérapeutique.	Percentage of active medications on the medication list for which the dosage and treatment regimen are complete.
**Topic 4.**	**Risicofactoren / Medicatiebewaking**	**Facteurs de risque / surveillance des médicaments**	**Risk factors/drug monitoring**
**4.1**	Percentage van patiënten met minimum één geregistreerde geneesmiddelenallergie of -intolerantie?	Pourcentage de patients ayant au minimum une enregistrement d'une allergie ou d'une intolérance aux médicaments.	Percentage of patients with at least one registration for a drug allergy or intolerance.
**4.2**	Percentage van patiënten met een registratie van een risicofactor (profylaxe voor Addison crisis, endocarditis, bloedingsziekten, endoprothese, immuun gecompromitteerde patiënt, (functionele) asplenie, trombose of bijzonder resistente micro-organismen)?	Pourcentage de patients avec un enregistrement d'un facteur de risque (prophylaxie de la crise d'Addison, endocardite, maladies hémorragiques, endoprothèse, patient immunodéprimé, asplénie fonctionnelle, thrombose ou micro-organismes particulièrement résistants) ?	Percentage of patients with a registration for a risk factor. Risk factors are clinical information that is imperative to know so that the life or health of the patient is not threatened. For example: prophylaxis for adrenal crisis (Addison's disease), endocarditis, bleeding disorders, endoprosthesis, immunocompromised patients, (functional) asplenia, thrombosis or particularly resistant micro-organisms.

**4.3**	Percentage van patiënten met minimum één gecodeerde registratie van een item in het lichamelijk onderzoek (gewicht, lengte, bloeddruk, hartritme…)	Pourcentage de patients ayant au moins un enregistrement codé pour un examen physique (poids, taille, pression artérielle, pouls…)	Percentage of patients with at least one encoded registration for a physical examination (weight, height, blood pressure, pulse…)
**4.4**	Percentage van patiënten met minimum één registratie van een item voor sociale anamnese.	Pourcentage de patients ayant au moins un enregistrement d'un élément pour l'anamnèse sociale.	Percentage of patients with at least one registration of an item for social history.
**4.5**	Percentage patiënten van 12 jaar en ouder waarvoor ten minste één vermelding bestaat met betrekking tot het gebruik van sigaretten, alcohol en drugs	Pourcentage de patients âgés de 12 ans et plus pour lesquels il existe au moins une mention concernant la consommation de cigarettes, d'alcool et de substances	Percentage of patients 12 years and older for whom there is at least one notation concerning the use of cigarettes, alcohol, and substances.
**4.6**	Percentage patiënten met ten minste één registratie van een item voor familiegeschiedenis.	Pourcentage de patients ayant au moins un élément enregistré dans leurs antécédents médicaux familiaux.	Percentage of patients with at least one registration of an item for family medical history
**Topic 5.**	**Patiëntenidentificatie / Contactinformatie**	**Identification du patient /informations de contact**	**Patient identification and contact information**
**5.1**	Percentage patiënten met een registratie van welke urgentiedienst (ziekenhuis) de patiënt gekend is.	Pourcentage de patients pour lesquels le service des urgences de l'hôpital dans lequel le patient est connu est enregistré.	Percentage of patients with a registration for hospital preference.
**5.2**	Percentage patiënten met minimum één registratie van de contactpersonen, mantelzorgers of als vertegenwoordiger aangewezen personen.	Pourcentage de patients ayant enregistré au moins une fois les coordonnées de leurs contacts, des aidants informels et des représentants légaux (si nécessaire).	Percentage of patients with at least one registration of contact information for a contact person, caregiver or person designated as representative.
**5.3**	Percentage patiënten met minimum één registratie van een zorgverlener in het zorgteam dat de patiënt opvolgt.	Pourcentage de patients ayant au moins un enregistrement d'un prestataire de soins dans l'équipe soignante qui suit le patient.	Percentage of patients with at least one registration of a healthcare professional in his/her care team.
**5.4**	Percentage patiënten met minimum één registratie van: adres patiënt, e-mailadres, werkgever, huis en werk telefoonnummer, burgerlijke staat, registratie op patiëntenplatform en inwonenden bij de patiënt.	Pourcentage de patients ayant au moins un enregistrement: l'adresse du patient, son adresse e-mail, son employeur, ses numéros de téléphone à domicile et sur son lieu de travail, sa situation de famille, son inscription à une plateforme de patients ainsi que les coordonnées des personnes et des animaux qui vivent avec lui.	Percentage of patients with at least one registration for: address, e-mail, employer, home and work phone, marital status, registration status on patient platform or co-housing people and animals?
**Topic 6.**	**Vaccinatiestatus**	**Statut vaccinal**	**Vaccination status**
**6.1**	Percentage van patiënten met leeftijd 7 jaar en ouder die alle basisvaccinaties hebben gekregen.	Pourcentage de patients âgés de 7 ans et plus ayant reçu les vaccinations primaires recommandées pour les enfants.	Percentage of patients 7 years and older who have a registration for all the recommended primary childhood vaccines.

**6.2**	Percentage van patiënten met leeftijd 65 jaar en ouder die een griepvaccinatie hebben gekregen.	Pourcentage de patients âgés de 65 ans et plus ayant reçu le vaccin contre la grippe.	Percentage of patients 65 years and older who have a registration for a yearly flu vaccine.
**6.3**	Percentage vaccinaties waarvan de datum waarop de vaccinatie is toegediend, is geregistreerd.	Pourcentage de vaccinations pour lesquelles la date d'administration du vaccin est enregistrée.	Percentage of vaccinations for which the date the vaccination was administered is registered.
**Topic 7.**	**Wil van de patiënt**	**Volonté du patient**	**Patient's choices**
**7.1**	Percentage patiënten met een registratie van behandelingsvoorkeuren: verzoek om euthanasie of weigering van intubatie, reanimatie, orgaandonatie, vaccinatie of bloedtransfusie.	Pourcentage de patients ayant enregistré leurs préférences en matière de traitement : demande d'euthanasie ou refus d'intubation, de réanimation, de don d'organes, de vaccination ou de transfusion sanguine.	Percentage of patients with a registration of treatment preferences: euthanasia request or refusal for intubation, resuscitation, organ donation, vaccination or blood transfusion.
**7.2**	Percentage patiënten met ten minste één contact met een subjectief, objectief, evaluatie- of planningsitem dat gekoppeld is aan een gecodeerde diagnose met betrekking tot behandelingsvoorkeuren of zorg aan het levenseinde.	Pourcentage de patients ayant eu au moins un contact avec un élément subjectif, objectif, d'évaluation ou de planification lié à un diagnostic codé concernant les préférences en matière de traitement ou les soins de fin de vie.	Percentage of patients with at least one contact with a Subjective-, Objective-, Evaluation- or Planning-item linked to an encoded diagnosis regarding treatment preferences or end-of-life care.
**7.3**	Percentage patiënten met ten minste één registratie van een doel voor gepersonaliseerde zorg (*Goal-Oriented Care*).	Pourcentage de patients ayant enregistré au moins un objectif de soins personnels (Goal-Oriented Care).	Percentage of patients with at least one registration of a goal for personalised care (Goal-Oriented Care).
**7.4**	Percentage patiënten met ten minste één registratie met betrekking tot wensen van de patiënt betreffende screeningstrajecten.	Pourcentage de patients ayant au moins une inscription concernant les souhaits du patient concernant le dépistage.	Percentage of patients with at least one registration regarding preferences for preventive healthcare.
